# 1,5-Benzodiazepin-2(3H)-ones: In Vitro Evaluation as Antiparkinsonian Agents

**DOI:** 10.3390/antiox10101584

**Published:** 2021-10-09

**Authors:** Ana Ortíz de Zárate, Marta Pérez-Torralba, Iñigo Bonet Isidro, Concepción López, Rosa M. Claramunt, Diana Martínez-Casanova, Isabel Sánchez-Vera, Jesús Jiménez-González, José Luis Lavandera

**Affiliations:** 1 Instituto de Medicina Molecular Aplicada (IMMA), Facultad de Medicina, Universidad San Pablo-CEU, CEU Universities, Campus de Montepríncipe, E-28668 Madrid, Spain; ana.ortizzarate.ce@ceindo.ceu.es (A.O.d.Z.); ini.bonet.ce@ceindo.ceu.es (I.B.I.); dia.martinez.ce@ceindo.ceu.es (D.M.-C.); isanver@ceu.es (I.S.-V.); jesuscanillo@hotmail.com (J.J.-G.); 2 Departamento de Química Orgánica y Bio-Orgánica, Facultad de Ciencias, Universidad Nacional de Educación a Distancia (UNED), Paseo Senda del Rey 9, E-28040 Madrid, Spain; mtaperez@ccia.uned.es (M.P.-T.); clopez@ccia.uned.es (C.L.); 3 Department of Pharmacology, Physiology and Neuroscience, Medical School, Rutgers, The State University of New Jersey, 185 South Orange Avenue, Newark, NJ 07103, USA

**Keywords:** neurodegeneration, Parkinson disease, oxidative stress, mitochondrial dysfunction, neuroprotectant, antioxidant, 1,5-Benzodiazepin-2(3H)-ones, 6-OHD, MPP^+^, drug-like properties

## Abstract

A new series of twenty-three 1,5-benzodiazepin-2(3H)-ones were synthesized and evaluated in the 2,2′-azino-bis(3-ethylbenzothiazoline-6-sulfonic acid (ABTS), ferric reducing antioxidant power (FRAP), and 2,2-diphenyl-1-picrylhydrazyl (DPPH) assays as a new chemotype with antioxidant and good drug-like properties. All of the derivatives showed low cytotoxicity in comparison to curcumin against the human neuroblastoma SH-SY5Y and the human hepatoma HepG2 cell lines. Experimental solubility in bio-relevant media showed a good relationship with melting points in this series. Five compounds with the best antioxidant properties showed neuroprotectant activity against H_2_O_2_-induced oxidative stress in the SH-SY5Y cell line. From them, derivatives 4-phenyl-1H-1,5-benzodiazepin-2(3H)-one (**18**) and 4-(3,4,5-trimethoxyphenyl)-1H-1,5-benzodiazepin-2(3H)-one (**20**) yielded good neuroprotection activity in the same neuronal cell line under 6-OHD and MPP^+^ insults as in vitro models of mitochondrial dysfunction and oxidative stress in Parkinson’s disease (PD). Both compounds also demonstrated a significant reduction of intracellular Reactive Oxygen Species (ROS) and superoxide levels, in parallel with a good improvement of the Mitochondrial Membrane Potential (ΔΨm). Compared with curcumin, compound **18** better reduced lipid peroxidation levels, malondialdehyde (MDA), in SH-SY5Y cells under oxidative stress pressure and recovered intracellular glutathione synthetase (GSH) levels. Apoptosis and caspase-3 levels of SH-SY5Y under H_2_O_2_ pressure were also reduced after treatment with **18**. Neuroprotection in neuron-like differentiated SH-SY5Y cells was also achieved with **18**. In summary, this family of 1,5-benzodiazepin-2-ones with an interesting antioxidant and drug-like profile, with low cytotoxic and good neuroprotectant activity, constitutes a new promising chemical class with high potential for the development of new therapeutic agents against PD.

## 1. Introduction

Although there is a huge improvement in life expectancy, neurodegenerative diseases such as Alzheimer’s disease (AD) and Parkinson’s disease (PD) are a serious burden in addition to other neurological disorders which are now the leading source of disability globally [1,2,3,4]. Age is the most important risk factor [3], but other risks seem to be associated with industrial exposure to chemicals and pollutants, pesticides, solvents, and metals [5,6,7,8].

The most significant pathological changes in PD patients are the progressive degeneration of dopaminergic neurons in the substantia nigra, depletion of dopamine, and the intraneuronal inclusions of protein aggregates, mainly of α-synuclein [9,10] which are called Lewy bodies [11]. An important therapeutic strategy for PD treatment is the administration of levodopa as a dopamine precursor [12,13]. Levodopa crosses the blood–brain barrier and reaches its target site in the brain, where it is decarboxylated to dopamine and stored in presynaptic striatal neurons [14]. Unfortunately, after 4–6 years of treatment, 40% of PD patients treated with levodopa develop motor fluctuations and dyskinesias [15,16]. Despite the risk of developing motor-related undesired effects, levodopa is still the drug of choice. Dopamine agonists such as ropinirole and pramipexole have been explored as alternatives for PD treatment [17,18]. However, after several clinical trials using the dopamine agonists, no significant advantage was seen in slowing the progression of motor symptoms, and most of them have peripheral and central side-effects, which are often the reason for their discontinuation [19,20,21]. Other therapeutic approaches explored are dopamine metabolism inhibitors and non-dopaminergic drugs, albeit without success [22,23,24].

Substantial evidence links pathophysiological alterations observed in sporadic PD to some associated genes that may be involved in the disruption of mitochondrial integrity, triggering mitochondrial dysfunction, oxidative stress, and an increase in protein misfolding and aggregation [25,26]. Those facts reinforce the significance of oxidative stress and mitochondrial dysfunction as mechanisms of neuronal degeneration, offering unique opportunities to pursue research on new therapeutic interventions [27,28,29].

An excessive formation of reactive oxygen and nitrogen species (ROS and RNS) produces oxidative stress that overcomes the cell antioxidant defenses, mainly sustained by antioxidant enzymes such as superoxide dismutase (SOD), catalase, and glutathione peroxidase, and endogenous antioxidants such as ascorbic acid, alpha-tocopherol, glutathione (GSH), carotenoids, and flavonoids [30,31]. Failure of the endogenous cellular antioxidant system leads to oxidative damage of proteins, lipids, and DNA/RNA, which are the common features of many neurodegenerative diseases [30,32].

ROS are produced either by an enzyme or metal-catalysed processes or in the mitochondrial electron transport chain during energy transduction [33,34,35]; the non-radical hydrogen peroxide (H_2_O_2_), the superoxide radical (O2^•−^), and the hydroxyl radical (OH^•^) are the most important ones [36,37,38]. Consequently, antioxidant compounds are key strategies to fight oxidative stress and mitochondrial dysfunction in neurodegenerative diseases [39,40]. The beneficial effects of antioxidants have been demonstrated in in vitro and in vivo models of PD [29,41,42]. The most widely studied antioxidant therapies of these have been vitamin E [43,44], vitamin C [45,46,47], Coenzyme Q10 [48,49,50], melatonin [51,52,53], N-acetylcysteine [54,55,56], curcumin, and other phenolic and flavonoid compounds [57,58].

**Figure 1:**
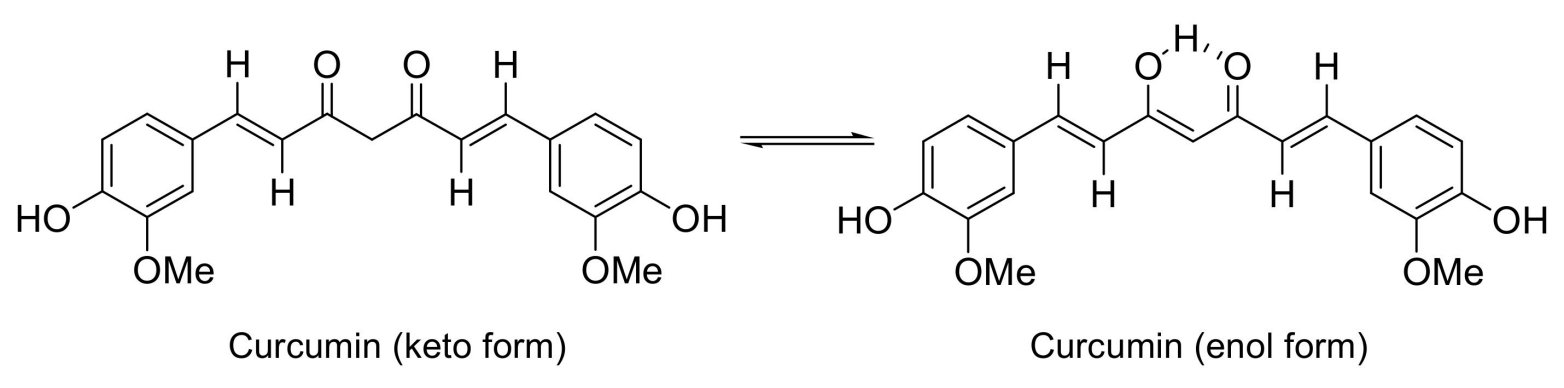
Curcumin presented in its two possible tautomeric forms.

Curcumin (Figure 1) and curcuminoids prevent α-synuclein aggregation in PD and attenuate ROS-induced neurodegeneration mediated by DJ-1, LRRK-2, and PINK-1 genes [59,60,61,62], pointing out that they are interesting therapeutic tools for PD treatment. However, curcumin derivatives have poor physiochemical and drug-like properties due to their low aqueous solubility and chemical stability, showing poor absorption, low bioavailability, and low metabolic stability after oral administration [63,64,65,66]. All of these features make these compounds unsuitable from the pharmacokinetic and pharmacodynamic point of view, hindering their effective targeting to cells and mitochondria in the human brain [63,64,65,67]. Even with those promising results in animal and in vitro cell studies, curcumin has no proven benefit for its use in PD from large randomized controlled trials [68,69,70].

Nevertheless, curcuminoids are interesting chemotypes amenable to chemical modifications that can improve their drug-like profile. Most of the previously reported synthetic work focused on the development of new symmetric curcuminoids with a similar antioxidant profile as curcumin but with different aromatic substitution patterns [71,72,73]. However, only a few attempts have been developed recently to explore asymmetrical substitution patterns of curcumin [74,75,76].

**Figure 2:**
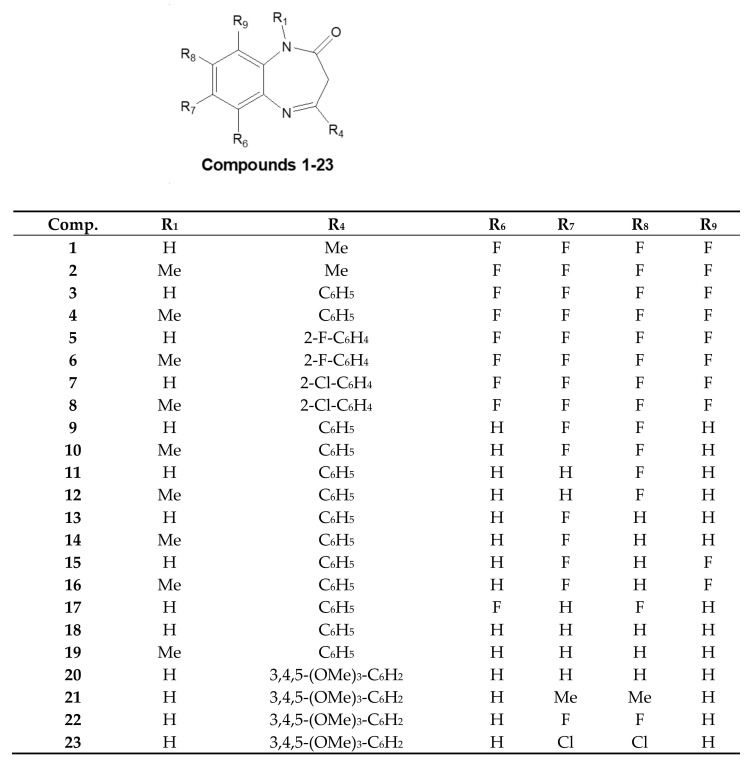
The studied 1,5-benzodiazepin2-(3*H*)-ones.

Taking the above into account, the present work reports the synthesis and biological evaluation of new 1,5-benzodiazepin-2(3H)-one (Figure 2) derivatives with antioxidant and in vitro cell neuroprotective properties with an improved drug-like profile as an interesting chemotype for the development of new neuroprotectant compounds for PD.

## 2. Materials and Methods

### 2.1. General

All chemicals cited in the synthetic procedures were commercial compounds. Melting points were determined by Differential Scanning Calorimetry (DSC) with a DSC 220 C instrument (SEIKO Instruments Inc., Torrance, CA, USA) connected to a model SSC5200H disk station. Thermograms (sample size 0.003–0.005 g) were recorded with a scan rate of 5.0 °C. Column chromatography was performed on silica gel 60, 70–230 mesh (Merck, Madrid, Spain), and elemental analyses were performed using a LECO-CHNS-932 (apparatus (LECO INSTRUMENTOS S.L., Madrid, Spain).

.

### 2.2. NMR Parameters

Solution spectra were recorded on a 9.4 Tesla spectrometer (Bruker Española S.A., Madrid, Spain, 400.13 MHz for ^1^H, 376.50 MHz for 19F, and 100.62 MHz for ^13^C and 40.54 MHz for ^15^N) at 300 K with a 5 mm inverse detection H-X probe equipped with a z-gradient coil or with a Quattro Nucleus Probe (QNP), i.e., 5 mm probe (^19^F). Chemical shifts (δ in ppm) are presented relative to the internal solvent: DMSO-d_6_ 2.49 for 1H and 39.5 for 13C. External references were used for ^15^N and ^19^F, nitromethane, and CFCl_3_.

**Figure 3:**
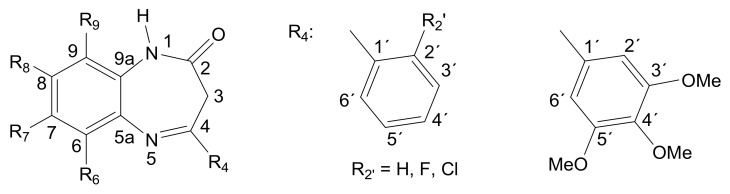
Atom numbering used for the NMR assignments.

Assignments according to the atom numbering depicted in Figure 3 were achieved by means of 2D NMR spectra: (^1^H-^1^H) gs-NOESY (gs-Nuclear Overhauser Effect spectroscopy), (^1^H-^13^C) gs-HMQC (gs-Heteronuclear Multiple Quantum Coherence), (^1^H-^13^C) gs-HMBC (gs-Heteronuclear Multiple Bond Correlation), (^1^H-^15^N) gs-HMQC, (^1^H-^15^N) gs-HMBC, (^19^F-^19^F) gs-COSY (gs-Homonuclear Correlation Spectroscopy), and (^1^H-^19^F) gs-HOESY (gs-Heteronuclear Overhauser Enhancement Spectroscopy). In some cases, to obtain the coupling constants involving the fluorine nuclei, irradiation of protons proved to be necessary.

### 2.3. Preparation of Compounds **1**–**23**

6,7,8,9-Tetrafluoro-4-methyl-1H-1,5-benzodiazepin-2(3H)-one (**1**, m.p. 155.2 °C) [77], 6,7,8,9-tetrafluoro-1,4-dimethyl-1H-1,5-benzodiazepin-2(3H)-one (**2,** m.p. 132.2 °C) [77], 6,7,8,9-tetrafluoro-4-phenyl-1H-1,5-benzodiazepin-2(3H)-one (**3**, m.p. 253.3 °C) [78], 6,7,8,9-tetrafluoro-4-phenyl-1-methyl-1H-1,5-benzodiazepin-2(3H)-one (**4**, m.p. 133.7 °C) [78], 6,7,8,9-tetrafluoro-4-(2-fluorophenyl)-1H-1,5-benzodiazepin-2(3H)-one (**5**, m.p. 248.1 °C) [78], 6,7,8,9-tetrafluoro-4-(2-fluorophenyl)-1-methyl-1H-1,5-benzodiazepin-2(3H)-one (**6**, m.p. 142.6 °C) [78], 6,7,8,9-tetrafluoro-4-(2-chlorophenyl)-1H-1,5-benzodiazepin-2(3H)-one (**7**, m.p. 211.7 °C) [78], and 6,7,8,9-tetrafluoro-4-(2-chlorophenyl)-1-methyl-1H-1,5-benzodiazepin-2(3H)-one (**8**, m.p. 128.7 °C) [78], were prepared as described previously and were purified by recrystallization in ethanol.

#### 2.3.1. 7,8-Difluoro-4-phenyl-1H-1,5-benzodiazepin-2(3H)-one (**9**)

4,5-Difluorobenzene-1,2-diamine (0.50 g, 3.47 mmol) and ethyl benzoylacetate (0.62 mL, 3.57 mmol) were heated at 120 °C in anhydrous xylene (5 mL) for 3 h 30 min. The mixture was cooled, and then a solid precipitated. This residue was washed with ethyl ether to yield compound 9 (0.59 g, 64%) as a pale yellow solid: m.p. 214.2 °C (EtOH); ^1^H NMR (400.13 MHz, DMSO-d_6_) δ 10.62 (s, 1H, H1), 8.05 (dd, ^3^J = 8.0, ^4^J = 1.5 Hz, 2H, H2′-H6′), 7.53 (m, 3H, H3′-H5′, H4′), 7.47 (dd, ^4^JF8 = 11.8, ^3^JF7 = 8.5 Hz, 1H, H6), 7.18 (dd, ^4^JF7 = 11.7, ^3^JF8 = 8.0 Hz, 1H, H9), 3.56 (s, 2H, H3); ^13^C NMR (100.62 MHz, DMSO-d_6_) δ 165.9 (C2), 159.5 (C4), 147.0 (dd, ^1^JF = 245.6, ^2^JF = 14.1 Hz, C8), 145.7 (dd, ^1^JF = 242.8, ^2^JF = 13.8 Hz, C7), 136.8 (C1′), 136.3 (dd, ^3^JF = 8.7, ^4^JF = 2.3 Hz, C5a), 131.3 (C4′), 128.7 (C3′–C5′), 127.7 (C2’–C6’), 126.9 (dd, ^3^JF = 8.8, ^4^JF = 1.9 Hz, C9a), 115.4 (d, ^2^JF = 18.2 Hz, C6), 109.8 (d, ^2^JF = 20.1 Hz, C9), 39.8 (C3); ^15^N NMR (40.54 MHz, DMSO-d_6_) δ–242.2 (N1),–70.4 (N5); ^19^F NMR (376.50 MHz, DMSO-d_6_) δ–143.0 (m, F7),–140.3 (m, F8); Anal. Calcd for C_15_H_10_F_2_N_2_O: C, 66.18; H, 3.70; N, 10.29. Found: C, 65.82; H, 3.79; N, 10.08.

#### 2.3.2. 7,8-Difluoro-1-methyl-4-phenyl-1H-1,5-benzodiazepin-2(3H)-one (**10**)

A solution of 7,8-difluoro-4-phenyl-1H-1,5-benzodiazepin-2(3H)-one (0.40 g, 1.51 mmol) in DMF (2 mL) was heated at 110 °C in the presence of iodomethane (0.10 mL, 1.66 mmol), K_2_CO_3_ (0.25 g, 1.82 mmol) and a catalytic quantity of KI for 2 h 30 min. The mixture was cooled, treated with cold water, and extracted with ethyl acetate. The ethyl acetate was evaporated and the crude extract was purified by column chromatography (hexane/ethyl acetate, 10:1) to yield compound 10 (0.43 g, 83%). m.p. 115.8 °C (EtOH). ^1^H NMR (400.13 MHz, DMSO-d_6_) δ, 8.07 (dd, ^3^J = 8.1, ^4^J = 1.6 Hz, 2H, H2′–H6′), 7.55 (m, 3H, H3′–H5′, H4′), 7.69 (dd, ^4^JF7 = 12.5, ^3^JF8 = 8.0 Hz, 1H, H9), 7.44 (dd, ^4^JF8 = 11.7, ^3^JF7 = 8.7 Hz, 1H, H6), 4.16 (d, ^2^J = 11.8 Hz, 1H, H3), 3.27 (s, 3H, CH_3_), 3.08 (d, ^2^J = 11.8 Hz, 1H, H3); ^13^C NMR (100.62 MHz, DMSO-d_6_) δ 165.4 (C2), 161.3 (C4), 147.8 (dd, ^1^JF = 247.9, ^2^JF = 13.8 Hz, C8), 146.1 (dd, ^1^JF = 244.2, ^2^JF = 13.7 Hz, C7), 138.1 (dd, ^3^JF = 8.7, ^4^JF = 2.3 Hz, C5a), 136.3 (C1′), 131.6 (m, C9a, C4′), 128.8 (C3′–C5′), 127.7 (C2′–C6′), 114.3 (d, ^2^JF = 18.3 Hz, C6), 111.3 (d, ^2^JF = 20.4 Hz, C9), 39.1 (C3), 34.7 (CH_3_); ^15^N NMR (40.54 MHz, DMSO-d_6_) δ –251.6 (N1), –72.4 (N5); ^19^F NMR (376.50 MHz, DMSO-d_6_) δ–141.8 (ddd, ^3^JFF = 24.2, ^4^JH9 = 12.5, ^3^JH6 = 8.7 Hz, F7), –139.9 (ddd, ^3^JFF = 24.2, ^4^JH6 = 11.7, 3JH9 = 8.0 Hz, F8); Anal. Calcd for C_16_H_12_F_2_N_2_O (%): C, 67.13; H, 4.23; N, 9.79. Found: C, 66.72; H, 4.32; N, 9.69.

#### 2.3.3. 8-Fluoro-4-phenyl-1H-1,5-benzodiazepin-2(3H)-one (**11**) and 7-Fluoro-4-phenyl-H-1,5-benzodiazepin-2(3H)-one (**13**)

4-Fluorobenzene-1,2-diamine (0.50 g, 3.96 mmol) and ethyl benzoylacetate (0.71 mL, 4.08 mmol) were heated at 120 °C in anhydrous xylene (7 mL) for 6 h. The mixture was cooled, and then a solid precipitated. This residue was washed with ethyl ether to yield a mixture of the compounds **11** and **13** (0.54 g, 53%) as a pale yellow solid. The mixture was chromatographed (hexane/ethyl acetate, 10:1) to yield 11 and 13, i.e., 30% and 70%, respectively.

Compound **11**: m.p. 212.5 °C (EtOH); ^1^H NMR (400.13 MHz, DMSO-d_6_) δ 10.65 (s, 1H, H1), 8.05 (dd, ^3^J = 7.8, ^4^J = 1.8 Hz, 2H, H2′–H6′), 7.54 (m, 3H, H3′–H5′, H4′), 7.44 (dd, ^3^J = 8.9, ^4^JF = 6.2 Hz, 1H, H6), 7.10 (ddd, ^3^J = 8.9, ^3^JF = 8.1, 4J = 2.9 Hz, 1H, H7), 6.98 (dd, ^3^JF = 10.1, 4J = 2.9 Hz, 1H, H9), 3.54 (s, 2H, H3); ^13^C NMR (100.62 MHz, DMSO-d_6_) δ 166.0 (C2), 159.5 (d, ^1^JF = 243.4 Hz, C8), 158.1 (C4), 137.1 (C1′), 136.2 (d, ^4^JF = 2.2 Hz, C5a), 131.3 (d, ^3^JF = 11.1 Hz, C9a), 131.0 (C4′), 129.8 (d, ^3^JF = 9.9 Hz, C6), 128.7 (C3′–C5′), 127.5 (C2′–C6′), 111.6 (d, ^2^JF = 22.6 Hz, C7),107.7 (d, ^2^JF = 24.9 Hz, C9), 39.7 (C3); ^15^N NMR (40.54 MHz, DMSO-d_6_) δ–240.8 (N1), n.o. (N5); ^19^F NMR (376.50 MHz, DMSO-d_6_) δ–116.7 (ddd, ^3^JH9 = 10.1, ^3^JH7 =8.1, ^4^JH6 = 6.2 Hz, F8); Anal. Calcd for C_15_H_11_FN_2_O: C, 70.86; H, 4.36; N, 11.02. Found: C, 70.70; H, 4.47; N, 10.77.

Compound **13**: m.p. 205.7 °C (EtOH); ^1^H NMR (400.13 MHz, DMSO-d_6_) δ 10.56 (s, 1H, H1), 8.06 (dd, ^3^J = 8.0, ^4^J = 1.6 Hz, 2H, H2′–H6′), 7.55 (m, 3H, H3′–H5′, H4′), 7.18 (m, 3H, H6, H8, H9), 3.52 (s, 2H, H3); ^13^C NMR (100.62 MHz, DMSO-d_6_) δ 166.0 (C2), 159.6 (C4), 158.2 (d, ^1^JF = 241.0 Hz, C7), 140.5 (d, J = 11.0 Hz, C5a), 136.9 (C1′), 131.3 (C4′), 128.7 (C3′–C5′), 127.7 (C2′–C6′), 126.8 (C9a), 123.5 (d, ^3^JF = 9.1 Hz, C9), 113.5 (d, ^2^JF = 22.9 Hz, C8), 112.9 (d, ^2^JF = 23.2 Hz, C6), 39.9 (C3); ^15^N NMR (40.54 MHz, DMSO-d_6_) δ –243.1 (N1), n.o. (N5); ^19^F NMR (376.50 MHz, DMSO-d_6_) δ–118.8 (ddd, ^3^JH6 = 10.0, ^3^JH8 =8.1, ^4^JH9 = 5.7 Hz, F7); Anal. Calcd for C_15_H_11_FN_2_O: C, 70.86; H, 4.36; N, 11.02. Found: C, 70.63; H, 4.45; N, 10.76.

#### 2.3.4. 8-Fluoro-1-methyl-4-phenyl-1H-1,5-benzodiazepin-2(3H)-one (**12**)

A solution of 8-fluoro-4-phenyl-1H-1,5-benzodiazepin-2(3H)-one (0.39 g, 1.54 mmol) in DMF (2 mL) was heated at 110 °C in the presence of iodomethane (0.10 mL, 1.69 mmol), K_2_CO_3_ (0.25 g, 1.84 mmol) and a catalytic quantity of KI for 3 h 30 min. The mixture was cooled, treated with cold water, and extracted with ethyl acetate. The ethyl acetate was evaporated, and the crude extract was purified by column chromatography (hexane/ethyl acetate, 10:1) to yield compound **12** (0.18 g, 44%). m.p. oil. ^1^H NMR (400.13 MHz, DMSO-d_6_) δ 8.07 (dd, ^3^J = 8.0, 4J = 1.7 Hz, 2H, H2′–H6′), 7.54 (m, 3H, H3′–H5′, H4′), 7.42 (m, 2H, H6, H9), 7.17 (ddd, ^3^J = 9.0, ^3^JF =7.9, ^4^J = 2.8 Hz, 1H, H7), 4.14 (bs, 1H, H3), 3.29 (s, 3H, CH_3_), 3.04 (bs, 1H, H3); ^13^C NMR (100.62 MHz, DMSO-d_6_) δ 165.4 (C2), 160.0 (C4), 159.6 (d, ^1^JF = 242.6 Hz, C8), 137.7 (d, ^4^JF = 2.3 Hz, C5a), 136.6 (C1′), 135.9 (d, ^3^JF = 10.2 Hz, C9a), 131.2 (C4’), 128.7 (C3’-C5’), 128.6 (d, ^3^JF = 9.5 Hz, C6), 127.5 (C2′–C6′), 112.4 (d, ^2^JF = 22.7 Hz, C7), 108.8 (d, ^2^JF = 25.6 Hz, C9), 39.0 (C3), 34.6 (CH_3_); ^15^N NMR (40.54 MHz, DMSO-d_6_) δ –251.0 (N1), –70.5 (N5); ^19^F NMR (376.50 MHz, DMSO-d_6_) δ–115.4 (m, F8); Anal. Calcd for C_16_H_13_FN_2_O (%): C, 71.63; H, 4.88; N, 10.44. Found: C, 70.78; H, 5.21; N, 9.78.

#### 2.3.5. 7-Fluoro-1-methyl-4-phenyl-1H-1,5-benzodiazepin-2(3H)-one (**14**)

A solution of 7-fluoro-4-phenyl-1H-1,5-benzodiazepin-2(3H)-one (0.50 g, 1.97 mmol) in DMF (2 mL) was heated at 110 °C in the presence of iodomethane (0.14 mL, 2.16 mmol), K_2_CO_3_ (0.33 g, 2.36 mmol) and a catalytic quantity of KI for 2 h 30 min. The mixture was cooled, treated with cold water, and extracted with ethyl acetate. The ethyl acetate was evaporated, and the crude extract was purified by column chromatography (hexane/ethyl acetate, 10:1) to yield compound **14** (0.33 g, 63%). m.p. 107.7 °C (EtOH). ^1^H NMR (400.13 MHz, DMSO-d6) δ 8.08 (dd, ^3^J = 8.2, ^4^J = 1.6 Hz, 2H, H2′–H6′), 7.56 (m, 4H, H3′–H5′, H4′, H9), 7.21 (m, 2H, H6, H8), 4.15 (d, ^2^J = 12.2 Hz, 1H, H3), 3.28 (s, 3H, CH_3_), 3.04 (d, ^2^J = 12.2 Hz, 1H, H3); ^13^C NMR (100.62 MHz, DMSO-d_6_) δ 165.4 (C2), 161.5 (C4), 158.5 (d, ^1^JF = 242.4 Hz, C7), 142.2 (d, ^3^JF = 11.1 Hz, C5a), 136.4 (C1’), 131.6 (d, ^4^JF = 2.3 Hz C9a), 131.5 (C4′), 128.8 (C2′–C6′), 127.7 (C3′–C5′), 124.2 (d, ^3^JF = 9.7 Hz, C9), 113.4 (d, ^2^JF = 23.4 Hz, C8), 112.1 (d, ^2^JF = 23.0 Hz, C6), 39.2 (C3), 34.7 (CH_3_); ^15^N NMR (40.54 MHz, DMSO-d_6_) δ–240.8 (N1), n.o. (N5); ^19^F NMR (376.50 MHz, DMSO-d_6_) δ–118.8 (ddd, ^3^JH6 = 10.0, ^3^JH8 =8.1, ^4^JH9 = 5.7 Hz, 1F, F7); Anal. Calcd for C_16_H_13_FN_2_O (%): C, 71.63; H, 4.88; N, 10.44. Found: C, 71.28; H, 4.84; N, 10.34.

#### 2.3.6. 7,9-Difluoro-4-phenyl-1H-1,5-benzodiazepin-2(3H)-one (**15**) and 6,8-Difluoro-4-phenyl-1H-1,5-benzodiazepin-2(3H)-one (**17**)

3,5-Difluorobenzene-1,2-diamine (0.50 g, 3.47 mmol) and ethyl benzoylacetate (0.62 mL, 3.57 mmol) were heated at 120 °C in anhydrous xylene (5 mL) for 4 h. The mixture was cooled, and then a solid precipitated. This residue was washed with ethyl ether to yield a mixture of the compounds **15** and **17** (0.50 g, 55%) as a pale yellow solid. The mixture was chromatographed (hexane/ethyl acetate, 10:1) to yield 15 and 17, 72% and 28%, respectively.

Compound **15**: m.p. 213.4 °C (EtOH); ^1^H NMR (400.13 MHz, DMSO-d_6_) δ 10.45 (s, 1H, H1), 8.08 (dd, ^3^J = 8.0, ^4^J = 1.8 Hz, 2H, H2′–H6′), 7.56 (m, 3H, H3′–H5′, H4′), 7.29 (ddd, ^3^JF9 = 10.6, ^3^JF7 = 8.7, 4J = 2.9 Hz, 1H, H8), 7.10 (ddd, ^3^JF7 = 9.9, ^4^J = 2.9, ^5^JF7 = 1.8 Hz, 1H, H6), 3.61 (s, 2H, H3); ^13^C NMR (100.62 MHz, DMSO-d_6_) δ 165.8 (C2), 161.6 (C4), 157.5 (dd, ^1^JF = 242.0, ^2^JF = 14.4 Hz, C7), 153.3 (dd, ^1^JF = 248.3, ^3^JF = 14.9 Hz, C9), 142.2 (d, ^3^JF = 14.2 Hz, C5a), 136.5 (C1′), 131.6 (C4′), 128.8 (C3′–C5′), 127.8 (C2′–C6′), 116.1 (dd, ^2J^F = 13.7, ^4^JF = 1.9 Hz, C9a), 108.5 (dd, ^2^JF = 22.9, ^4^JF = 3.4 Hz, C6), 101.3 (dd, ^2^JF = 27.7, ^2^JF = 24.5 Hz, C8), 40.1 (C3); ^15^N NMR (40.54 MHz, DMSO-d_6_) δ–253.6 (N1), n.o. (N5); ^19^F NMR (376.50 MHz, DMSO-d6) δ–119.9 (ddd, ^3^JH8 = 10.3, ^4^JFF = 4.6, ^5^JH6 = 1.5 Hz, 1F, F9),–116.4 (td, ^3^JH8 δ 3JH6 δ 9.3, ^4^JFF = 4.9 Hz, 1F, F7); Anal. Calcd for C_15_H_10_F_2_N_2_O: C, 66.18; H, 3.70; N, 10.29. Found: C, 65.88; H, 3.66; N, 10.10.

Compound 17: m.p. 221.5 °C (EtOH); ^1^H NMR (400.13 MHz, DMSO-d_6_) δ 10.84 (s, 1H, H1), 8.06 (dd, ^3^J = 8.0, 4J = 1.7 Hz, 2H, H2′–H6′), 7.55 (m, 3H, H3′–H5′, H4′), 7.20 (ddd, 3JF = 10.6, ^3^JF =8.9, ^4^J = 2.8 Hz, 1H, H7), 6.85 (ddd, ^3^JF = 10.1, ^4^J = 2.8, ^5^JF = 1.9 Hz, 1H, H9), 3.63 (s, 2H, H3); ^13^C NMR (100.62 MHz, DMSO-d_6_) δ 165.8 (C2), 159.2 (C4), 159.1 (dd, ^1^JF = 243.6, ^3^JF = 15.0 Hz, C8), 157.2 (dd, ^1^JF = 249.8, ^3^JF = 15.0 Hz, C6), 136.7 (C1′), 132.5 (dd, ^3^JF = 13.3, ^3^JF = 12.7 Hz, C9a), 131.4 (C4′), 128.8 (C3′–C5′), 127.7 (C2′–C6′), 125.8 (dd, ^2^JF = 12.6, ^4^JF = 3.6 Hz, C5a), 103.4 (dd, ^2^JF = 24.9, ^4^JF = 3.5 Hz, C9), 99.8 (dd, ^2^JF = 26.9, ^2^JF = 25.2 Hz, C7), 40.2 (C3); ^15^N NMR (40.54 MHz, DMSO-d_6_) δ–240.1 (N1), n.o. (N5); ^19^F NMR (376.50 MHz, DMSO-d_6_) δ–115.4 (m, 1F, F6),–113.3 (td, ^3^JH7 δ ^3^JH9 δ 9.3, ^4^JFF = 6.5 Hz, 1F, F8); Anal. Calcd for C_15_H_10_F_2_N_2_O: C, 66.18; H, 3.70; N, 10.29. Found: C, 66.33; H, 3.84; N, 10.00.

#### 2.3.7. 7,9-Difluoro-1-methyl-4-phenyl-1H-1,5-benzodiazepin-2(3H)-one (**16**)

A solution of 7,9-difluoro-4-phenyl-1H-1,5-benzodiazepin-2(3H)-one (0.26 g, 0.98 mmol) in DMF (1 mL) was heated at 110 °C in the presence of iodomethane (0.07 mL, 1.08 mmol), K_2_CO_3_ (0.16 g, 1.18 mmol), and a catalytic quantity of KI for 2 h. The mixture was cooled, treated with cold water, and extracted with ethyl acetate. The ethyl acetate was evaporated, and the crude extract was purified by column chromatography (hexane/ethyl acetate, 10:1) to yield compound 16 (0.21 g, 75%). m.p. 60.2 °C (EtOH). ^1^H NMR (400.13 MHz, DMSO-d_6_) δ 8.09 (dd, ^3^J = 8.2, ^4^J = 1.5 Hz, 2H, H2′–H6′), 7.56 (m, 3H, H3′–H5′, H4′), 7.33 (ddd, ^3^JF = 11.9, ^3^JF = 8.7, 3J = 3.0 Hz, 1H, H8), 7.10 (ddd, ^3^JF = 9.7, ^3^J = 2.9, ^5^JF = 1.9 Hz, 1H, H6), 4.15 (d, ^3^J = 12.5 Hz, 1H, H3), 3.22 (d, ^3^J = 12.5 Hz, 1H, H3), 3.15 (d, ^5^JF = 4.3 Hz, 3H, CH_3_); ^13^C NMR (100.62 MHz, DMSO-d_6_) δ 165.6 (C2), 163.6 (C4), 158.6 (dd, ^1^JF = 243.7, ^3^JF = 15.1 Hz, C7), 155.2 (dd, ^1^JF = 250.6, ^3^JF = 14.4 Hz, C9), 144.1 (dd, ^3^JF = ^3^JF = 13.0 Hz, C5a), 136.2 (C1′), 131.8 (C4′), 128.8 (C3′–C5′), 127.9 (C2′–C6′), 120.6 (dd, ^2^JF = 10.9, ^4^JF = 4.0 Hz, C9a), 107.8 (dd, ^2^JF = 23.3, ^4^JF = 3.0 Hz, C6), 102.0 (dd, ^2^JF = 27.1, ^2^JF = 25.9 Hz, C8), 39.1 (C3), 35.7 (d, ^4^JF = 7.8 Hz, CH_3_); ^15^N NMR (40.54 MHz, DMSO-d_6_) δ–263.0 (N1), –73.3 (N5); ^19^F NMR (376.50 MHz, DMSO-d_6_) δ–113.7 (td, ^3^JH6 δ ^3^JH8 δ 9.2, ^4^JFF = 6.8 Hz, 1F, F7),–113.2 (m, 1F, F9); Anal. Calcd for C_16_H_12_F_2_N_2_O (%): C, 67.13; H, 4.23; N, 9.79. Found: C, 66.11; H, 4.31; N, 9.72.

#### 2.3.8. 4-Phenyl-1H-1,5-benzodiazepin-2(3H)-one (**18**)

Benzene-1,2-diamine (0.50 g, 4.62 mmol) and ethyl benzoylacetate (0.82 mL, 4.76 mmol) were heated at 120 °C in anhydrous xylene (5 mL) for 24 h. The mixture was cooled, and then a solid precipitated. This residue was washed with ethyl ether to yield compound **18** (0.90 g, 82%) as a pale yellow solid: m.p. 208.2 °C (EtOH). ^1^H NMR (400.13 MHz, DMSO-d_6_) δ 10.55 (s, 1H, H1), 8.06 (dd, ^3^J = 7.7, ^4^J = 2.0 Hz, 2H, H2′–H6′), 7.53 (m, 3H, H3′–H5′, H4′), 7.40 (dd, ^3^J = 7.5, ^4^J = 1.9 Hz, 1H, H6), 7.23 (m, 3H, H7, H8, H9), 3.49 (s, 2H, H3); ^13^C NMR (100.62 MHz, DMSO-d_6_) δ 166.2 (C2), 158.3 (C4), 139.4 (C5a), 137.2 (C1**’**), 130.1 (C9a), 131.0 (C4′), 128.7 (C3′–C5′), 127.7 (C6), 127.5 (C2′–C6′), 126.1 (C8), 124.1 (C7), 121.9 (C9), 39.8 (C3); ^15^N NMR (40.54 MHz, DMSO-d_6_) δ–242.0 (N1), n.o (N5).

#### 2.3.9. 1-Methyl-4-phenyl-1H-1,5-benzodiazepin-2(3H)-one (**19**)

A solution of 4-phenyl- 1H-1,5-benzodiazepin-2(3H)-one (0.50 g, 2.12 mmol) in DMF (2 mL) was heated at 110 °C in the presence of iodomethane (0.14 mL, 2.33 mmol), K_2_CO_3_ (0.35 g, 2.54 mmol), and a catalytic quantity of KI for 3 h. The mixture was cooled, treated with cold water, and extracted with ethyl acetate. The ethyl acetate was evaporated, and the crude extract was purified by column chromatography (hexane/ethyl acetate, 10:1) to yield compound 19 (0.35 g, 65%). m.p. oil. ^1^H NMR (400.13 MHz, DMSO-d_6_) δ 8.08 (dd, ^3^J = 7.9, ^4^J = 1.8 Hz, 2H, H2′–H6′), 7.53 (m, 4H, H3′–H5′, H4′, H9), 7.33 (m, 3H, H6, H7, H8), 4.13 (d, ^2^J = 11.3 Hz, 1H, H3), 3.30 (s, 3H, CH3), 2.98 (d, ^2^J = 11.3 Hz, 1H, H3); ^13^C NMR (100.62 MHz, DMSO-d_6_) δ 165.4 (C2), 160.2 (C4), 140.9 (C5a), 136.7 (C1′), 134.8 (C9a), 131.2 (C4′), 128.7 (C3′–C5′), 127.5 (C2′–C6′), 126.7 (C6), 126.2 (C8), 124.9 (C7), 122.2 (C9), 39.0 (C3), 34.6 (CH3); ^15^N NMR (40.54 MHz, DMSO-d_6_) δ–251.9 (N1),–68.6 (N5).

#### 2.3.10. 4-(3,4,5-Trimethoxyphenyl)-1H-1,5-benzodiazepin-2(3H)-one (**20**)

Benzene-1,2-diamine (0.22 g, 2 mmol) and ethyl 3-oxo-3-(3,4,5-trimethoxyphenyl)-propanoate (0.56 g, 2 mmol) were heated at 120 °C in anhydrous xylene (8 mL) for 7 h. The mixture was cooled, and then a solid precipitated. This residue was washed with ethyl ether to yield compound 20 (0.50 g, 77%) as a white solid: m.p. 215.4 °C (EtOH). ^1^H NMR (400.13 MHz, DMSO-d_6_) δ 10.53 (s, 1H, H1), 7.39 (ddd, ^3^J = 7.2, ^4^J = 2.1, ^5^J = 0.8 Hz, 1H, H6), 7.34 (s, 2H, H2′–H6′), (7.17-7.27 (mc, 3H, H7, H8,H9), 3.86 (s, 6H, OMe3′–5′), 3.73 (s, 3H, OMe-4′), 3.50 (bs, 2H, H3);^13^C NMR (100.62 MHz, DMSO-d_6_) δ 166.2 (C2), 157.9 (C4), 152.8 (C3′–5′), 140.3 (C4′), 139.4 (C9a), 132.6 (C1′), 130.1 (C5a), 127.6 (C6), 126.0 (C7), 124.1 (C8), 121.9 (C9), 105.3 (C2′–6′), 60.2 (OMe 4′), 56.1 (OMe3′–5′), 39.6 (C3); ^15^N NMR (40.54 MHz, DMSO-d_6_) δ–240.1 (N1), n.o. (N5). Anal. Calcd. for C_18_H_18_N_2_O_4_: C, 66.25; H, 5.56; N, 8.58. Found: C, 65.86; H, 5.45; N, 8.48.

#### 2.3.11. 7,8-Dimethyl-4-(3,4,5-trimethoxyphenyl)-1H-1,5-benzodiazepin-2(3H)-one (**21**)

4,5-Dimethylbenzene-1,2-diamine (0.38 g, 2.82 mmol) and ethyl 3-oxo-3- (3,4,5-trimethoxyphenyl) propanoate (0.80 g, 2.82 mmol) were heated at 120 °C in anhydrous xylene (8 mL) for 6 h. The mixture was cooled, and then a solid precipitated. This residue was washed with ethyl ether to yield compound **21** (0.83 g, 82%) as white solid: m.p. 208.5 °C (EtOH). ^1^H NMR (400.13 MHz, DMSO-d_6_) δ 10.36 (s, 1H, H1), 7.32 (s, 2H, H2′–H6′), 7.18 (s, 1H, H6), 6.93 (s, 1H, H9), 3.86 (s, 6H, OMe3′–5′), 3.73 (s, 3H, OMe4′), 3.45 (bs, 2H, H3), 2.24 (s, 3H, Me8), 2.23 (s, 3H, Me7); ^13^C NMR (100.62 MHz, DMSO-d_6_) δ 165.9 (C2), 156.8 (C4), 152.8 (C3′–5′),140.1 (C1′), 137.4 (C9a), 134.5 (C5a), 132.8 (C4′), 128.1 (C6), 127.8 (C7), 124.1 (C8), 122.3 (C9), 105.2 (C2′–6′), 60.2 (OMe-4′), 56.0 (OMe-3′–5′), 39.6 (C3), 19.0 (Me8), 18.7 (Me7); ^15^N NMR (40.54 MHz, DMSO-d_6_) δ –240.6 (N1),–69.4 (N5). Anal. Calcd. for C_20_H_22_N_2_O_4_: C, 67.78; H, 6.26; N, 7.90. Found: C, 67.59; H, 6.13; N, 7.90.

#### 2.3.12. 7,8-Difluoro-4-(3,4,5-trimethoxyphenyl)-1H-1,5-benzodiazepin-2(3H)-one (**22**)

4,5-Difluorobenzene-1,2-diamine (0.20 g, 1.39 mmol) and ethyl 3-oxo-3-(3,4,5-trimethoxyphenyl) propanoate (0.39 g, 1.39 mmol) were heated at 120 °C in anhydrous xylene (5 mL) for 6 h. The mixture was cooled, and then a solid precipitated. This residue was washed with ethyl ether to yield compound **22** (0.39 g, 85%) as a white solid: m.p. 241.8 °C (EtOH). ^1^H NMR (400.13 MHz, DMSO-d_6_) δ 10.60 (bs, 1H, H1), 7.46 (dd, ^4^JF = 11.7, ^3^JF = 8.5 Hz 1H, H6), 7.33 (s, 2H, H3′–5′), 7.17 (dd, ^4^JF = 11.7, ^3^JF = 8.1 Hz 1H, H9), 3.86 (s, 6H, OMe3′–5′), 3.73 (s, 3H, OMe4′), 3.57 (bs, 2H, H3);^13^C NMR (100.62 MHz, DMSO-d_6_) δ 166.0 (C2), 159.1 (C4), 152.9 (C3′–5′), 146.9 (d, ^1^JF = 245.4, ^2^JF = 14.1 Hz, C7), 145.7 (d, ^1^JF = 242.9, ^2^JF = 13.8 Hz, C8), 140.6 (C4′), 136.4 (C9a), 127.0 (C5a), 115.4 (d, ^2^JF = 18.0 Hz, C6), 109.81 (d, ^2^JF = 20.2 Hz, C9). 105.5 (C2′–6′), 60.2 (OMe-4′), 56.1 (OMe-3′–5′), 39.7 (C3), ^19^F NMR (376.5 MHz, DMSO-d_6_) δ 140.41 (ddd, JFF = 24.1, ^4^JH6 = 11.8, ^3^JH9 = 8.5 Hz, F8),–142.94 (ddd, JFF = 24.1, ^4^JH9 = 11.7, ^3^JH6 = 8.0 Hz, F7).^15^N NMR (40.54 MHz, DMSO-d_6_) δ–240.7 (N1), n.o. (N5). Anal. Calcd. for C_18_H_16_F_2_N_2_O_4_: C, 59.67; H, 4.45; N, 7.73. Found: C, 59.44; H, 4.40; N, 7.73.

#### 2.3.13. 7,8-Dichloro-4-(3,4,5-trimethoxyphenyl)-1H-1,5-benzodiazepin-2(3H)-one (**23**)

4,5-Dichlorobenzene-1,2-diamine (0.31 g, 1.77 mmol) and ethyl 3-oxo-3-(3,4,5-trimethoxyphenyl) propanoate (0.50 g, 1.77 mmol) were heated at 120 °C in anhydrous xylene (8 mL) for 6 h. The mixture was cooled, and then a solid precipitated. This residue was washed with ethyl ether to yield compound **23** (0.56 g, 80%) as a pale brown solid: m.p. 246.0 °C (EtOH). 1H NMR (400.13 MHz, DMSO-d6) δ 10.70 (bs, 1H, H1), 7.64 (s, 1H, H6), 7.39 (s, 1H, H9), 7.34 (s, 2H, H2′–H6′), 3.86 (s, 6H, OMe3′–5′), 3.73 (s, 3H, OMe4′), 3.61 (bs, 2H, H3);^13^C NMR (100.62 MHz, DMSO-d_6_) δ 166.0 (C2), 159.9 (C4), 152.8 (C3′–5′),140.7 (C4′), 139.2 (C9a), 132.0 (C1′), 130.2 (C5a), 128.7 (C6), 127.5 (C7), 125.7 (C8), 122.9 (C9), 105.6 (C2′–6′), 60.2 (OMe-4′), 56.1 (OMe-3′–5′), 39.6 (C3); ^15^N NMR (40.54 MHz, DMSO-d_6_) δ –237.5 (N1), n.o. (N5). Anal. Calcd. for C_18_H_16_Cl_2_N_2_O_4_: C, 54.70; H, 4.08; N, 7.09. Found: C, 54.33; H, 3.98; N, 7.15.

### 2.4. Radical Scavenger Capacity Determination

#### 2.4.1. 2,2′-Azino-bis (3-ethylbenzothiazoline-6-sulfonic Acid) (ABTS) Assay

ABTS^+^ radical was produced by reacting 2,2′-azino-bis (3-ethylbenzthiazoline-6-sulphonic acid) (ABTS) 7 mM with K_2_S_2_O_8_ 2.45 mM; both reactants dissolved in water at a volume ratio of 1:1. The mixture was stored in the dark at room temperature for 16 h. The ABTS^+^ solution was diluted to yield an absorbance of 0.750 ± 0.025 at 734 nm in EtOH. All the compounds tested were dissolved in EtOH. Then, 150 µL of each compound solution was added to the wells of a 96-well culture plate, followed by the addition of 50 µL of the ABTS^+^ radical solution. The final compound concentrations tested were 0, 5, 10, 20, 50, 100, 150, and 200 µM. The absorbance change at 734 nm was recorded after 30-min incubation in the dark, and the percentage of radical scavenging was calculated for each concentration relative to a blank containing no scavenger. The degree of decolorization was calculated as the percentage reduction of absorbance. The ABTS^+^ radical scavenging activity was calculated as follows:

Percentage ABTS^+^ radical scavenging activity = {1 − [As/Ac]} × 100 (1)

where A is the absorbance of the ABTS^+^ radical solution containing samples, and Ac is the absorbance of the control solution without antioxidant. The percentages of ABTS^+^ radical reduction were plotted against the compound concentration. Trolox was used as a reference antioxidant. All the assays were carried out three times in quadruplicate for each compound.

#### 2.4.2. Ferric Reducing Antioxidant Power (FRAP) Assay

The assay was carried out according to the method of Benzie et al. [79], with slight modifications, in a 96-well culture microplate. FRAP reagent was prepared by mixing 10 mL of 300 mM acetate buffer with 1 mL of 10 mM 2,4,6-tripyridyl-*S*-triazine (TPTZ) in HCl 40 mM and 1mL of FeCl_3_·6H_2_O 20 mM. Firstly, 190 µL of the FRAP reagent was added to all wells of a 96-well culture plate, followed by the addition of 10 µL of the test compounds dissolved in Ethanol to reach the final concentrations assayed (0, 5, 10, 20, 50, 100, 150, 200 µM). After 30-min incubation in the dark, the absorbance was read at 593 nm. Trolox was used as assay control. Three different assays were carried out in quadruplicate for each compound.

#### 2.4.3. 2,2-Diphenyl-1-picrylhydrazyl (DPPH) Assay

The assay was performed in a 96-well culture plate. A 0.2 mM solution of 2,2-diphenyl-1-picryl-hydrazyl-hydrate (DPPH) in ethanol was prepared. All the compounds tested were dissolved in EtOH. Firstly, 150 µL of each compound solution was added to the wells of the 96-well culture plate, followed by the addition of 50 µL of the DPPH solution. The final compound concentrations tested were 0, 5, 10, 20, 50, 100, 150, 200 µM. The change in absorbance at 517 nm was measured after 30-min incubation in the dark. The free radical scavenging activity was calculated as inhibition using Equation (2):

Percentage DPPH radical scavenging activity = {1 − [As/Ac]} × 100(2)

where As is the absorbance of the DPPH solution containing the samples and Ac is the absorbance of the control solution without antioxidant but with DPPH. The percentages of DPPH reduction were plotted against the compound concentration. The experiment was also conducted using Trolox as a reference antioxidant. Assays were performed three times in quadruplicate for each compound.

### 2.5. Cell Culture

Human neuroblastoma SH-SY5Y cells were obtained from Prof. Ricardo Martinez Murillo (Neurovascular Research Group, Department of Translational Neurobiology, Cajal Institute. Madrid. Spain) and cultured in DMEM-F12 (1:1) medium supplemented with 10% heat-inactivated fetal bovine serum (FBS), 1% 100 mg/mL penicillin, and 100 mg/mL streptomycin. Human hepatoma HepG2 cells were obtained from ATCC and cultured in RPMI 1640 medium supplemented with 10% heat-inactivated fetal bovine serum (FBS), 1% 100 mg/mL penicillin, and 100 mg/mL streptomycin. All cell lines were incubated at 37 °C in a humidified atmosphere incubator at 5% CO_2_. The culture medium was changed every other day, and the cells were sub-cultured after they reached 75–80% confluence.

For differentiation experiments, SH-SY5Y cells were plated in 96-well culture plates (1.5 × 10^3^ cells per well) for neuroprotection experiments, and in 24-well culture plates (6 × 10^3^ cells per well) with 12 mm poly-lysine treated coverslips for fluorescence microscopy studies.

After 24 of seeding, SH-SY5Y cells were exposed to 10 µM all-trans retinoic acid (RA) in DMEM:F12 (1:1) with 1% FBS for eight days before compound treatment. The medium was replaced every 48 h on days 2, 4, 6, and 8. On day eight, compounds were administered in the same media for protection experiments.

### 2.6. Cytotoxicity Activity In Vitro in SH-SY5Y and HepG2 Cell Lines

SH-SY5Y and HepG2 cells were seeded into 96-well culture plates at a density of 5 × 10^3^ cells/well and grown in an incubator for 24 h. All compounds tested were dissolved and eventually further diluted in dimethylsulfoxide (DMSO). After overnight incubation, the cells were treated with different compound concentrations (5, 10, 20, 50, 100, 150, 200 µM) in a final volume of 200 µL with four replicates each. The concentration of DMSO did not exceed 0.2%, which is considered non-toxic to cells [80]. Cell proliferation or viability was measured using the 3-(4,5-dimethylthiazol-2-yl)-2,5-diphenyl tetrazolium bromide (MTT) assay. The MTT assay was optimized taking into account the number of cells plated per well, to work in the linear part of the cell density vs. MTT absorbance calibration curve, which allows achieving a maximum absorbance in the range of 0.8–1.0 in each well at the end of the experiment. In addition to that, the number of cells seeded for each experiment was optimized in agreement with the time scale of the experiment.

After cell treatment, 20 µL of MTT (5 mg/mL) was added to each well, and the plate was incubated at 37 °C in the dark for 2 h. Supernatants were removed, and the formazan crystals were solubilized in DMSO (200 µL/well), under 10 min shaking at room temperature. The reduction of MTT was quantified by absorbance at 570 nm in a Varioskan microplate reader (Thermo Fisher Scientific, Vantaa, Finland). Effects of the test compounds on cell viability were calculated using cells treated with a vehicle as a control. For IC_50_ and LD_50_ calculations, the data were subjected to linear regression analysis, and the regression lines were plotted for the best fit. The **IC**_50_ (inhibition of cell viability) concentrations were calculated using the respective dose–response curves.

### 2.7. SH-SY5Y Cell Neuroprotection Assay

Compound neuroprotection under H_2_O_2_-, 6-OHD- or MPP^+^-induced oxidative stress was firstly assessed by MTT assay. Briefly, SH-SY5Y was seeded into 96-well culture plates at a density of 3 × 103 cells/well and grown in the incubator for 24 h. Cells were pretreated for 24 h with different compound concentrations (5, 10, 20, 30, 40, 50 µM). After the treatment period, cells were exposed to 250 µM H_2_O_2_ for 24 h or to 250 µM 6-OHD or 700 µM MPP^+^ for 48 h. Cell viability was measured with MTT following the same procedure described above. All the absorbances were subtracted from that of the background control (medium + MTT without cells). Cell viability was expressed as the percentage of the control value (cells without treatment with a stressor).

To confirm the protection results obtained from the MTT method, the most interesting derivative **18** was tested by the resazurin and Calcein-AM assays.

The resazurin method is used to measure cellular metabolic reduction as an additional cell viability assay [81]. Resazurin dye has been broadly used as an indicator of cell viability in several types of assays. Mitochondrial enzymes, as carriers of diaphorase activities, are probably responsible for the transference of electrons from NADPH to resazurin, which is reduced to resorufin. The level of reduction was quantified as a measure of cell metabolic activity [81,82]. In this assay, 10 μL resazurin (4 mg/mL) was added to each well. After 4 h of incubation at 37 °C, 5% CO_2_, fluorescence was measured in a Varioskan microplate reader (Thermo Fisher Scientific, Vantaa, Finland) with λex = 544 and λem = 612 nm. All the fluorescence values were subtracted from that of the background control (medium without cells).

Calcein-AM is a fluorescence-based cell viability assay. Calcein-acetoxymethyl (AM) is a non-fluorescent compound that enters the cells where intracellular esterases hydrolyze the acetoxymethyl group [83,84]. The fluorescent calcein is retained in the living cells, and it can be detected at λex = 495 and λem = 550 nm. After treatment, all the samples were washed with PBS and incubated with 2 μM calcein-AM for 30 min at 37 °C, 5% CO_2_, and protected from light. The fluorescence was measured in a Varioskan microplate reader (Thermo Fisher Scientific, Vantaa, Finland) with λex = 490 and λem = 520 nm filters. Cell viability was calculated from samples treated with 1% Triton X-100 for 30 min at 37 °C, 5% CO_2_.

### 2.8. Lactate Dehydrogenase (LDH) Cell V2bility Assay

SH-SY5Y 96-well culture plates at a density of 5 × 10^3^ cells/well were placed in an incubator for 24 h. Cells were pretreated for 24 h with different compound concentrations (5, 10, 20, 30, 40, 50 µM) and then exposed to 250 µM H_2_O_2_ for 24 h or to 250 µM 6-OHD or 700 µM MPP^+^ for 48 h. Then, the supernatant was used in the LDH assay, and the absorbance was measured at 490 nm in a Varioskan microplate reader after 30 min according to the manufacturer’s instructions (LDH Cytotoxicity Assay Kit, PierceTM, USA). LDH leakage was calculated as the percentage of the control group.

### 2.9. Kinetic Solubility by UV Spectrometry Assay

A modified protocol was used from that described by Hoelke et al. [85] for the determination of the kinetic solubility from DMSO compound solutions. A compound calibration curve (compound concentration vs UV absorption) was prepared for each compound with concentrations of 10, 20, 50, 100, and 200 µM. Each compound dilution was carried out by pipetting defined volumes of a 20 mM stock compound solution in DMSO into vials and adding PBS 50 mM to reach a final volume of 1 mL/well. The final amount of DMSO per well was 2%. For the lower compound concentrations, an additional volume of DMSO was added to each well to reach 2% DMSO. Then, 200 µL of each dilution was transferred to a new 96-well culture plate for the measurement of the UV-spectra. One well in each row was left without a compound as a blank, and its UV absorbance was used as an offset correction. The experiments were performed in duplicate.

In parallel, compound saturation solutions were reached and filtered from 20 mM compound stock in DMSO: 10 µL of a 20 mM stock solution was dispensed into 190 µL of PBS 10 mM. The solutions were mixed, shaken on a microplate shaker for two hours, and then 160 µL of each solution was filtered and transferred to a new well plate for UV absorption measurement. The compound solubility in PBS 50 mM was determined at 2, 6, and 24 h with solutions prepared as those for the compound calibration curve.

Measurement of the UV-spectra was performed using a Varioskan microplate reader, and spectra were taken in the range of 270–600 nm. Spectral bandwidth was set to 5 nm, and the data pint interval was 10 nm. The experiments were carried out in triplicate.

### 2.10. Estimation of Intracellular Reactive Oxygen Species (ROS)

Intracellular ROS levels were determined using dichlorofluorescein diacetate (DCFH_2_-DA) according to Wang et al. [86]. DCFH_2_-DA is cleaved by intracellular esterases and is oxidized by ROS to form the fluorescent compound DCF, which represents the ROS level. Therefore, 3 × 10^3^ SH-SY5Y cells were seeded in 96-well culture black clear bottom plates and after 24 h were treated with the test compound for an additional 24 h. Then, they were exposed to H_2_O_2_ for 4 h. After the incubation period, cells were washed twice with Hanks buffer and loaded with 20 µM DCFH_2_-DA (in FBS-free culture medium) for 40 min in dark at 37 °C in a CO_2_ incubator. At the end of the dye incubation period, they were washed twice with Hanks buffer to remove the extracellular DCFH_2_-DA. Fluorescence was measured with a Varioskan microplate reader at λex = 485 nm and λem = 535 nm wavelengths. Results are expressed as a percentage of the fluorescent increase compared with that of the control. All the experiments were performed at least three times in quadruplicate for each compound concentration.

### 2.11. Determination of Mitochondrial Superoxide (MitoSOX) Levels

Mitochondrial superoxide levels were measured with MitoSOX red (3,8-phenanthridinediamine, 5-(60-triphenylphosphoniumhexyl)-5,6-dihydro-6-phenyl) [87,88] from Molecular Probes (Thermo Fisher Scientific, 29851Willow Creek Road, Eugene, OR, USA). After SH-SY5Y cell treatment in 96-well culture black clear bottom plates, the medium was removed and cells were washed with PBS and afterward, MitoSOX red (final concentration of 5 µM) in 200 µL of PBS was added and incubated for 20 min at 37 °C. After incubation, the cells were washed with warm PBS buffer, and fluorescence was measured at 510 nm excitation/640 nm emission wavelengths in a Varioskan microplate reader (Thermo Fisher Scientific, Vantaa, Finland).

### 2.12. Determination of the Mitochondrial Membrane Potential ΔΨm (MMP)

The fluorescent dye 5,5′,6,6′-tetrachloro-1,1′,3,3′-tetraethylbenzimidazolylcarbocyanine iodide (JC-1) was used for measuring MMP [89]. A decrease in fluorescence intensity represents mitochondrial membrane depolarization. For fluorescence ratio detection under oxidative stress conditions, 96-well culture black clear bottom plates were seeded with 3 × 103 SH-SY5Y cells, and after 24-h incubation, they were treated with the test compound and left out for 24 h. Then, the cells were exposed to different H_2_O_2_ concentrations for 4 h. After the incubation period, the cells were washed twice with Hanks buffer and then stained with 5 µM JC-1 in culture media in the dark in a CO2 incubator at 37 °C for 20 min. At the end of the dye incubation period, cells were washed three times with Hanks buffer, and 200 µL of fresh buffer was added. Cells were analyzed with a Varioskan microplate reader. J-aggregate red fluorescence intensity was measured at λex = 535 nm and λem = 595 nm, while λem = 485 nm and λem = 535 nm was used for J-monomer green fluorescence. Results were expressed as the ratio of fluorescence intensity of J-aggregates to J-monomers. All the experiments were performed three times in quadruplicate for each compound concentration.

### 2.13. Determination of Apoptosis and Necrosis by Flow Cytometry

Annexin V/7-AAD-based apoptosis assay by flow cytometry was performed using a PE Annexin V Apoptosis Detection Kit I (BD Biosciences, USA) following the manufacturer’s instruction. For apoptosis and necrosis assays, 5 × 10^5^ cells/well were seeded in 6-well culture plates and treated with the compound at 10 and 20 µM for 24 h and afterward with H_2_O_2_ 1 mM for 4 h. Cells were then washed twice with chilled PBS and collected by centrifugation for 4 min at 1000× *g* at 4 °C. Then, cells were re-suspended in Ca^2+^ binding buffer; 100 μL of cell suspension (10^5^ cells) was incubated with Annexin V PE (5 μL) and 7-AAD (5 μL) for 15 min under dark conditions. After the addition of 400 μL of 1X binding buffer to each tube, samples were analyzed by flow cytometry using an Accuri C6 system (Becton Dickinson, USA), and data analyses were done using BD Accuri C6 software.

### 2.14. Evaluation of Caspase-3 Activity

Caspase-3 activity was measured using the Abcam fluorimetric kit (ab39383) according to the manufacturer’s instructions. Cells are washed with ice-cold PBS and lysed with lysis buffer. After compound and stressor treatments, SH-SY5Y cells were scraped off from p6 plates and collected in a 1.5 mL Eppendorf vial. The cell suspension was then centrifuged at 9000× *g* for 10 min at 4 °C. The supernatant was collected and stored at −80 °C for 24 h. Caspase 3 activity was measured as the ability of the cell extract to cleave the DEVD-AMC substrate and release the AMC fluorochrome. The sample’s protein concentration was determined by the Bradford method. The reaction assay was performed by the addition of 50 μL of the cell lysate into the wells of a black 96-well culture plate followed by the addition of 50 μL of 2× reaction buffer containing DTT (10 mM) and caspase-3 substrate DEVD-AFC (final concentration 50 μM). Reagents were mixed by shaking the plate for 2 min at 300 rpm and then incubated at 37 °C for 2 h. Caspase-3 activity was measured as fluorescence intensity in a Varioskan microplate reader at λex = 400 nm and λem = 505 nm wavelengths. Relative fluorescence units (RFU)/mg of protein were calculated, and the fold increase in Caspase-3 activity was compared with that of the control.

### 2.15. Determination of Lipid Peroxidation (Malondialdehyde, MDA)

For MDA studies [90,91], 2 × 10^5^ SH-SY5Y cells were seeded in 6-well culture plates. After 24 h, cells were treated with the test compound and incubated for another 24 h. After compound treatment, cells were exposed to H_2_O_2_ for 24 h. Then, the cells were washed twice with cold 50 mM PBS buffer, detached, and lysed with lysis buffer, and TBA was added to each sample tube and vortexed. The reaction mixture was incubated at 96 °C for 60 min. After incubation, samples were cooled and centrifuged 10 min at 10^4^ rpm. Samples were dispensed in 96-well culture black clear bottom plates, and fluorescence was measured with a Varioskan microplate reader at λex = 540 nm and λem = 590 nm wavelengths. Protein concentration was measured by the Bradford protein assay. The levels of MDA were expressed as nmol/mg protein. Experiments were performed four times in triplicate.

### 2.16. Determination of the Reduced GSH/Oxidized GSSG Glutathione Ratio

GSH and GSSG levels were determined by the method of Vikas et al. [92] with slight modifications. In summary, 2 × 10^5^ SH-SY5Ycells were seeded in 6-well culture plates. After 24 h, cells were treated with the test compound and incubated for another 24 h. Afterward, cells were exposed to H_2_O_2_ for 24 h. Then, cells were washed twice with cold 50 mM PBS buffer, detached, lysed with lysis buffer, and split into two samples: one for GSH analysis and the other for GSSG determination. For GSH analysis, 20 µL of ortho-phthaldehyde (OPT) 20 mg/mL and 280 µL of monobasic potassium phosphate buffer were added per 100 µL of sample replicate. Samples were vortexed and centrifuged at 4 °C at 13,000× *g* for 10 min. Afterward, 100 µL of supernatant per well was transferred to a black 96-well culture plate with a clear bottom. Fluorescence was measured in a Varioskan plate reader (λex = 360 nm; λem = 420 nm). Sample GSH concentrations were calculated from a standard calibration curve made by serial dilutions of GSH from 0 to 50 µM. For GSSG determination, to prevent oxidation of GSH to GSSG and to avoid a false overestimation of the GSSG content, 2 μL of N-ethylmaleimide (NEM) was added to 100 µL of each sample replicate and 200 μL of di-potassium hydrogen phosphate + EDTA. Samples were vortexed and incubated in the dark for 30 min at room temperature. After incubation, 20 µL of OPT and 190 µL of 0.1 N NaOH were added and incubated once again in the dark for 10 min at r.t. Samples were centrifuged at 4 °C at 13,000× *g* for 10 min. Then, 100 µL of supernatant per well was transferred to a black 96-well culture plate with a clear bottom. Fluorescence was measured in a Varioskan plate reader (λex = 360 nm; λem = 420 nm). Sample GSSG concentrations were calculated from a standard calibration curve made by serial dilutions of GSSG from 0 to 200 µM.

### 2.17. Determination of Intracellular Glutathione (GSH) Recovery

For intracellular GSH determination studies, the method of Senft et al. was used [93]. In summary, 3 × 10^3^ SH-SY5Y cells were seeded in 96-well culture black clear bottom plates and after 24 h were treated with the test compound for an additional 24 h. Then, they were exposed to H_2_O_2_ for 24 h. After the incubation period, cells were loaded with 30 µM mClB and incubated at 37 °C for 30 min in a CO_2_ incubator as described by Sebastia et al. [94]. At the end of the mClB incubation period, fluorescence was measured with a Varioskan microplate reader at λex = 405 nm and λem = 475. Results are expressed as the percentage of the fluorescence increase compared with that of the control. Experiments were performed four times in triplicate.

### 2.18. Fluorescence Microscopy Experiments

Differentiated SH-SY5Y cells grown on coverslips were washed with PBS 20 mM on day 8 and fixed with cool 4% paraformaldehyde for 10 min. Afterward, cells were washed three times with cool PBS and blocked with 0.2% Tween-20 in PBS. For immunocytochemistry visualization of neuronal processes, βIII-tubulin and MAP2 antibodies were used as neuronal markers (anti-MAP2, Sigma–Aldrich (M9942); anti- βIII-tubulin, Sino Biological (101288-T34)). DAPI was used for nuclei staining and Phalloidin-iFluor 594 Abcam (ab176757) for actin filaments. After primary antibody incubation, coverslips were washed with PBS and PBS with 0,2% Tween 20 and incubated with secondary antibodies (Goat Anti-Rabbit IgG H&L, Abcam (ab150077), and Alexa Fluor^®^ 488 AffiniPure Goat Anti-Rabbit IgG (111-545-144)), and DAPI was used for nuclei staining. After that coverslips were mounted on glass slides for fluorescence microscopy imaging. Leica LAS-X software was used for collecting images. Fiji ImageJ v1.52i was used for image processing [95].

### 2.19. Statistical Analysis

Data are expressed as the mean ± SEM of three or four experiments performed on different days and carried out in quadruplicate unless otherwise indicated. Comparisons were made between control and treated groups or the entire intragroup using one-way ANOVA or two-way ANOVA and Dunnett’s multiple comparisons test using GraphPad Prism 7.0 (GraphPad-Software, La Jolla, CA, USA) [96].

## 3. Results

### 3.1. Synthesis and Structure Characterization

**Scheme 1:**
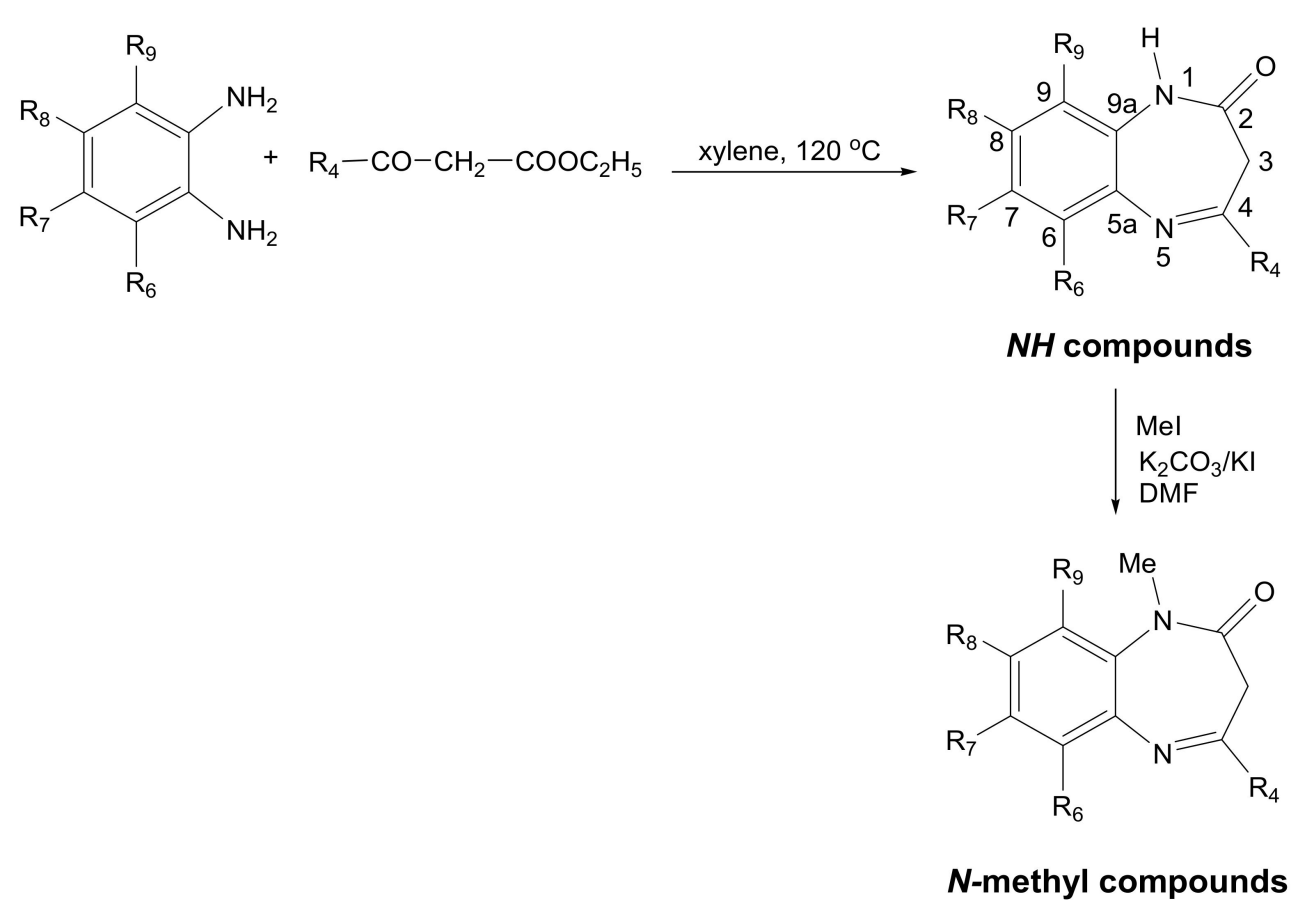
Condensation of benzene-1,2-diamines with β-oxoesters and subsequent methylation.

The most common method used to prepare the 1H-1,5-benzodiazepin-2(3H)-one core is the condensation reaction of appropriately substituted benzene-1,2-diamines with β-oxoesters (ethyl acetylacetate or ethyl aroylacetates) [97,98]. We employed this method to obtain the NH compounds, which were subsequently treated with iodomethane in basic media to yield the *N*-methyl derivatives (Scheme 1). Derivatives **1**–**8** have already been described by us in two previous papers [76,77], and the remaining ones, **9**–**23**, are new. In the case of 4-fluorobenzene-1,2-diamine and 3,5-difluorobenzene-1,2-diamine, two isomers were formed, pairs **11**/**13** and **15**/**17**, respectively, being separated by column chromatography (see Experimental section).

**Figure 4:**
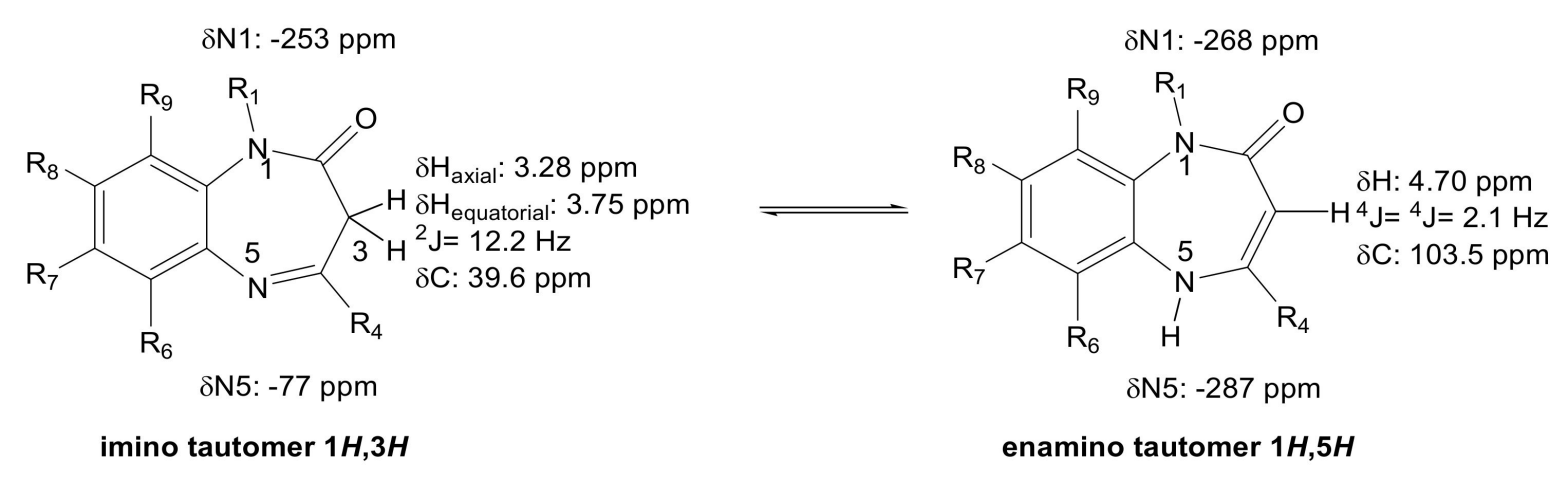
^1^H, ^13^C and ^15^N NMR data differentiating the imino and enamino tautomers of 1,5-benzodiazepin-2(3H)-ones.

All 1,5-benzodiazepin-2(3H)-ones were characterized by elemental analysis and multinuclear NMR spectroscopy, their purity being checked by differential scanning calorimetry. The ^1^H, ^13^C, and ^15^N NMR spectral data in DMSO-d6 confirmed that the NH compounds existed in the imino form 1H,3H. In some cases, compounds **9**, **11**, **13**,**15**, **22**, and **23** and the enamino tautomer 1H,5H was also detected in proportions lower than 5%, in accordance with what was previously observed in compounds **1**–**8** [76,77]. Figure 4 shows the mean values of the most relevant NMR features allowing us to identify both tautomeric forms.

### 3.2. Antioxidant (AOX) Properties

The 1,5-benzodiazepin-2(3H)-ones presented in this work were evaluated by three different methods to characterize their antioxidant (AOX) and antiradical capacity. There are several analytical methods available in the literature for antioxidant and antiradical capacity assessment, which are classified under two main types, i.e., H atom-donating ability (HAT) and electron transfer (ET) from antioxidant compounds, and both result in the neutralization of free radicals [99,100]. In the HAT assays, the antioxidant and substrate compete for radicals in a competitive reaction, and the 2,2-diphenyl-1-picrylhydrazyl hydrate (DPPH) method is one of the most commonly used. In the ET methods, the reductive capacity is measured through a colorimetric change that takes place when the radical is reduced by the antioxidant compound; the 2,2′-azino-bis (3-ethylbenz-thiazoline-6-sulphonic acid) (ABTS) [101,102] and the Ferric Reducing Antioxidant Power (FRAP) [79,103] assays are the most cited.

In the present work, the antioxidant activity of the compounds synthesized was determined by the ABTS, FRAP, and DPPH methods. The results are shown in Table 1 as IC_50_ values, expressed as the amount of antioxidant needed to decrease the initial radical concentration by 50%, and the Trolox Equivalent Antioxidant Capacity (TEAC) values are defined as the micromolar concentration of a Trolox solution having the same antioxidant capacity as a 100 µM solution of the substance. Trolox and curcumin were used as reference compounds for comparison purposes.

From Table 1, it can be noted that the N-methyl derivatives (**2**, **4**, **6**, **8**, **10**, **12**, **14**, **16**, and **19**) at position 1 of the 1,5-benzodiazepin-2(3H)-one ring have almost no antioxidant capacity in both ABTS and FRAP assays, which indicates that the amide hydrogen is required for the AOX activity. Moreover, the substitution of the methyl group by phenyl at position 4 of the 1,5-benzodiazepin-2(3H)-one ring in compound **1** yielded derivative 3, which lacks AOX activity. However, the introduction of F or Cl at the orto position of the 4-phenyl group of the 1,5-benzodiazepin-2(3H)-one ring yielded derivatives **5** and **7**, which had similar IC_50_s in the ABTS electron transfer assay to that of compound **1**, which could be explained by the steric hindrance that breaks the planarity of 4-phenyl with a 1,5-benzodiazepin-2(3H)-one ring. Compounds **18**, **20**, and **21** had the best inhibitory activity of the radical ABTS·+ of the whole chemical family, although their IC_50_ values were lower than those of curcumin and Trolox. The introduction of three methoxy groups at positions 3, 4, and 5 of the 4-phenyl ring in compound **20** slightly reduced its AOX capacity in comparison with derivative **18**, while the substitution of H by methyl groups at positions 7 and 8 of the 1,5-benzodiazepin-2(3H)-one ring improved the ABTS activity in compound **21**. On the other hand, the introduction of fluorine at positions 6, 7, 8, and 9 of the 1,5-benzodiazepin-2(3H)-one ring increased the IC_50_ in compounds **11**, **13**, **9**, **15**, **17**, and **3** in comparison with that of derivative **18**, with an AOX capacity in the order **18** >**11** ≈ **13** >**9** ≈ **15** >>**17** = **3**, i.e., with the inhibitory activity of the unsubstituted derivative >mono-fluor-substituted >di-fluor-substituted >tetra-fluor-substituted derivatives. From these results, it can be concluded that the introduction of fluorine in the 1,5-benzodiazepin-2(3H)-one ring reduced the AOX activity in this chemical series.

On the other hand, in the FRAP assay, the antioxidant character was evaluated as the reducing capacity of Fe^+3^ to Fe^+2^. Results shown in Table 1 demonstrate that although the present compounds followed similar structure-AOX-capacity relationships in this assay to those observed in the ABTS reduction test, the Fe^+3^-reducing power of the most potent derivatives **18**, **20**, and **21**, was just slightly lower than that seen for curcumin (i.e., curcumin >**18** ≈ **20** ≈ **21**). In this assay, only compounds with a phenyl group at position 4 of the 1,5-benzodiazepin-2(3H)-one ring had Fe^+3^-reducing capacity. Similarly, the fluorine substitution pattern showed a similar AOX trend to that identified in the ABTS assay, with the compounds with greater reductive power in the order **18** >**11** ≈ **13** >**9** ≈ **15** >>**17** = **3**.

Concerning the DPPH assay, which measures the AOX capacity of compounds capable of donating a hydrogen atom or an electron (HAT) to the DPPH· radical [104,105], none of the present compounds showed any H-transfer reduction capacity (data not shown). This fact could be explained by the lack of a phenolic OH in their structure.

### 3.3. Calculation of Physicochemical Properties

A study of the physicochemical parameters for the compounds presented in this work was carried out to evaluate their potential drug-like properties. Calculated values are shown in Table 2, in comparison with those obtained for curcumin. The most important parameters studied were molecular weight (MW), lipophilicity (clogP), predicted clogD (distribution coefficient at pH 7.4), HBD, HBA, N-Arom-Rings, NRotB, and predicted BBB and solubility. From these data, it can be concluded that in agreement with the most validated criteria for compounds intended to reach the central nervous system (CNS), most of the derivatives with some AOX capacity have a reasonable profile, with MW ≤ 360, clogP ≤ 3.0, cLogD ≤ 2, HBD ≤ 2, N-Arom-Ring ≤ 2, [106,107]; compounds **18** and **20** are the most interesting ones due to their lower values of MW (**18**: 236.275; **20**: 326.354) and logD_7.4_ (**18**: 2.081; **20**: 1.799) in comparison with those of curcumin (MW: 368.39; logD_7.4_ =2.778).

Regarding the potential for blood–brain barrier (BBB) penetration capacity, the Total Polar Surface Area (TPSA) has been used as a predictor [108], as drugs aimed at the CNS tend to have lower polar surface areas. The TPSA for a molecule to penetrate the brain has to be >40 Å^2^ and ≤90 Å2, in agreement with the currently most accepted range [106,107]. The calculated TPSA for these compounds shows that most of them have values in the range needed for BBB penetration and lower than that for curcumin. In the case of compounds **18** and **20** with AOX activity, their calculated TPSA values (**18**: 41.46; **20**: 69.15) were lower than that of curcumin (TPSA: 96.2), which suggests a potentially better capacity of **18** and **20** to cross the BBB.

### 3.4. Determination of Kinetic Solubility

Solubility in biorelevant aqueous media is one of the most important physicochemical parameters in drug discovery, being a determinant for non-toxic and brain-permeable drug-like compounds with good bioavailability. Therefore, compounds with good aqueous solubility are the best candidates for drug-discovery progression. In order to know the solubility of the most interesting 1,5-benzodiazepin-2(3H)-ones, we determined their kinetic solubility in aqueous media at different concentrations following the procedure described by Hoelke et al. [85]. The assays were performed at room temperature, in 50 mM Phosphate-Buffered Saline (PBS) from compound stocks in DMSO at 20 mM concentration. The maximum solubilities achieved in 50 mM PBS for all the newly synthesized derivatives and curcumin are shown in Table 3. Among those compounds with the best AOX profile, derivatives **18**, **20**, and **21** showed the best solubility behavior, while compounds **9**, **11**, and **13** were more insoluble in this aqueous media. It is worthwhile highlighting that as a general trend, the introduction of the methyl group at position N1 increased the solubility of the resulting methylated derivatives (**19** >**18**; **16** >**15**; **14** >**13**; **12** >**11**; **10** >**9**; **8** >**7**; **6** >**5**; **4** >**3**) in perfect agreement with the variations in the melting points observed (Table 3), which may indicate that there is a distortion in the crystal lattice or in the hydrogen bond network after N-methylation with a reduction in the crystal energy packing [109,110]. On the other hand, the introduction of fluorine atoms in the aromatic ring of the 1,5-benzodiazepin-2(3H)-ones reduced the solubility as can be seen in those fluoro-derivatives of compound **18** (**18** >**17** = **9** >**11** = **13** >**15**). Regarding curcumin, its solubility value determined in PBS is in agreement with previously reported very low water solubility (<0.6 µg/mL) [111,112,113].

In parallel, different in silico models were used to test their predictive power for the solubility of 1,5-benzodiazepin-2(3H)-ones [112,113,114]. However, as shown in Table 3, from these data it was concluded that in general, the solubility class assignment from the Silicos-IT model showed the best correlation with experimental data for this chemical series.

### 3.5. Chemical Reactivity with H_2_O_2_ and MTT

#### 3.5.1. Chemical Reactivity with H_2_O_2_

Basic chemistry shows that H_2_O_2_ reacts as an oxidant with specific functional groups [115,116]. Hundreds of articles and books have reviewed the chemical features and reaction conditions needed for the reaction of H_2_O_2_ as an oxidant [117]. After reviewing the literature, we concluded that none of them are present in the current 1,5-benzodiazepin-2(3H)-ones, and that reaction with H_2_O_2_ in aqueous media and buffers will require conditions and chemical reactants not present in the assays reported in this work.

**Figure 5:**
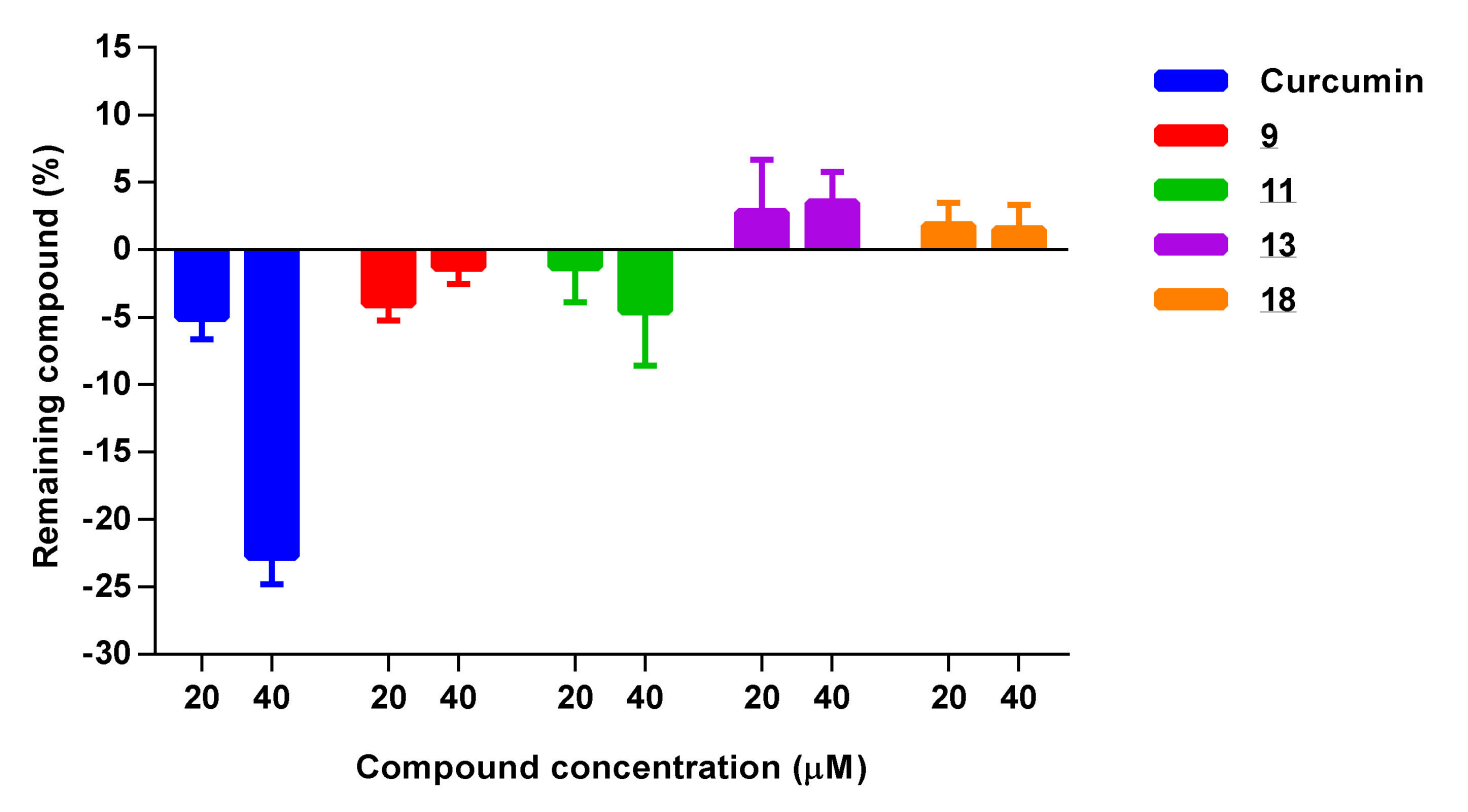
Reactivity of curcumin and compounds **9**, **11**, **13**, and **18** with H_2_O_2_ 500 µM in DMEM:F12 medium (without cells, FBS, and phenol red) at 37 °C for 4 h. Results represent the concentration decrease (%) for each compound. Values are the mean ± SD of two experiments performed on different days and carried out in quadruplicate in 96-well culture plates.

In our case, before running the protection experiments under oxidative stress conditions, we carried out experiments to assess the potential reactivity of compounds **9**, **11**, **13**, and **18** at four different concentrations (5, 10, 20, and 40 µM) with H_2_O_2_ 500 µM at 37 °C for 4 h in DMEM:F12 (without phenol red, FBS, and cells). Curcumin was used as a control compound. Figure 5 shows the results obtained at the highest concentrations (20 and 40 µM). From these data, it can be concluded that 1,5-benzodiazepin-2(3H)-ones did not show any significant reaction with H_2_O_2_ 500 µM after 4-h incubation at 37 °C, while curcumin, as widely reported in the literature, is unstable in buffers and other aqueous media [118,119] mainly due to its autoxidation capability, which is also enhanced in the presence of H_2_O_2_ with a 30% concentration decrease at pH ≥ 7.4 driven by hydrogen abstraction from the phenolic hydroxyl group [119,120].

#### 3.5.2. Chemical Reactivity with MTT

MTT is one of the most commonly used assays to study cell viability. Only viable cells reduce the MTT reagent to formazan by their NAD(P)H-dependent oxidoreductase enzymes [121,122]. MTT crosses the plasma membrane intact and is reduced intracellularly [123], which allows us to measure the metabolic processes linked to intracellular respiration that cause ER and mitochondria dysfunction through an increase in ROS generation, making MTT appropriate for neuroprotection measurements under oxidative stress conditions induced by electron transport inhibitors such as MPP^+^, 6OHD, rotenone, etc., which inhibit the electron transport complex I [124,125,126,127,128,129,130,131,132,133,134,135] and interfere with cellular redox equilibrium.

However, despite that MTT is a well-established method, some studies reported interferences with MTT because the assay was performed with extracts, nanoparticles, mixtures or metals [131,132,133,134,135,136,137,138,139]. Polyphenols with optical spectral interference are difficult in those situations to determine which are the real causes of interference, which may sometimes be due to impurities of those mixtures assayed.

**Figure 6:**
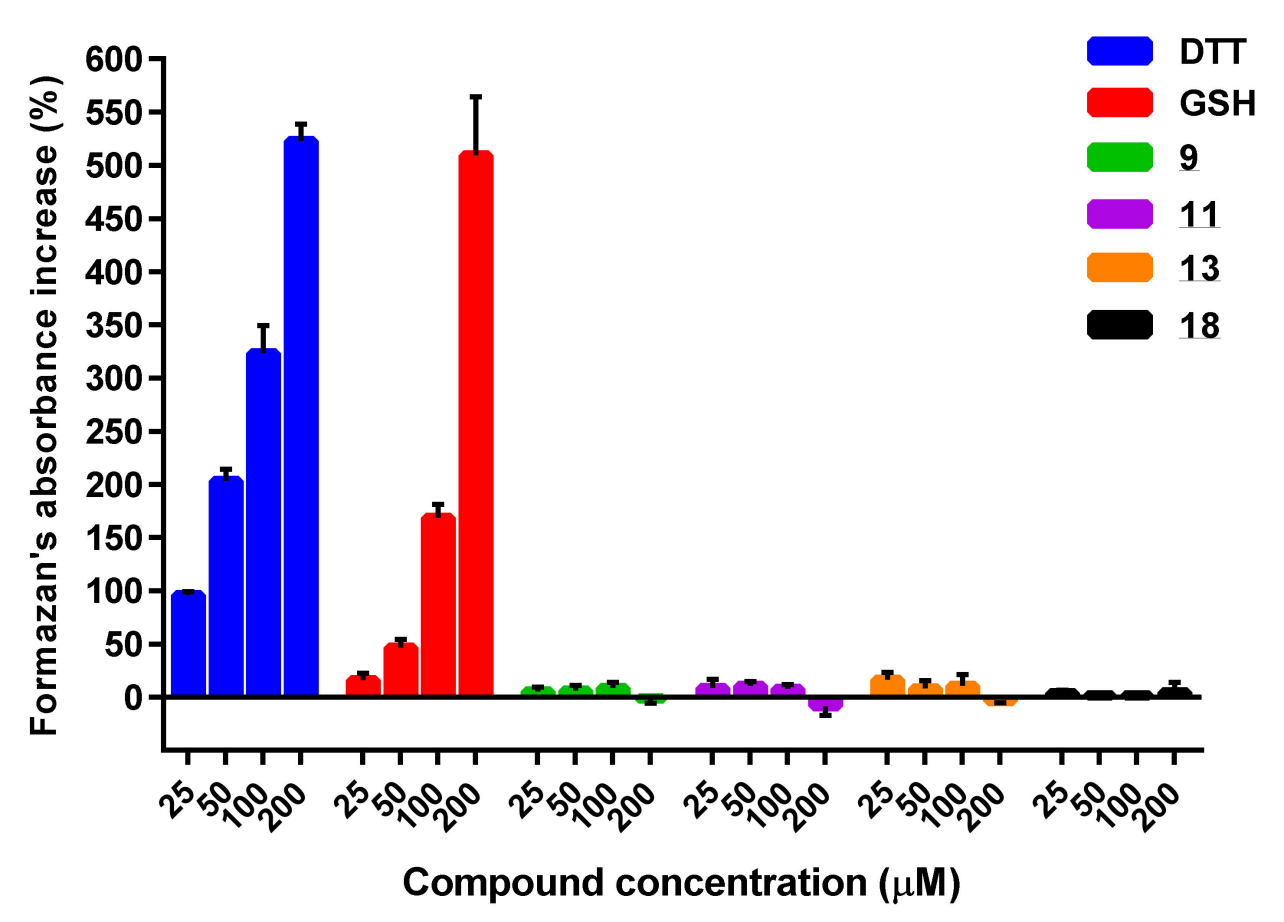
Reactivity study of dithiothreitol (DTT), glutathione (GSH), and compounds **9**, **11**, **13**, and **18** with MTT in DMEM: F12 medium (without cells, FBS, and phenol red) for 4 h at 37 °C. Results represent the absorbance increase (%) at 570 nm. Values are the Mean ± SD of two experiments performed on different days and carried out in quadruplicate in 96-well culture plates.

In this work, the 1,5-benzodiazepin-2(3H)-ones did not have protons to reduce the tetrazolium ring of MTT, as proven by the negative results obtained in the DPPH assay (Hydrogen Atom Transfer -HAT- AOX assay) previously mentioned. Nevertheless, for four of those compounds with the best AOX and calculated physicochemical properties (**9**, **11**, **13**, and **18**), their potential reactivity with MTT was studied before running the cytotoxicity and protection assays. For that, the derivatives were tested under the same assay conditions but without cells (in the absence of any metabolic reaction) to study any potential interference with MTT due to any reduction (GSH or DTT) or precipitation issues. In those tests, the compounds were incubated 4 h at 37 °C with MTT in the medium (DMEM: F12) without phenol red, using dithiothreitol (DTT) and glutathione (GSH) as positive interfering (control) compounds. No significant reaction was detected after 4 h incubation with MTT, as can be seen in Figure 6. From these results, it could be concluded that no interference with MTT readout would be expected from 1,5-benzodiazepin-2(3H)-ones.

### 3.6. Cytotoxicity Studies

Cytotoxicity studies were carried out in SH-SY5Y and HepG2 cell lines. The human-derived neuroblastoma cell line SH-SY5Y is a well-validated neuronal cell line that has been widely used as an in vitro model for Alzheimer’s and Parkinson’s disease studies [140,141,142,143,144,145,146]. Therefore, to study the neuroprotectant potential of benzodiazepines in the SH-SY5Y cell line, we first needed to determine their cytotoxicity in the same cellular model as well as in other human cell lines. On the other hand, HepG2 is the most well recognized and widely studied human hepatoma cell line as an in vitro model for chemical risk assessment in drug discovery studies for cytotoxicity, hepatotoxicity, metabolism, and genotoxicity [142,143].

Table 4 shows the cytotoxicity results obtained in the SH-SY5Y and HepG2 cell lines for compounds reported in this work, expressed as IC_50_ values (µM), i.e., the compound concentration needed to reduce the cell viability to 50% from the control. From these data, curcumin had the worst toxicity profile against SH-SY5Y and HepG2 cell lines in comparison with any of those 1,5-benzodiazepin-2(3H)-ones assayed. The curcumin cytotoxicity IC_50_ value in SH-SY5Y was in perfect agreement with previously reported data [144,145] as well as for the IC_50_ value determined in HepG2 cell line [146,147,148]. Among 1,5-benzodiazepin-2(3H)-ones, compounds **7** and **8** had higher toxicity in comparison with their other analogs, which could be related to their highest lipophilicity in terms of clogP (**7**:3.593; **8**:3.825) and LogD (**7**:3.101; **8**:3.287). As a general trend, most of the new derivatives showed lower cytotoxicity in these cell lines compared with curcumin. In addition to that, none of those compounds with the most interesting AOX profile (**9**, **11**, **13**, **18**, **20**, and **21**) showed cytotoxicity issues in both cell lines.

Microscopy images taken from cytotoxicity experiments (Figure S1) highlighted the best cytotoxicity profile of **18** in comparison with that one from curcumin.

### 3.7. Neuroprotection Studies

#### 3.7.1. Neuroprotection against H_2_O_2_-Induced Oxidative Stress

Neuroprotection assays were carried out in the SH-SY5Y cell line under oxidative stress conditions to study the neuroprotectant character of the new 1,5-benzodiazepin-2(3H)-ones. In this in vitro model, SH-SY5Y cells are treated with H_2_O_2_ as the stressor to evaluate the potential neuroprotective activity of new compounds with AOX properties.

**Figure 7:**
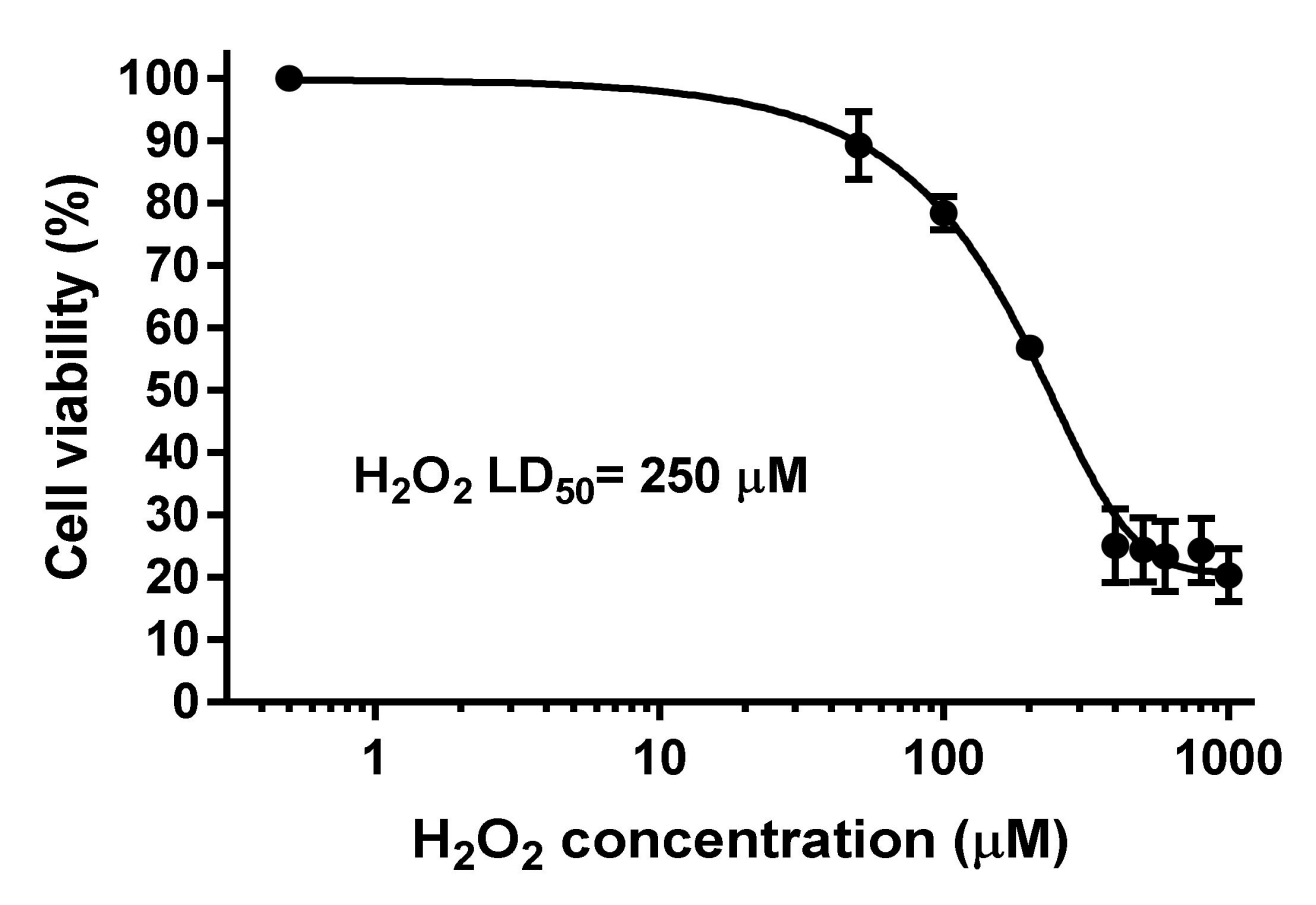
Determination of the H_2_O_2_ lethal dose (LD50, µM) after 24-h incubation for SH-SY5Y cells. Values are the Mean ± SD of six different experiments carried out in quadruplicate (r2: 0.9879).

In the present work after cell treatment with AOX compounds for 24 h, cells were exposed to a H_2_O_2_ lethal dose (LC50). The H_2_O_2_ EC50 in the SH-SY5Y cell line was previously determined from the killing H_2_O_2_ curve as shown in Figure 7; 250 µM was the concentration at which the cell viability remained at 50% of the control.

As it has been widely reported from a large body of experimental data related to the oxidative stress increase during aging, the H_2_O_2_ concentration needed for cell growth arrest and apoptosis ranges from 10^−6^ to 10^−4^ Molar [149]. Among relevant ROS, H_2_O_2_ has the lowest reactivity and the highest stability (seconds). Lower intracellular H_2_O_2_ concentrations (10^−8^ to 10^−7^) only affect cell proliferation physiology, acting as a signaling molecule and producing oxidative eustress without oxidative damage [150,151]. To achieve intracellular physiological H_2_O_2_ concentrations that can drive oxidative stress, mitochondrial dysfunction, and induce apoptosis, mammalian cells have to be treated with H_2_O_2_ concentrations in the range from 200–1000 µM, as has been nicely demonstrated by Huang et al., [152] for levels similar to the H_2_O_2_ concentration determined as EC50 in this work for SH-SY5Y cells and such as those reported by other authors for the same cell line [153,154,155,156].

For neuroprotection experiments, SH-SY5Y cells were treated with test compounds at different concentrations (5, 10, 20, 30, and 40 µM) for 24 h. After the treatment period, cells were exposed to H_2_O_2_ 250 µM for 24 h. At the end of the treatment, cell viability was measured with MTT. Table 5 shows the results obtained for all the compounds synthesized in an initial screening using curcumin as a reference. These data confirmed that only those compounds with AOX properties had some neuroprotectant activity and that all the N1-methylated derivatives were inactive, which highlights the key role of NH in biological activity.

**Figure 8:**
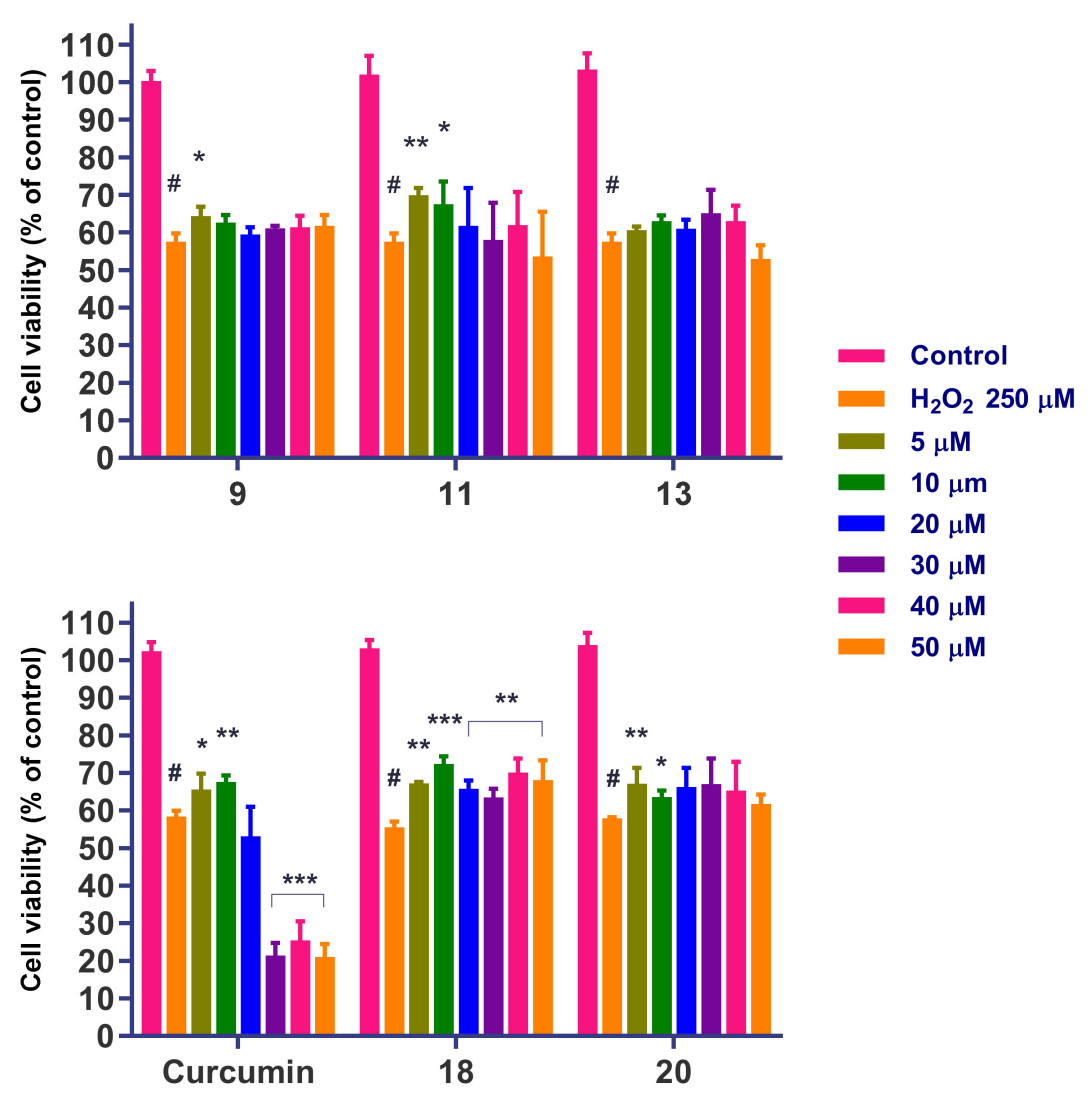
Neuroprotection effect of compounds **9**, **11**, **13**, **18**, **20**, and curcumin against H_2_O_2_-induced cytotoxicity in SH-SY5Y cells. Cell viabilities were determined by MTT assay. Compounds were administered at different concentrations for 24 h prior to treatment with H_2_O_2_ 250 µM for another 24 h. Data are presented as the Mean ± SEM of four independent experiments carried out in quadruplicate. # *p* < 0.001 vs. vehicle-treated control. * *p* < 0.05, ** *p* < 0.01, *** *p* < 0.001 vs. only with H_2_O_2_-treated cells.

Figure 8 displays the neuroprotectant activity for the most interesting derivatives measured as cell viability results (%) obtained for compounds **9**, **11**, **13**, **18**, **20**, and curcumin in comparison with control cells at 5, 10, 20, 30, 40, and 50 µM. As shown, the decreased SH-SY5Y cell viability produced by 250 µM H_2_O_2_ was significantly improved by pretreatment with compounds **18** and **20** at all the concentrations assayed; compound **18** had the best cell death inhibition profile at 10 (*p* <0.01), 20, 30, 40, and 50 µM (*p* <0.001). It is worth highlighting that **18** and **20** without fluorine in the aromatic ring of the 1,5-benzodiazepin-2(3H)-ones were the most active derivatives. However, in the same assay, curcumin only showed slight protection at concentrations 5 and 10 µM, which is in agreement with previously reported data [74,157] carried out with H_2_O_2_ at 100 and 250 µM. On the other hand, compound **11** showed high variable neuroprotection results at concentrations higher than 10 µM due to solubility issues. These results confirm that compounds with the best AOX capacity yielded cell protectant activity under oxidative stress pressure. The microscopy images in Figure S2 highlight the neuroprotection differences between derivative **18** and curcumin.

In addition to the MTT cell viability assays, the neuroprotection effect on cell death induced by 250 µM H_2_O_2_ was determined by measuring the lactate dehydrogenase (LDH) release in the cell culture medium. The LDH assay in this work was performed as a secondary assay to confirm those results obtained previously from the MTT test, following the indications from the kit’s supplier. The LDH release (LDHr) assay [158] was used as a surrogate marker of cell viability and for the assessment of neuroprotection and in agreement with the procedure used in previously reported studies. In this work, the LDHr data were normalized to 100% as the value for the control cells without any treatment.

**Figure 9:**
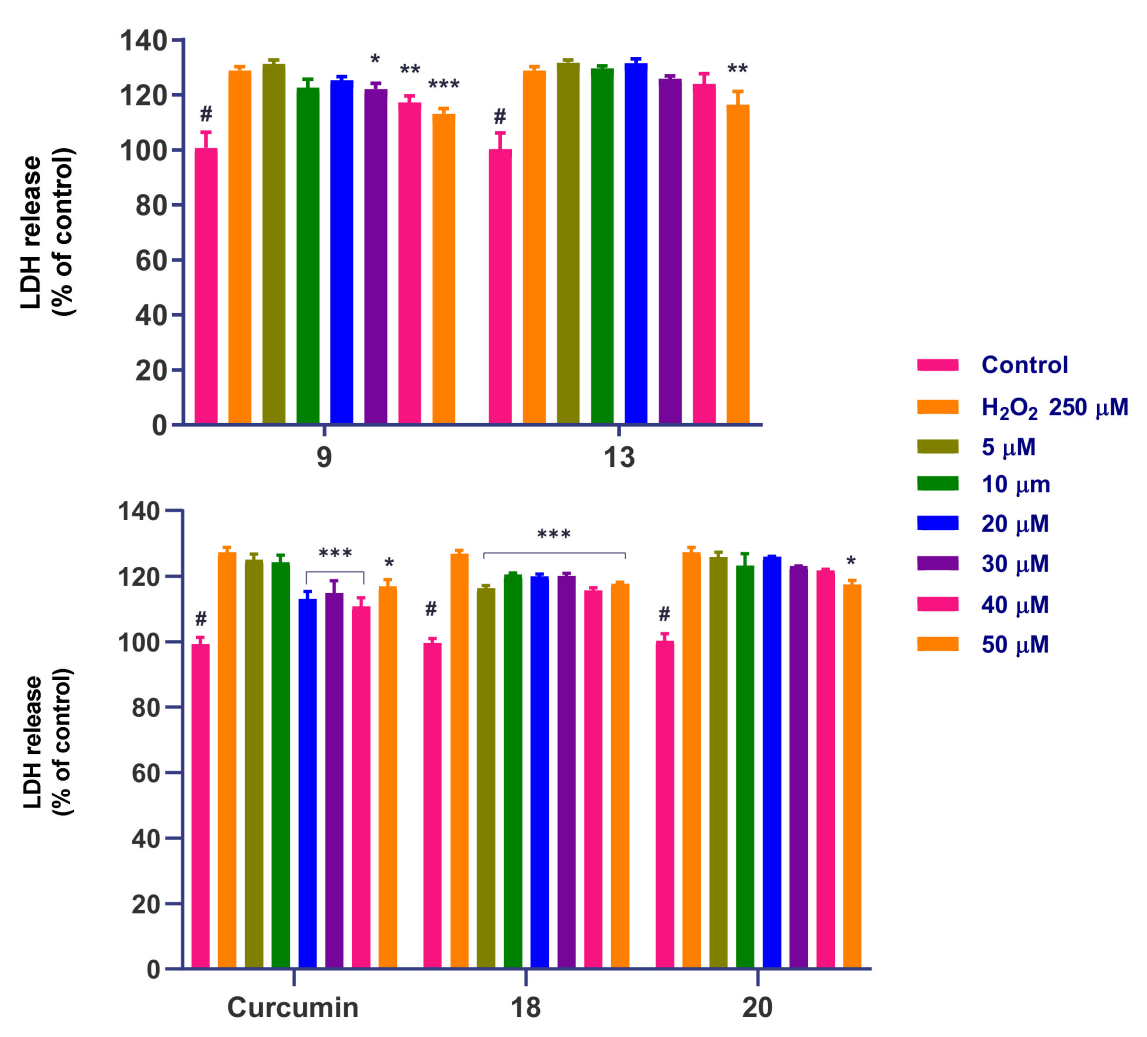
Neuroprotection effect of compounds **9**, **13**, **18**, **20**, and curcumin against H_2_O_2_-induced cytotoxicity in SH-SY5Y cells expressed as LDH release. Compounds were administered at different concentrations for 24 h prior to H_2_O_2_ 250 µM treatment for 24 h. Data are presented as the Mean ± SEM of three independent experiments carried out in quadruplicate. # *p* < 0.001 vs. vehicle-treated control. * *p* < 0.05, ** *p* < 0.01, *** *p* < 0.001 vs. only with H_2_O_2_-treated cells.

As shown in Figure 9, compounds **9**, **20**, and curcumin reduced LDH leakage to some degree, but only derivative **18** showed a significant reduction at all the concentrations tested (*p* < 0.001).

Taking into account all the previous AOX, physicochemical, cytotoxicity, solubility, and neuroprotection results, 1,5-benzodiazepin-2(3H)-ones derivatives **18** and **20** are the most interesting compounds for further studies that evaluate the rational bases of their neuroprotection profile.

**Figure 10:**
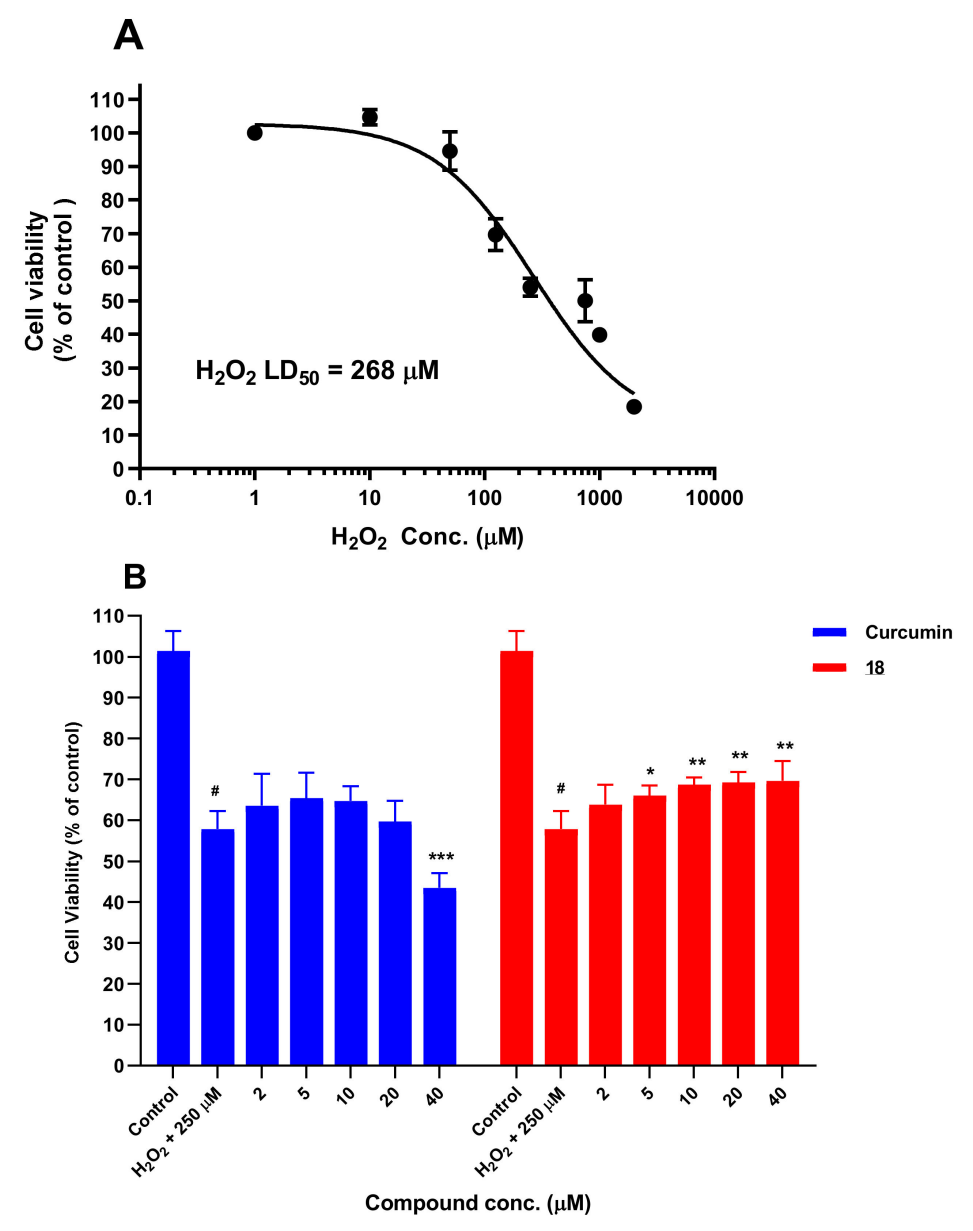
(**A**) Determination by the Resazurin method of the H_2_O_2_ lethal dose (LD50, μM) after 24-h incubation of SH-SY5Y cells. Values are the Mean ± SEM of four different experiments carried out in quadruplicate. (**B**) Neuroprotection effect of curcumin and compound **18** against H_2_O_2_-induced cytotoxicity in SH-SY5Y cells. Cell viabilities were determined by Resazurin assay with fluorometric detection. Compounds were administered at different concentrations for 24 h prior to H_2_O_2_ 250 µM treatment for another 24 h. Data are presented as the Mean ± SEM of four independent experiments carried out in quadruplicate. # *p* < 0.001 vs. vehicle-treated control. * *p* < 0.05, ** *p* < 0.01, *** *p* <0.001 vs. only with H_2_O_2_-treated cells.

To confirm the neuroprotection results obtained with the MTT method, the most active compound **18** and curcumin (as a control) were also evaluated by the resazurin assay with fluorometric detection, as an additional way to measure cellular metabolic activity [82,121]. Similar to the procedure carried out with MTT, the EC_50_ for H_2_O_2_ was determined from a killing curve as shown in Figure 10A. The EC_50_ value obtained was 268 µM, in close agreement with the previous value determined by the MTT method. The protection results for curcumin and **18** are shown in Figure 10B using H_2_O_2_ 250 µM to compare with those obtained with the MTT method. The data show that compound **18** yielded significant protection against the oxidative stress insult in comparison with curcumin.

**Figure 11:**
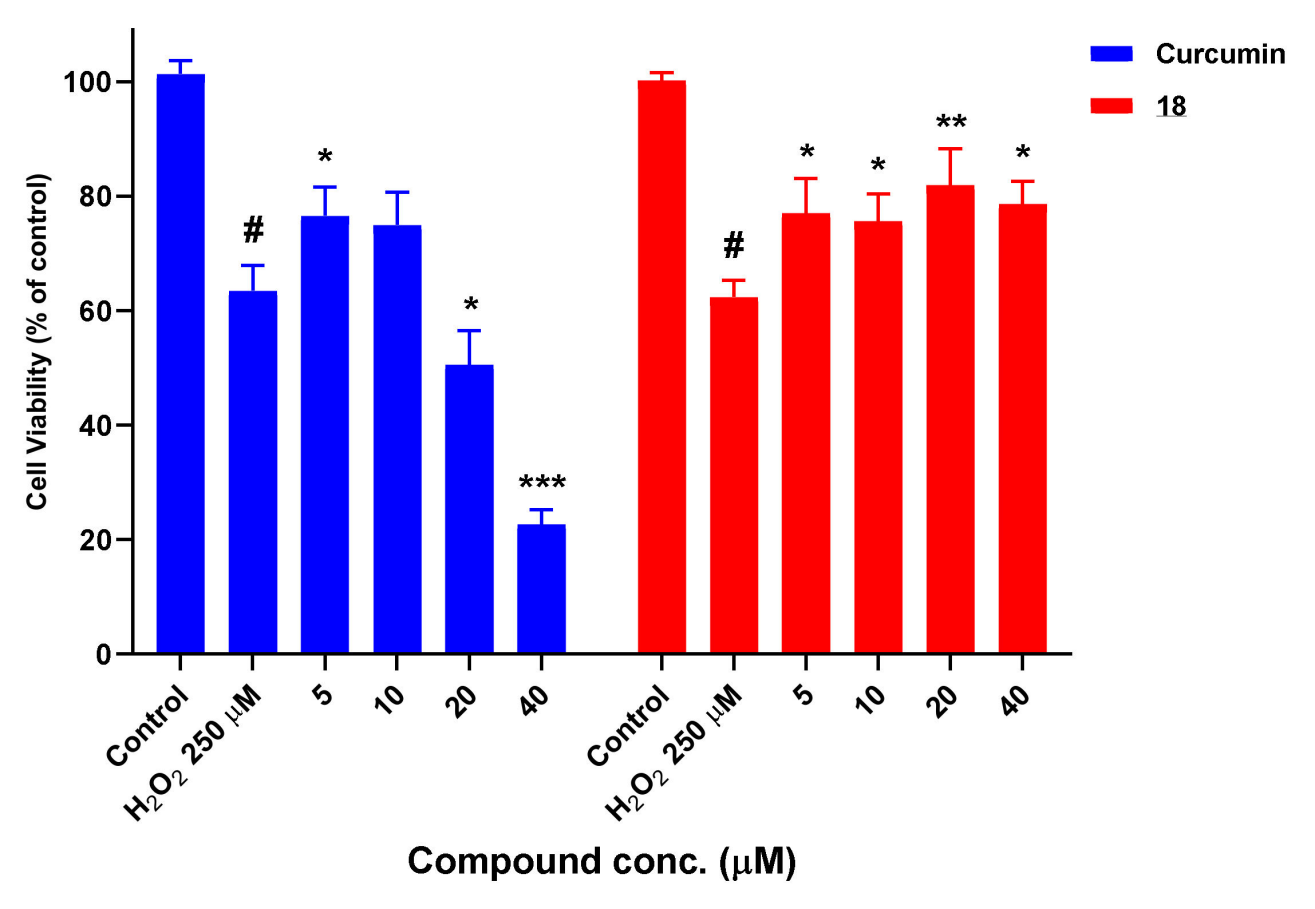
Neuroprotection effect of curcumin and compound **18** against H_2_O_2_-induced cytotoxicity in SH-SY5Y cells. Cell viabilities were determined by Calcein–AM assay with fluorometric detection. Compounds were administered at different concentrations for 24 h prior to H_2_O_2_ 250 µM treatment for another 24 h. Data are presented as the Mean ± SEM of four independent experiments carried out in quadruplicate. # *p* < 0.001 vs. vehicle- treated control. * *p* < 0.05, ** *p* < 0.01, *** *p* < 0.001 vs. only with H_2_O_2_-treated cells.

**Figure 12:**
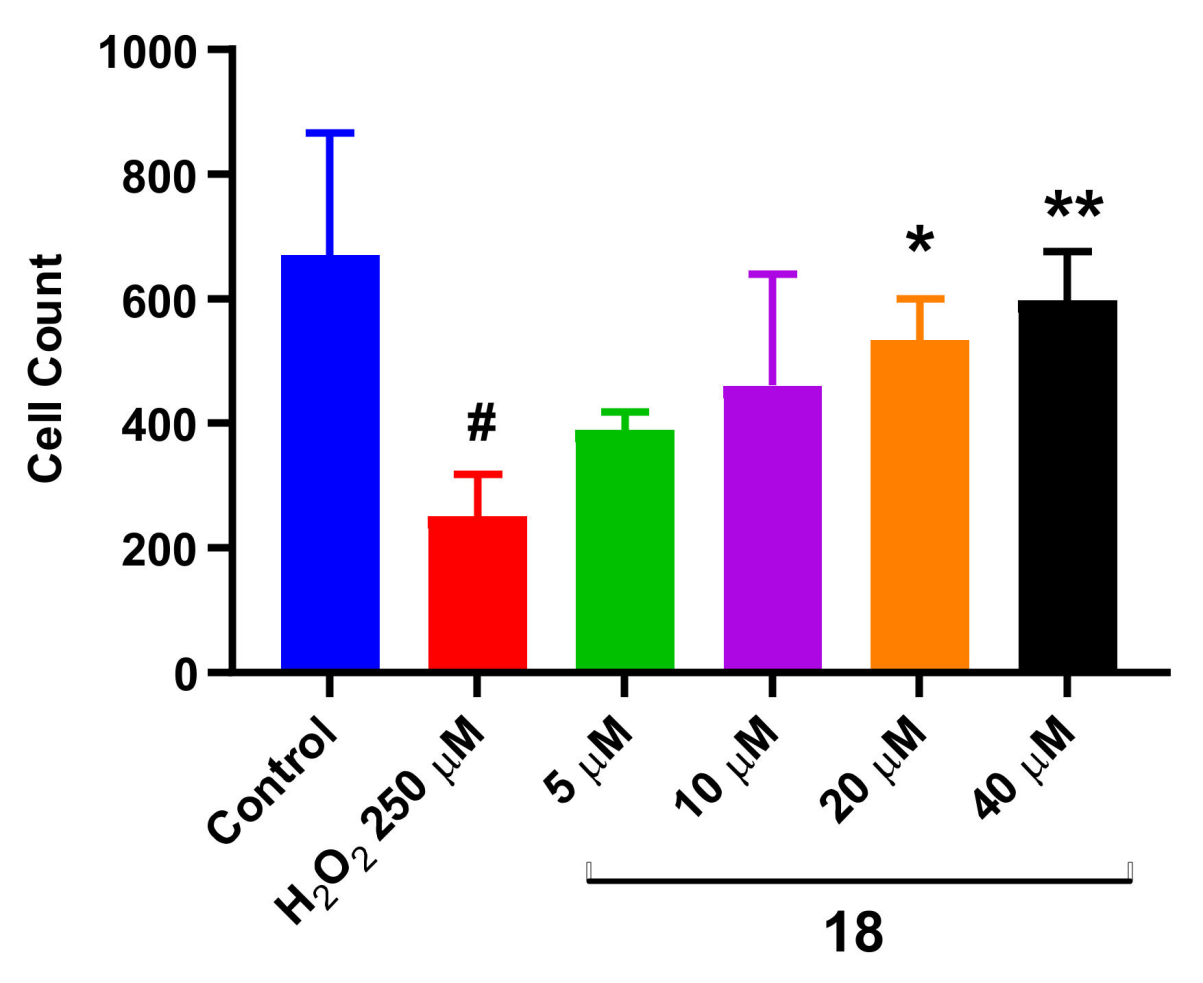
Neuroprotection effect of compound **18** against H_2_O_2_ induced cytotoxicity in SH-SY5Y cells. Compound was administered at different concentrations for 24 h prior to H_2_O_2_ 250 µM treatment for another 24 h. A. After treatment, Calcein-AM was added and cells were counted from six fluorescent microscopy random images taken at 4× from four different replicates of each compound concentration. ImageJ was used for cell counting. # *p* < 0.001 vs. vehicle- treated control. * *p* < 0.05, ** *p* < 0.01 vs. only with H_2_O_2_-treated cells.

To corroborate the neuroprotection results obtained with the MTT and resazurin methods, the most active compound **18** and curcumin were also assayed using Calcein-AM to quantify cell viability under oxidative stress pressure induced by 24-h treatment with H_2_O_2_ 250 µM. Calcein-AM is a membrane permeant, not a fluorescent compound. Only live cells with intact plasma membrane have esterases that hydrolyze the compound into the green fluorescent dye calcein, which is retained in the cytoplasm [83,84]. The fluorescent signal obtained is proportional to the number of living cells in the sample. Figure 11 shows the data obtained from SH-SY5Y cells after 24-h treatment with curcumin and **18** and after 24-h treatment with H_2_O_2_. Cell counting results from random images taken by fluorescent microscopy are represented in Figure 12, and they confirm the statistically significant neuroprotectant activity of **18** at 5, 10, 20, and 40 µM. Microscopy images in Figure S3 show the neuroprotection activity observed with Calcein-AM for derivative **18**.

Taking into account all the previous experiments performed by different methods, we can conclude that 1,5-benzodiazepin-2(3H)-ones are an interesting chemotype with neuroprotectant activity under oxidative stress conditions induced by H_2_O_2_; compounds **18** and **20 are** the best candidates for other assays.

#### 3.7.2. Neuroprotection Studies in the 6-OHD Neurotoxicity-Induced Model

Neuroprotection studies were carried out for compounds **18** and **20** in a model of neurotoxicity produced by 6-hydroxydopamine (6-OHD), a hydroxylated analog of dopamine generated by its oxidation, which is extensively used in vivo and in vitro to model PD [159,160]. Treatment with 6-OHD is associated with enhanced oxidative stress and mitochondrial dysfunction, which lead to dopaminergic neuron damage through different mechanisms [125,161,162].

**Figure 13:**
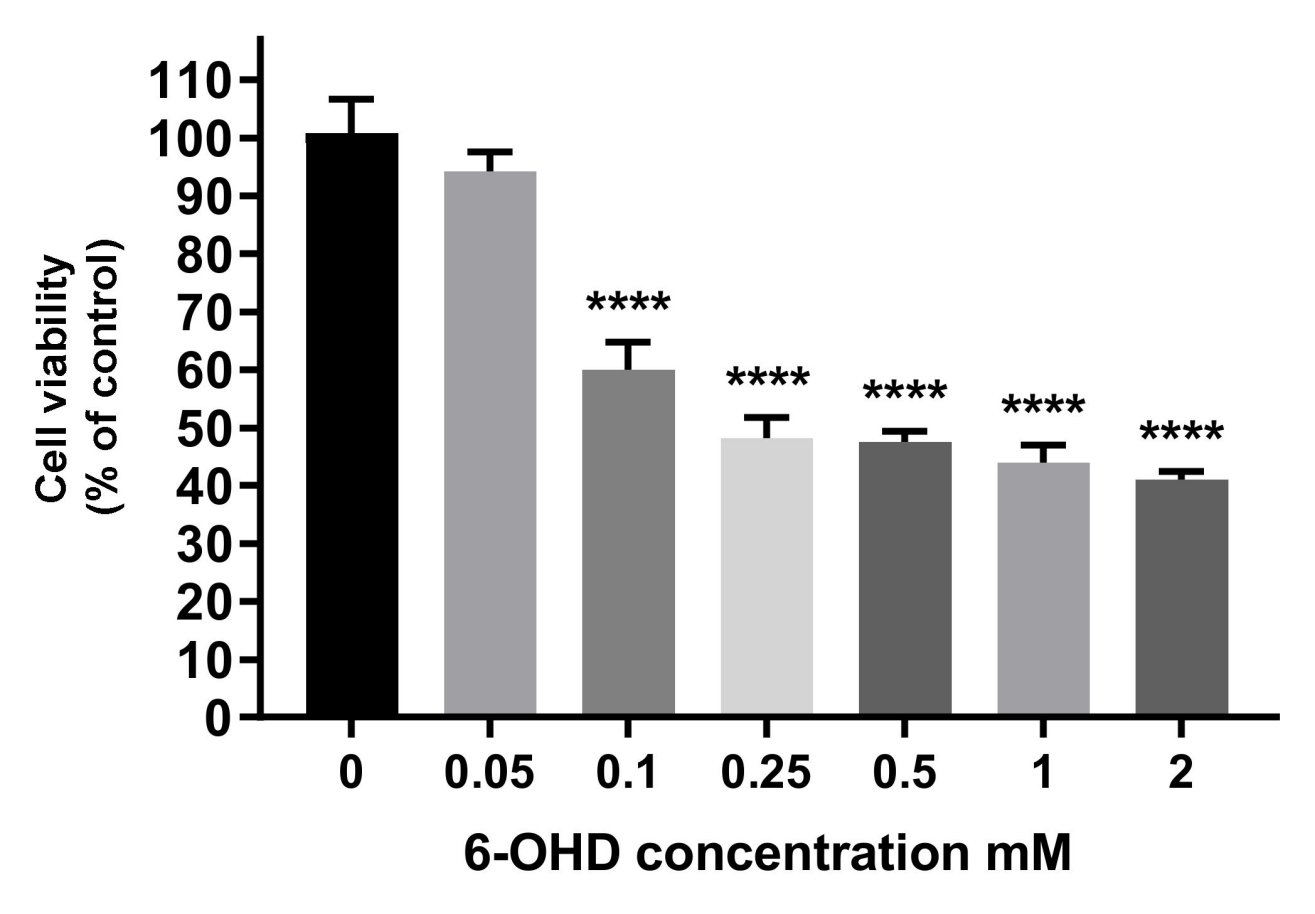
Effect of 6-OHD 48-h treatment on SH-SH5Y cells. Cell viability was measured by the MTT assay. Data are presented as the Mean ± SEM of three independent experiments carried out in quadruplicate. **** *p* < 0.0001 vs. vehicle-treated control cells.

**Figure 14:**
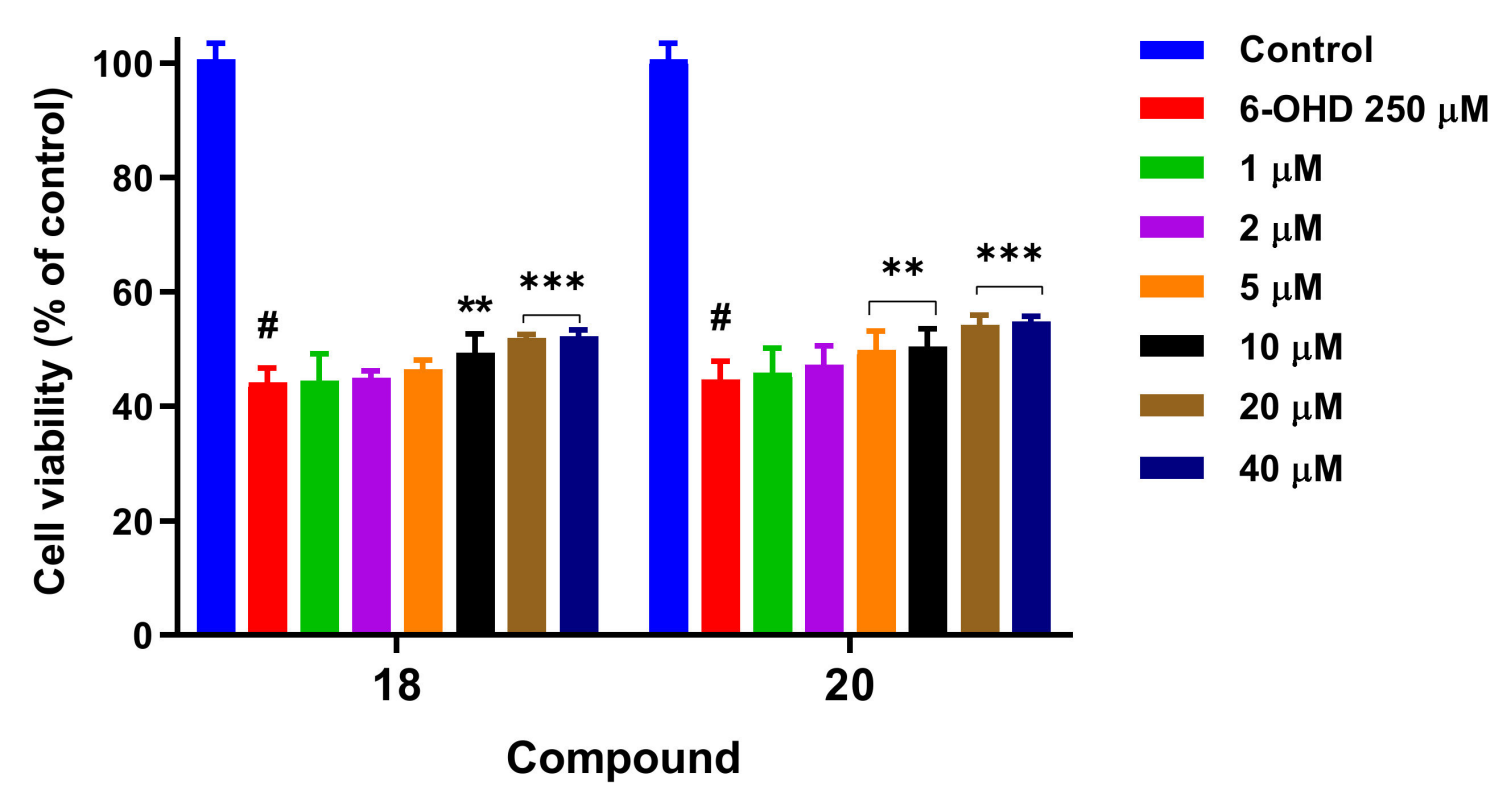
Neuroprotection effect of compounds **18** and **20** in the 6-OHD neurotoxicity-induced model in SH-SY5Y cells. Compounds were administered at different concentrations for 24 h before 6-OHD 250 µM treatment for 48 h. Data are presented as the Mean ± SEM of three independent experiments carried out in quadruplicate. # *p* < 0.001 vs. vehicle-treated control. ** *p* < 0.01, *** *p* < 0.001 vs. only 6-OHD treated cells.

Figure 13 shows that after 48 h of 6-OHD treatment, the oxidative stress reduced the SH-SY5Y cell viability in a dose-dependent manner from 0.05 to 2 mM; 0.25 mM was the concentration at which the cell viability was reduced by a 50%. Pretreatment with compounds **18** and **20** for 24 h before 6-OHD stress insult yielded significant neuroprotection in this demanding model (Figure 14). From this study, it can be concluded that both derivatives have a similar protection profile with increasing compound concentration (*p* <0.01 at 5 and 10 µM; *p* <0.001 at 20 and 40 µm). However, curcumin did not show any protection at the concentrations assayed (1, 2, 5, 10, 20, 40 µM) in this demanding assay (data not shown), which is in agreement with previous results [163], where 24-h treatment with curcumin (5, 10, and 20 µM) protected SH-SY5Y cells before exposure to only 25 µM 6-OHD for 24 h; these conditions were less challenging than the ones used in the present work (250 µM 6-OHD, 48 h).

#### 3.7.3. Neuroprotection Studies in the MPP^+^ Neurotoxicity-Induced Model

1-Methyl-4-phenylpyridinium (MPP^+^) is a positively charged metabolite of 1-methyl-4-phenyl-1,2,3,6-tetrahydropyridine (MPTP), which is toxic for dopaminergic neurons through the generation of oxidative stress and induction of apoptosis [164,165]. MPP^+^ has been widely used as an inducer of PD-like pathologies in several in vitro and in vivo PD models [166,167]. It has been characterized as an inhibitor of the mitochondrial electron transport complex I [164,165].

**Figure 15:**
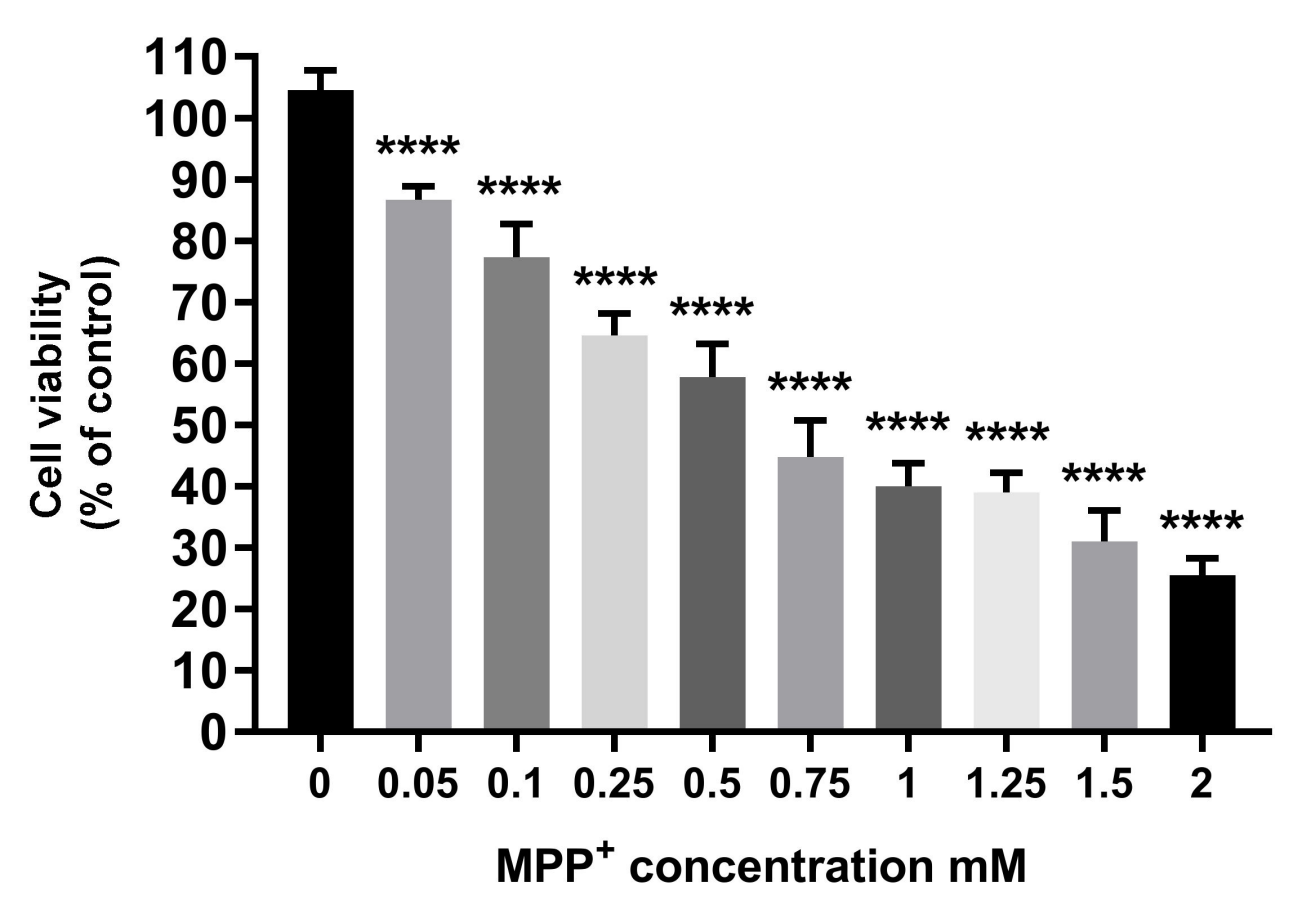
Effect of MPP^+^ treatment for 48 h on SH-SH5Y cells. Cell viability was measured by the MTT assay. Data are presented as the Mean ± SEM of three independent experiments carried out in quadruplicate. **** *p* < 0.0001 vs. vehicle-treated control cells.

In the present study, SH-SY5Y cells were treated with MPP^+^ at different concentrations from 0.05 to 2 mM for 48 h, and the cell viability was measured by the MTT method. Figure 15 shows the MPP^+^ concentration-dependent cell viability profile; 700 µM was the MPP^+^ concentration that resulted in 50% cell viability.

**Figure 16:**
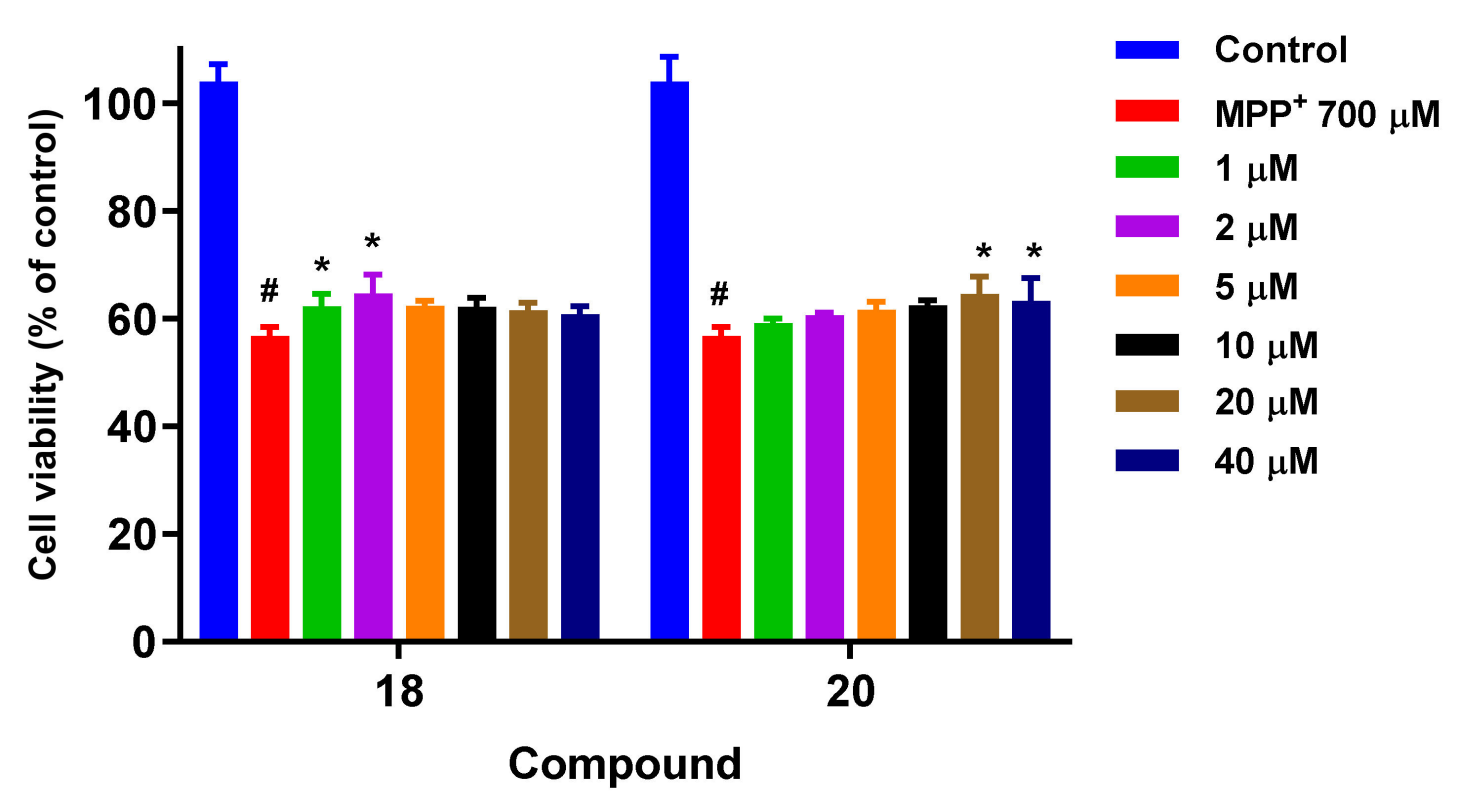
Neuroprotection effect of compounds **18** and **20** in the MPP^+^ neurotoxicity-induced model in SH-SY5Y cells. Compounds were administered at different concentrations 24 h before MPP^+^ 700 µM treatment for 48 h. Data are presented as the Mean ± SEM of three independent experiments carried out in quadruplicate. # *p* < 0.001 vs. vehicle-treated control. * *p* < 0.05 vs. only 6-OHD treated cells.

To evaluate the neuroprotection effect of compounds **18** and **20** in SH-SY5Y cells against MPP^+^ oxidative stress insult, cells were treated for 24 h with these compounds and then exposed to MPP^+^ 700 µM for 48 h. Figure 16 shows that compound **18** had a small significant neuroprotection at 1 and 2 µM (*p* <0.05) while derivative **20** demonstrated dose-dependent neuroprotection, which was more significant at 20 and 40 µM (*p* <0.05). However, under the same conditions, curcumin did not yield any protection in this assay (data not shown), although it has been reported that curcumin has shown some protective activity in a less demanding model (12-h treatment with MPP^+^ 400 µM) [168,169].

### 3.8. Effect on ROS Levels in SH-SY5Y Cells under Oxidative Stress

**Figure 17:**
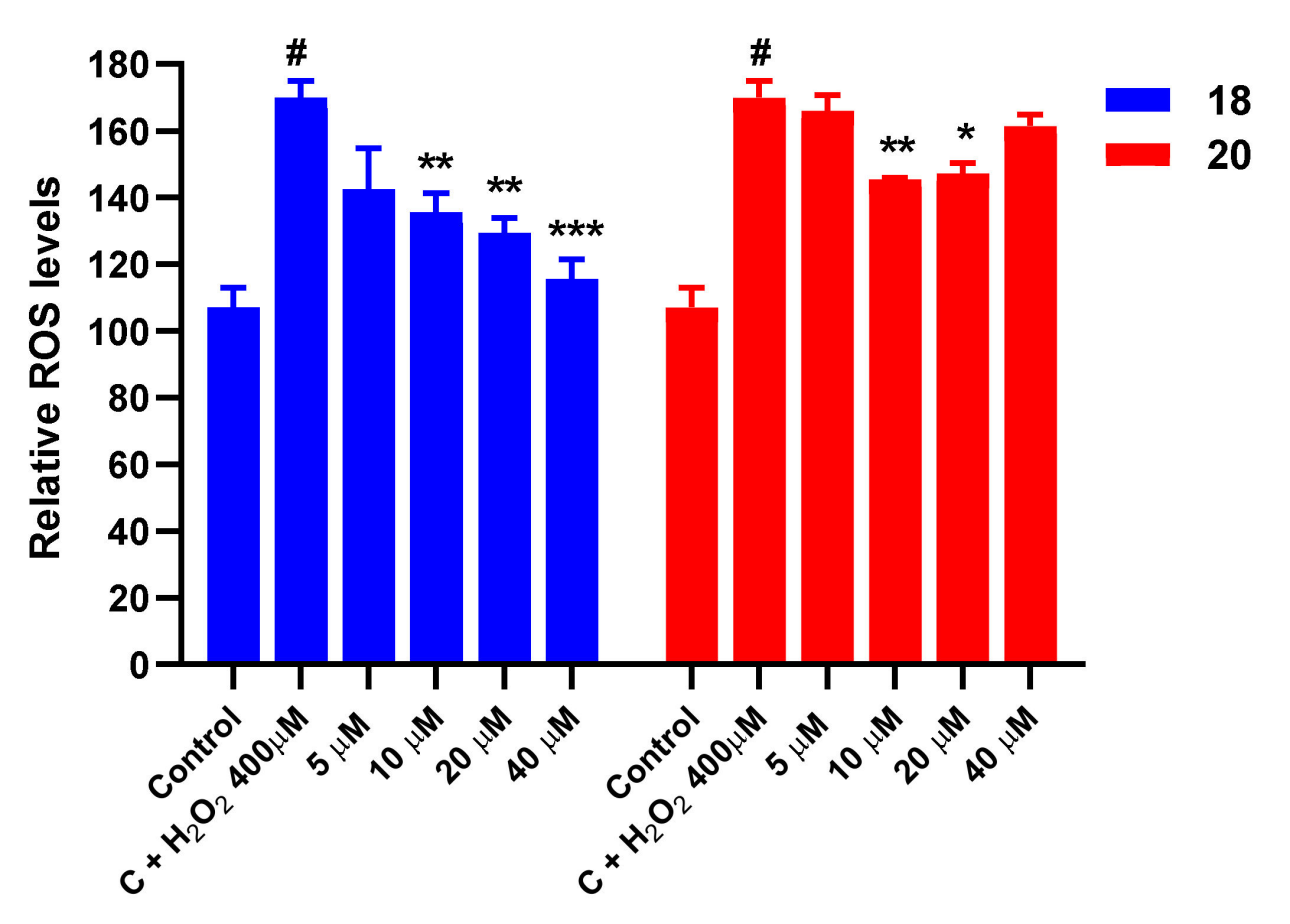
Intracellular ROS level reduction by compounds **18** and **20** in SH-SH5Y under 4-h treatment with 400 µM H_2_O_2_. Cells were treated with **18** and **20** for 24 h prior to exposure to H_2_O_2_. ROS levels were evaluated by the DFCH-DA method. Data are presented as the Mean ± SEM of three independent experiments carried out in quadruplicate. # *p* <0.001 vs. vehicle-treated control. * *p* < 0.05, ** *p* < 0.01, *** *p* < 0.001 vs. only with H_2_O_2_-treated cells.

Intracellular ROS levels increase under oxidative stress conditions, which may cause cell damage and death. Therefore, ROS production caused by H_2_O_2_ was studied in cells pretreated with or without derivatives **18** and **20**. In this assay, the DFCH-DA method was used to evaluate the ROS intracellular levels by fluorescence [86]. Figure 17 shows that a 24-h compound treatment prior to 4-h exposure to 400 µM H_2_O_2_ yielded a ROS level reduction at all doses of compound **18**, which was more significant at 10, 20, and 40 µM (*p* <0.01 and *p* <0.001). Compound **20** showed a lower ROS decrease that was more significant at 10 and 20 µM (*p* <0.01 and *p* <0.05, respectively). From this study, it could be concluded that although both **18** and **20** reduced intracellular ROS levels, derivative **18** had the most promising profile.

### 3.9. Protection against Mitochondrial Membrane (ΔΨm) Depolarization Induced by Oxidative Stress

Under oxidative stress conditions, depolarization of the Mitochondrial Membrane Potential ΔΨm (MMP) due to the generation of ROS has been proposed to participate in mitochondrial dysfunction and cellular apoptosis [38].

**Figure 18:**
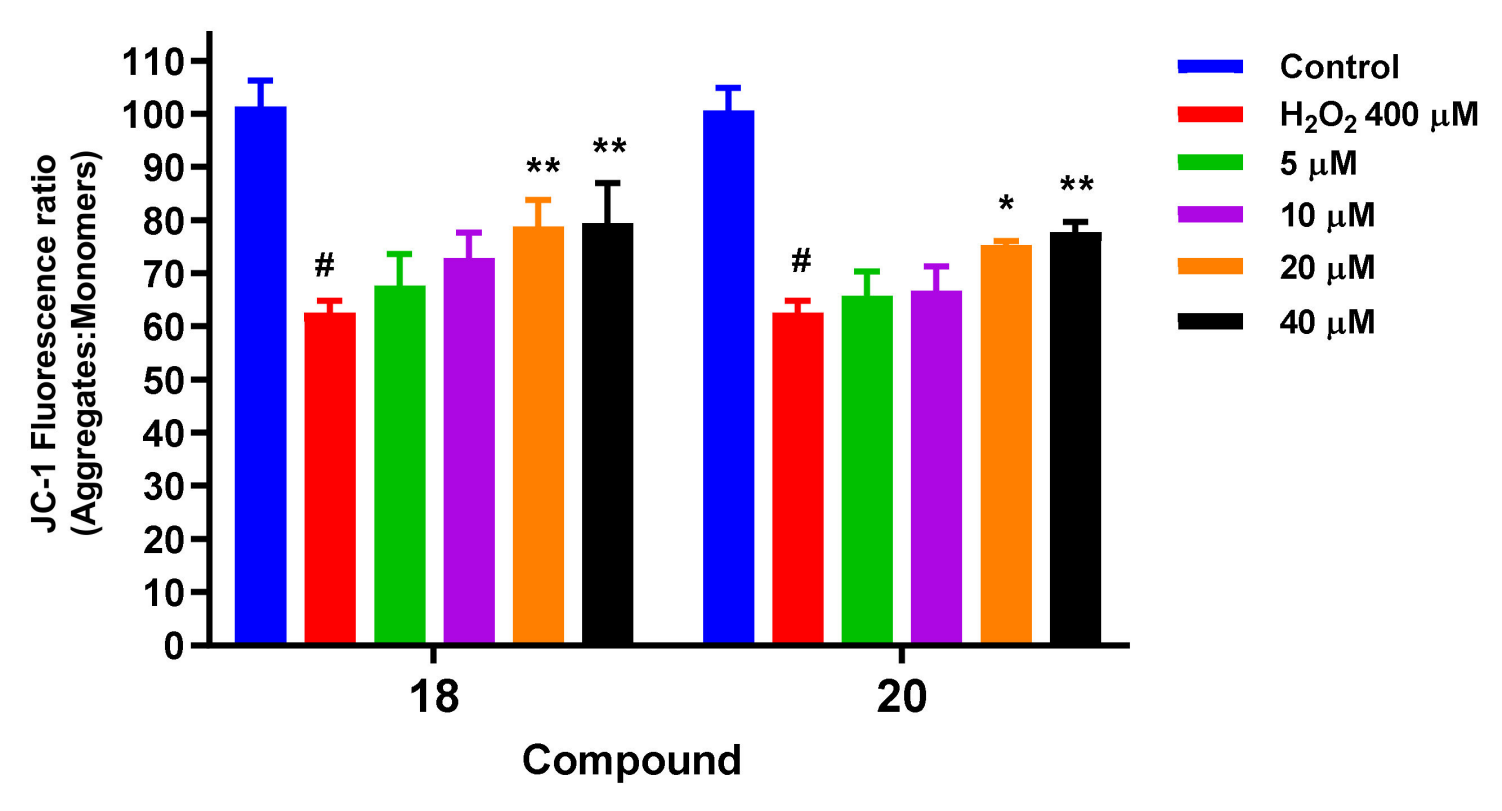
MMP loss is prevented by compounds **18** and **20** in SH-SH5Y under 4-h exposure to 400 µM H_2_O_2_. Cells were treated with **18** and **20** for 24 h prior to exposure to H_2_O_2_. MMP is expressed as the JC-1 fluorescence ratio in terms of red fluorescence to green fluorescence (Aggregates:Monomers). Data are presented as the Mean ± SEM of four independent experiments carried out in quadruplicate. # *p* < 0.001 vs. vehicle-treated control. * *p* < 0.05, ** *p* < 0.01 vs. cells treated only with H_2_O_2_.

In the present work, MMP experiments were carried out with the cationic dye JC-1 (5,5′,6,6′-tetrachloro-1,1′,3,3′-tetraethylbenzimidazolylcarbocyanine iodide) to determine whether benzodiazepines **18** and **20** protect the mitochondrial membrane from depolarization under H_2_O_2_ insult. JC-1 enters the mitochondria selectively and reversibly changes its color from red to green as the membrane potential decreases [89]. JC-1 forms red fluorescent J-aggregates in healthy cells but remains as a monomer with green fluorescence in apoptotic cells with low MMP. The ratio of J-aggregates to J-monomers is an indication of healthy non-apoptotic cells. As Figure 18 shows, in SH-SY5Y cells exposed to 400 µM H_2_O_2_ for 4 h, the MMP was reduced by about 40%, but those previously treated with **18** and **20** at different concentrations for 24 h had a significant MMP recovery of 10–25%. These results confirmed that the neuroprotective effects of **18** and **20** were in agreement with the reduction in the intracellular ROS levels and the MMP recovery under 400 µM H_2_O_2_ oxidative stress insult.

### 3.10. Determination of Mitochondrial Superoxide Levels with MitoSOX

MitoSOX Red is a derivative of dihydroethidium bearing a cationic triphenylphosphonium group, which is a positively charged molecule that rapidly accumulates in mitochondria and may be used to detect superoxide and ROS production inside the mitochondria by fluorometry, microscopy, and flow cytometry [88].

**Figure 19:**
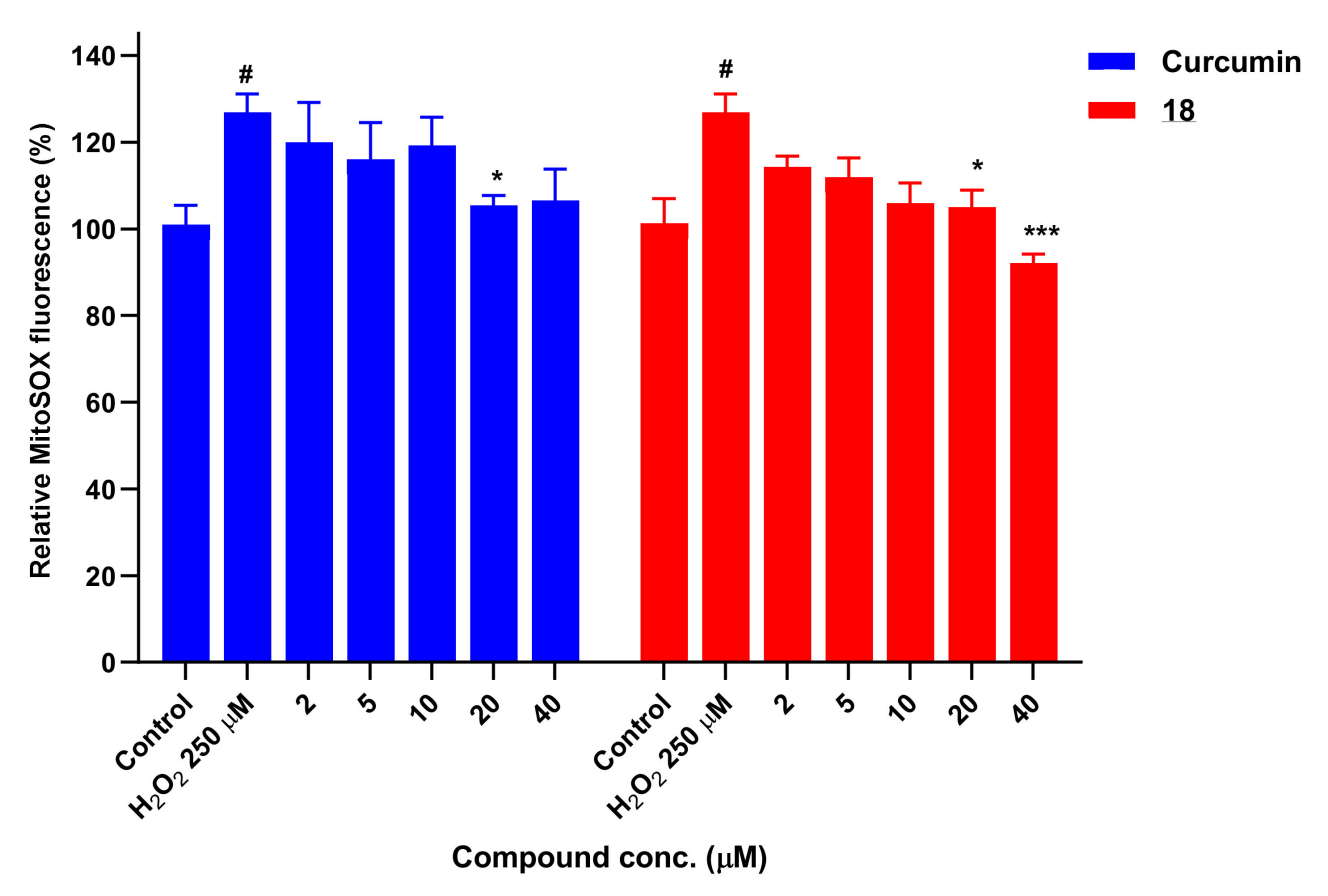
Reduction in mitochondrial superoxide levels by 18 and curcumin in SH-SY5Y. Cells were treated with **18** and curcumin for 24 h prior to exposure to H_2_O_2_. ROS levels were evaluated with MitoSox. Data are presented as the Mean ± SEM of three independent experiments carried out in quadruplicate. # *p* < 0.001 vs. vehicle-treated control. * *p* < 0.05, *** *p* < 0.001 vs. only with H_2_O_2_-treated cells. Figure S4 (Supplementary Materials) shows microscopy images from MitoSOX experiments performed with compound **18**.

To evaluate the capacity of compound **18** to reduce the mitochondrial superoxide levels of SH-SY5Y cells under oxidative stress conditions, experiments were performed with MitoSox, using curcumin as a control compound and H_2_O_2_ 250 µM as an oxidative stressor. Figure 19 shows that after 24-h treatment with compound **18**, it was able to reduce mitochondrial superoxide levels in a dose-dependent manner, being statistically significant at the highest concentrations assayed. Curcumin demonstrated only a clear reduction at 20 µM. These results are in agreement with those previously found regarding the reduction in intracellular ROS levels for compound **18**. On the other hand, our results for curcumin at 5 µM are in general agreement with those previously reported although under less demanding conditions [157] (H_2_O 100 µM).

### 3.11. Study of Apoptosis and Necrosis

#### 3.11.1. Evaluation of Caspase 3 Activity

Apoptosis and necrosis are two forms of cell death. Apoptosis depends on an intracellular proteolytic pathway mediated by two types of caspases, initiator caspases (caspase-8 and caspase-9) and executioner caspases (caspase-3, caspase-6, and caspase-7) [170]. Caspases are cysteine proteases that are involved in triggering two pathways of apoptosis: the intrinsic pathway, which relies on the release of cytochrome c and activation of caspase-3, and the extrinsic pathway, which depends on the activation of cell surface death receptors, generating the activation of caspase-8, which activates caspase-3, a key mediator of apoptosis in neuronal cells.

**Figure 20:**
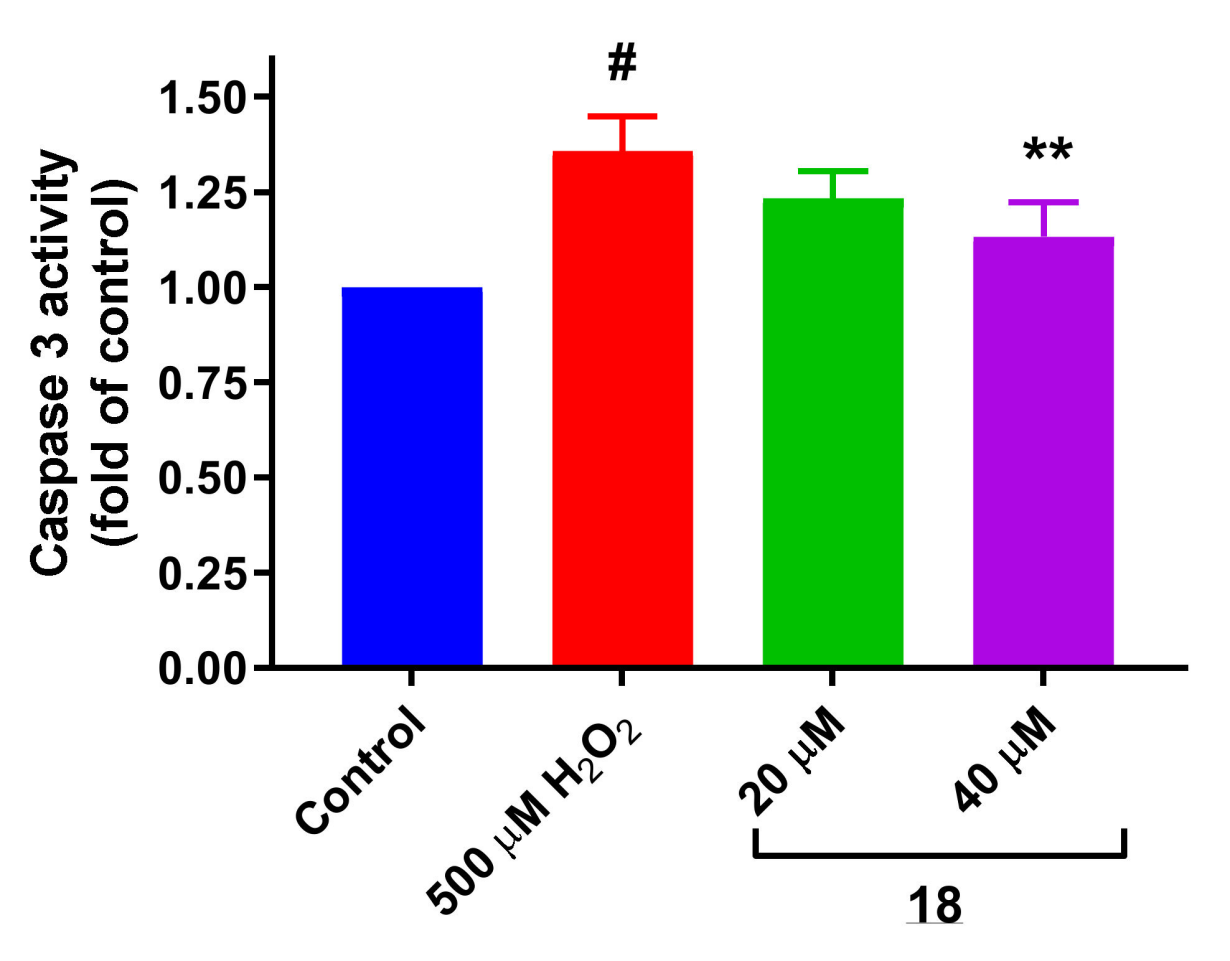
Reduction in caspase-3 activity by compound **18** in SH-SY5Y cells. Cells were treated with 18 at 0, 20, and 40 µM for 24 h at 37° and then with H_2_O_2_ 500 µM for 4 h. Data are presented as the Mean ± SEM of three different experiments carried out in quadruplicate. # *p* <0.001 vs. vehicle-treated control. ** *p* < 0.01 vs. only with H_2_O_2_-treated cells.

To understand the potential mechanism involved in the neuroprotectant properties of compound **18** in SH-SY5Y cells under oxidative stress pressure, the levels of cellular apoptosis were measured using caspase-3 as an apoptosis biomarker. In these experiments, cells were treated with compound **18** at 20 and 40 µM for 24 h at 37 °C and afterward with H_2_O_2_ 500 µM for 4 h. Caspase-3 activity was measured as a fold increase relative to the control. Results shown in Figure 20 demonstrate that a 24-h treatment with **18** reduced the levels of activated caspase 3 at the concentrations tested, being statistically significant at 20 µM. These results are in agreement with those previous results showing that the protectant effect of compound **18** reduces both the intracellular levels of ROS and superoxide radicals and also stimulates the recovery of the mitochondrial membrane potential. However, under the present conditions (H_2_O_2_ 500 µM for 4 h), curcumin did not show any reduction in caspase-3 activity at the concentrations tested (data not shown), although it was reported [157] that at 5 µM and under H_2_O_2_ 100 µM treatment, curcumin showed a caspase-3 activity decrease.

#### 3.11.2. Study of Apoptosis and Necrosis by Flow Cytometry with Annexin-V PE/7-AAD

To study whether compound **18** protects from apoptotic and necrotic cell death caused by H_2_O_2_, SH-SY5Y cells were stained with Annexin V conjugated with PE (Phycoerythrin) and 7-ADD (7-Aminoactinomycin) used to label apoptotic and necrotic cells, respectively. Annexin V (human anticoagulant protein) is a Ca^2+^-dependent phospholipid-binding protein with an affinity for phosphatidylserine (PS). PS is located on the inner side of the cell membrane in viable cells. Under oxidative stress cell damage, PS is translocated from the inner to the outer part of the plasma membrane in apoptotic cells, being accessible for binding to Annexin V fluorescent conjugates, which can then be analyzed by flow cytometry.

**Figure 21:**
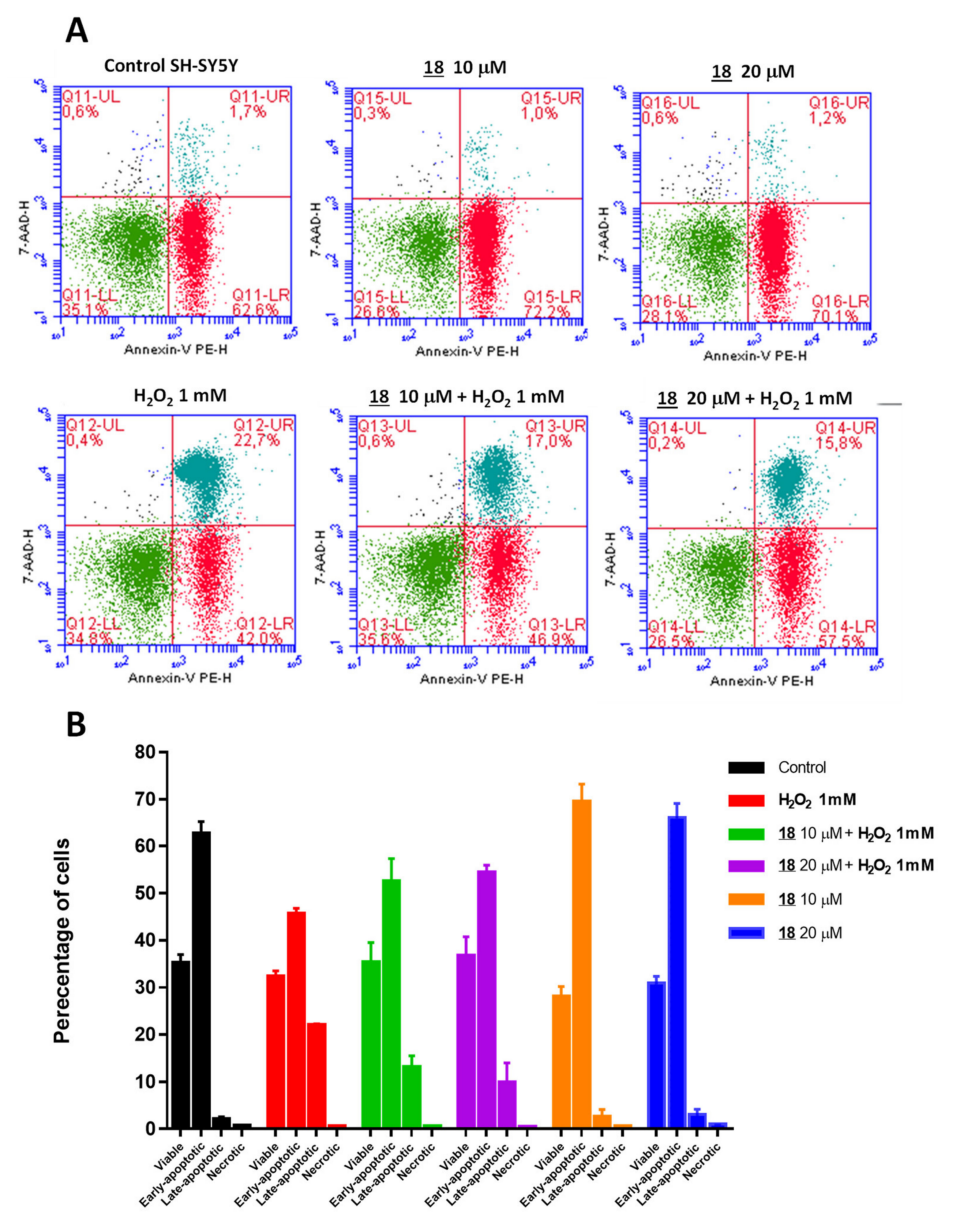
Results from apoptosis and necrosis flow cytometry experiments with Annexin V-PE and 7-AAD. (**A**) Dot plots for each compound treatment where quadrants indicate the cell population’s distribution for each sample. SH-SY5Y cells were treated with **18** at 0, 10, and 20 µM for 24 h at 37° and then exposed to H_2_O_2_ 1 mM for 4 h. Q1-LL: viable cells; Q1-LR: early apoptotic cells; Q1-UR: late apoptotic cells; Q1-UL: necrotic cells. (**B**) Histogram representation of the percentage of each cell population for each compound treatment. Data are the Mean ± SD of four independent experiments.

In these experiments, SH-SY5Y cells were treated for 24 h at 37 °C with compound **18** at 0, 10, and 20 µM and then exposed to H_2_O_2_ 1 mM for 4 h. Figure 21A shows the results obtained as flow cytometric dot plots of one representative experiment. Results are shown as fluorescence intensity on the x-axis (Annexin V-PE) vs. y-axis (7-AAD) in six flow cytometry plots representative of each compound treatment. The four quadrants in each plot represent viable cells (Q1-LL: AV-/PI-), early apoptotic cells (Q1-LR: AV+/PI-), late apoptotic cells (Q1-UR: AV+/PI+), and necrotic cells (Q1-UL: AV-/PI+). Values represent the percentage of cells in each quadrant for 15,000 total events recorded. Figure 21B shows the percentage of each cell population for each compound treatment. From these results, it can be highlighted that in comparison with the H_2_O_2_- only-treated cells, compound **18** in an acute model after 4 h exposure to H_2_O_2_ 1 mM at 10 and 20 µM reduced the number of late apoptotic cells, increasing in parallel the number of early apoptotic and viable cells.

### 3.12. Determination of Lipid Peroxidation Levels

**Figure 22:**
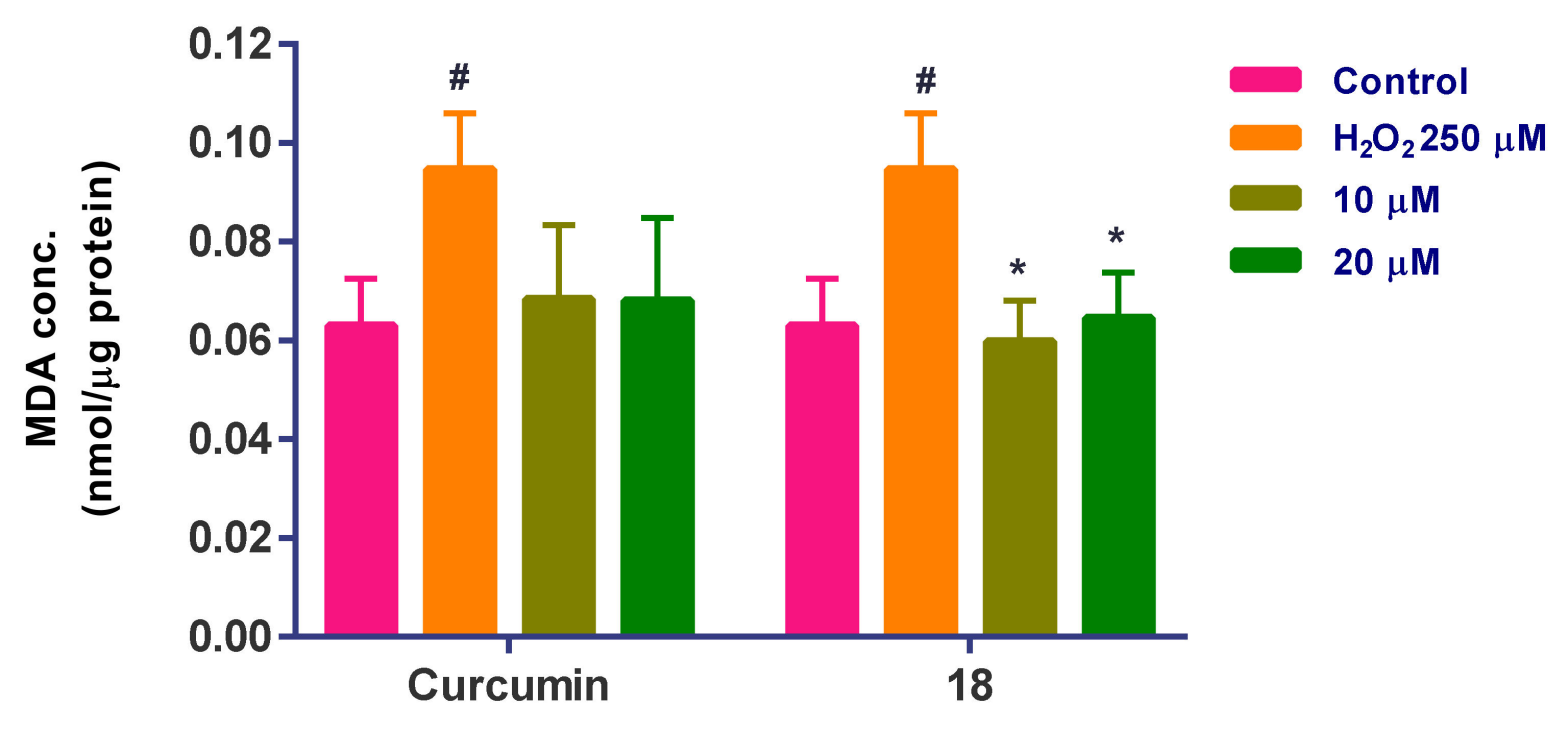
Effect of compound **18** and curcumin on MDA levels in SH-SY5Y cells exposed for 24 h to oxidative stress pressure with 250 µM H_2_O_2_. Cells were treated with derivative 18 or curcumin at 10 and 20 µM for 24 h prior to exposure to H_2_O_2_. Data are presented as the Mean ± SD of four independent experiments carried out in triplicate. # *p* < 0.001 vs. vehicle-treated control. * *p* < 0.05 vs. cells treated only with H_2_O_2_.

Under oxidative stress conditions, ROS causes membrane lipid peroxidation and pathophysiology related to neurodegeneration [171,172]. Malondialdehyde (MDA) is a secondary product of lipid peroxidation that is used as a biomarker of oxidative stress [91,173], and it is measured by its reaction with thiobarbituric acid [91,174]. In this work, lipid peroxidation in SH-SY5Y cells was determined by the level of MDA generated under oxidative stress conditions using the TBARS method [91]. Figure 22 shows that 250 µM H_2_O_2_ stimulated the production of MDA significantly, while this increase was significantly prevented by treatment with derivative **18** at 10 and 20 µM (*p* <0.05) in comparison with those results obtained for curcumin. In the case of curcumin, the values in Figure 22 show a reduction of MDA levels although without statistical significance, which is consistent with those results from Ugûz et al. [157], under H_2_O_2_ 100 µM treatment.

### 3.13. Determination of the GSH/GSSG Ratio

Among the endogenous antioxidant defenses, glutathione (GSH) is the principal intracellular small molecular thiol that has a critical role in the cellular defense against oxidative stress insults [175]. In its reduced form, it protects cells from oxidative injury and maintains vital homeostatic functions. Altered GSH concentration and increases in oxidative stress compromise cellular defenses against ROS and are associated with neurodegenerative and aging-related diseases [176,177].

The formation of GSSG from the oxidation of two molecules of GSH is the result of the reaction catalyzed by Glutathione Peroxidase (GPX). Regeneration of GSH from GSSG is the catalytic process performed by glutathione reductase (GR) through oxidation of β-nicotinamide adenine dinucleotide phosphate (β-NADPH2). Cells under oxidative stress pressure undergo a reduction in GSH levels in parallel with an increase in GSSG concentration, which ultimately reduces the GSH/GSSG ratio. Therefore, the determination of the GSH/GSSG ratio is a key oxidative stress biomarker in cells.

**Figure 23:**
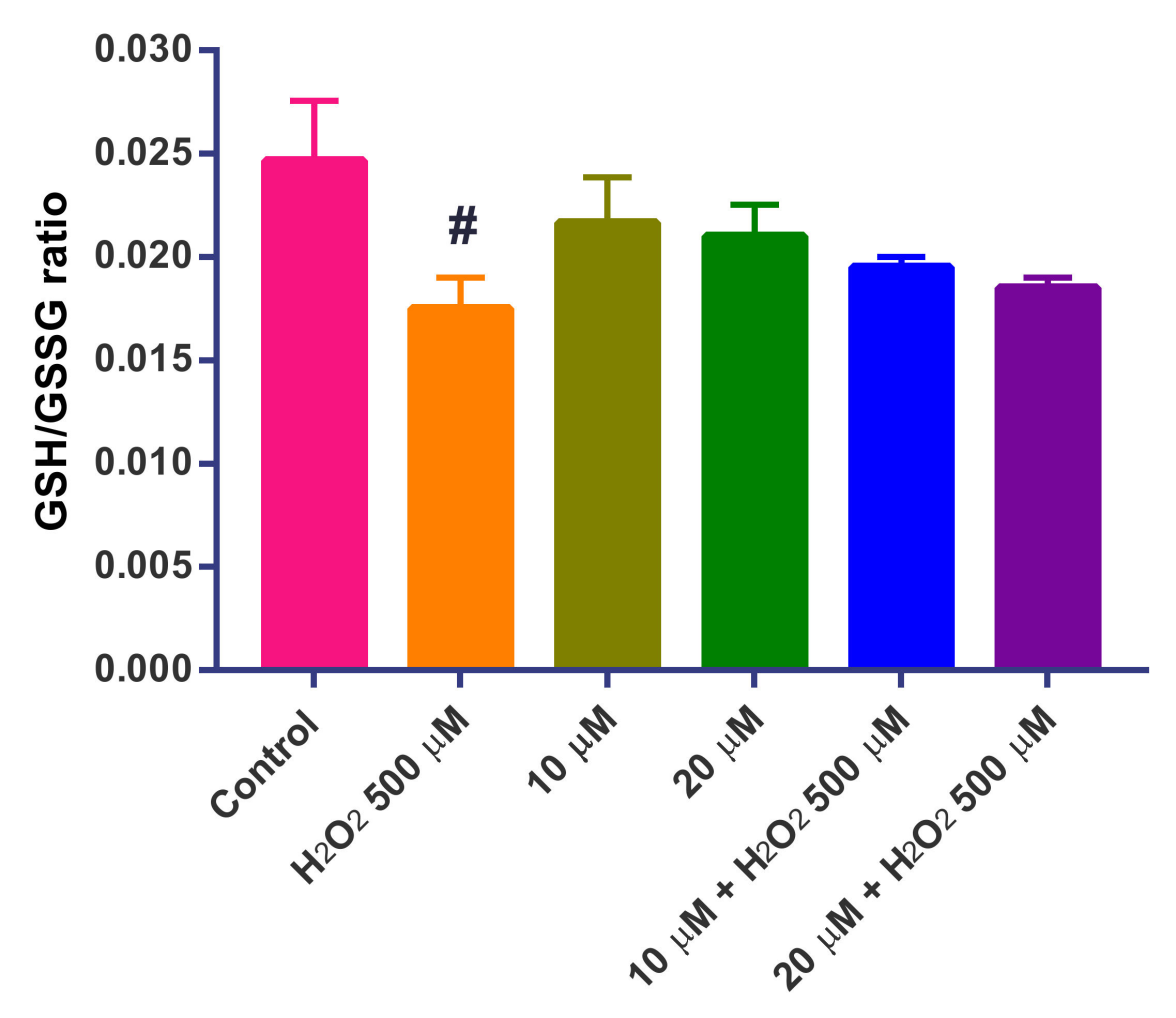
GSH/GSSG ratios determined for compound **18** at 10 and 20 µM in SH-SY5Y cells under H_2_O_2_ 500 µM oxidative stress. Data are presented as the Mean ± SD of four independent experiments carried out in triplicate. # *p* < 0.001 vs. vehicle-treated control.

In this work, the GSH/GSSG ratios were evaluated for SH-SY5Y cells treated for 24 h with compound **18** at 0, 10, and 20 µM and then exposed to H_2_O_2_ 500 µM for 6 h. Figure 23 shows that derivative **18** at 10 and 20 µM improved the GSH/GSSG ratios of cells under oxidative stress, although without statistical significance. From these data, we can conclude that treatment with compound **18** at 20 and 40 µM yielded GSH/GSSH ratios similar to those of control cells without H_2_O_2_. In parallel, cells treated with **18** and exposed to H_2_O_2_ 500 µM showed better GSH/GSSG ratios that those exposed only to H_2_O_2_.

### 3.14. Determination of Intracellular GSH Levels

**Figure 24:**
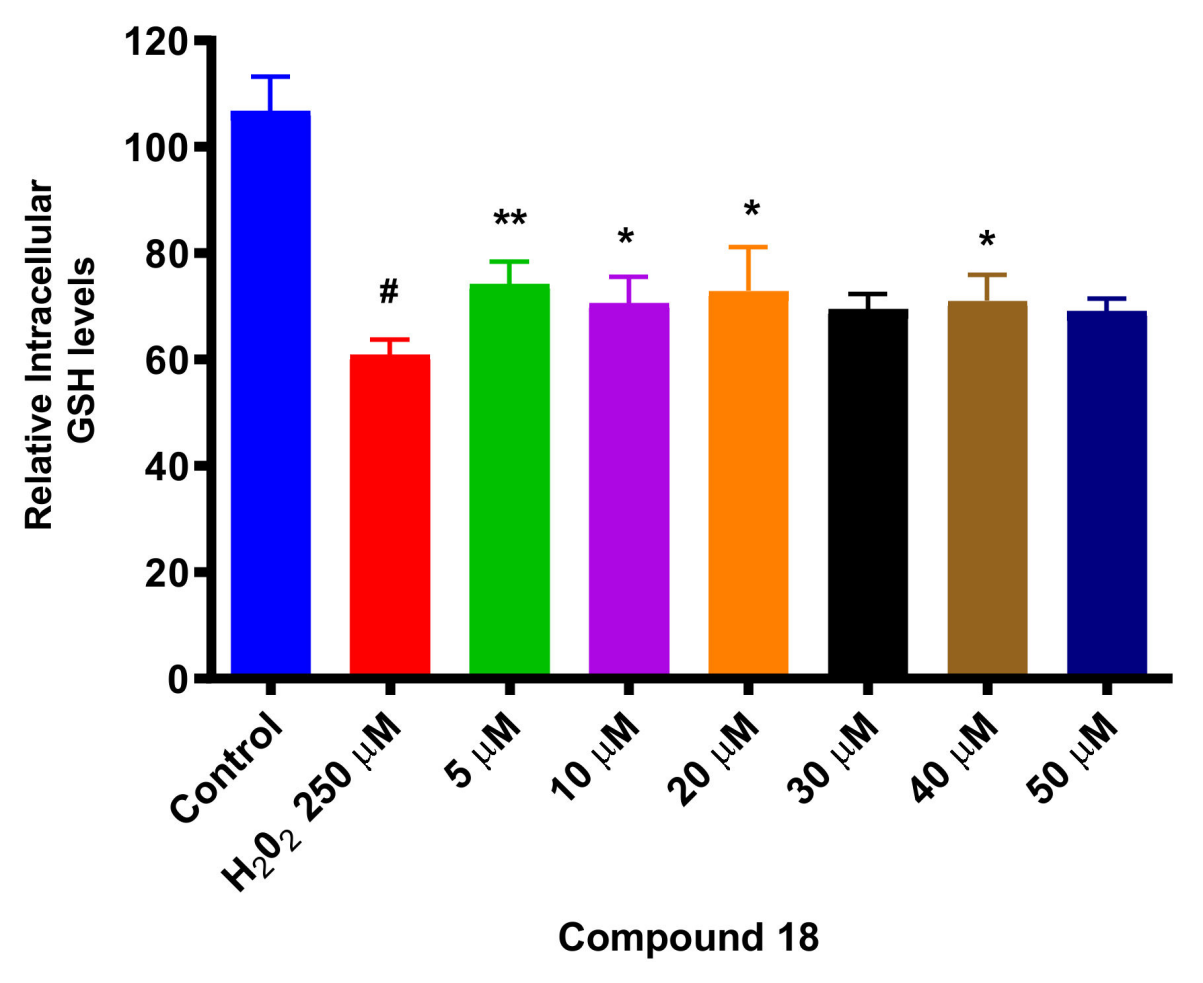
Effect of compound **18** on intracellular GSH levels in SH-SY5Y cells exposed for 24 h to oxidative stress pressure with 250 µM H_2_O_2_. Cells were treated with derivative 18 at 5, 10, 20, 30, 40, and 50 µM for 24 h prior to exposure to H_2_O_2_. Data are presented as the Mean ± SD of four independent experiments carried out in triplicate. # *p* < 0.001 vs. vehicle-treated control. * *p* < 0.05, ** *p* < 0.01 vs. cells treated only with H_2_O_2_.

To analyze intracellular GSH in cultured cells, a well-known method is the addition of monochlorobimane (mClB) to the culture medium where it enters cells to form a fluorescent GSH-chlorobimane adduct that can be measured fluorometrically in a reaction catalyzed by glutathione S-transferase (GST) [94,178]. In the present work, this method was used for intracellular GSH evaluation after treatment with compound **18**. Figure 24 shows that a 24-h H_2_O_2_ 250 µM insult reduced the intracellular GSH levels by 40%, and that after treatment with derivative **18**, there was a significant GSH recovery at 5 µM (*p* <0.01), 10 µM (*p* <0.05), and 20 µM (*p* <0.05) of about 18–20%. However, these results did not show a dose–response effect in the recovery of intracellular GSH levels.

### 3.15. Neuroprotection Assays in SH-SY5Y Differentiated Cells (diffSH-SY5Y)

To verify the real potential as a neuroprotectant of this chemotype, experiments with SH-SY5Y differentiated cells were performed. It has been well documented that the undifferentiated neuroblastoma SH-SY5Y cell line is a good in vitro model of dopaminergic neurons for the study of neurodegeneration pathogenesis and drug screening [140], although it maintains a fast proliferative profile because it expresses specific dopaminergic markers such as tyrosine hydroxylase (TH) and the dopamine transporter (DAT) [178]. On this basis, it is reasonable to evaluate these compounds in a more neuronal-like phenotype, such as the one developed by differentiated SH-SY5Y, with morphological changes and neurite-like processes with a more catecholaminergic phenotype [179,180,181]. There is a consensus on differentiation methods of SH-SY5Y cells that the best conditions are those that use all-trans-retinoic acid (RA) 10 µM for 6–14 days with serum starvation, alone or in combination with other reagents. In our case, the best results obtained were with all-trans-RA 10 µM in DMEM:F12 medium with 1% FBS for eight days. Figure S5 (Supplementary Materials) shows the different cell morphology from undifferentiated to differentiated SH-SY5Y.

To corroborate the neuronal-like profile of diffSH-SY5Y cells, experiments were performed with βIII-Tubulin and MAP2 antibodies as the most commonly used immunocytochemistry markers of neuronal differentiation (Figure S6A,B Supplementary Materials). Images from these figures underscore morphological changes of differentiated cells in comparison with undifferentiated ones, where diffSH-SY5Y cells had more extensive processes and dendrites highlighted by βIII-Tubulin and MAP2. Results from staining experiments with Phalloidin as a marker of actin filaments and DAPI for nuclei are shown in Figure S7 (Supplementary Materials).

**Figure 25:**
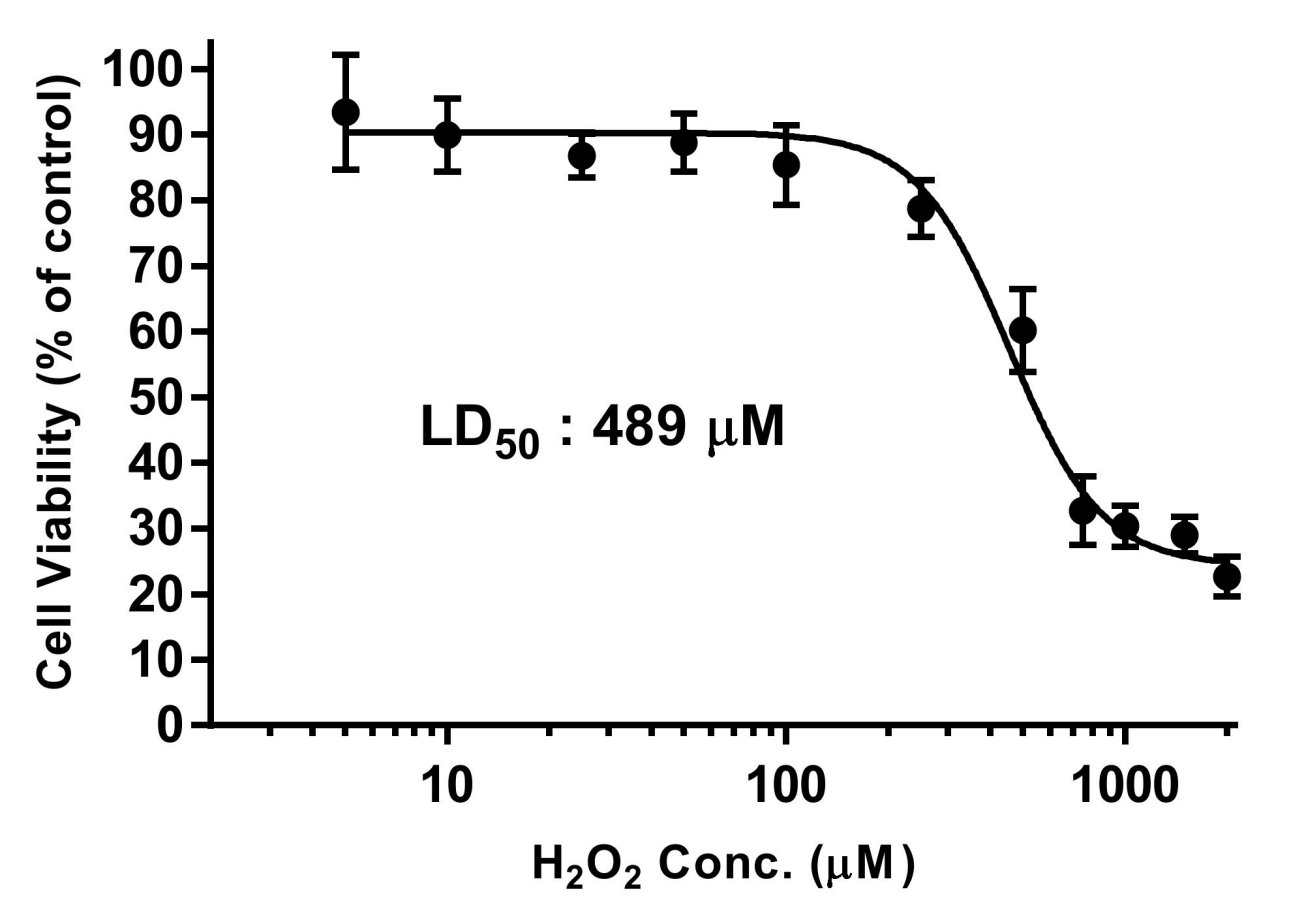
Determination of the H_2_O_2_ lethal dose (LD50, µM) after 24-h incubation of diffSH-SY5Y cells. Values are the Mean ± SD of four different experiments performed in quadruplicate (r2: 0.9801).

To evaluate the neuroprotectant activity of compound **18** against diffSH-SY5Y cells under oxidative stress pressure induced by H_2_O_2_, killing curves were constructed to determine the H_2_O_2_ concentration needed to achieve 50% cell viability after eight days of differentiation. The value of LD50 = 494 µM obtained from the curve shown in Figure 25 was similar to that reported in the literature [182]. Therefore, H_2_O_2_ 500 µM was used as a stressor in the following neuroprotection assays.

**Figure 26:**
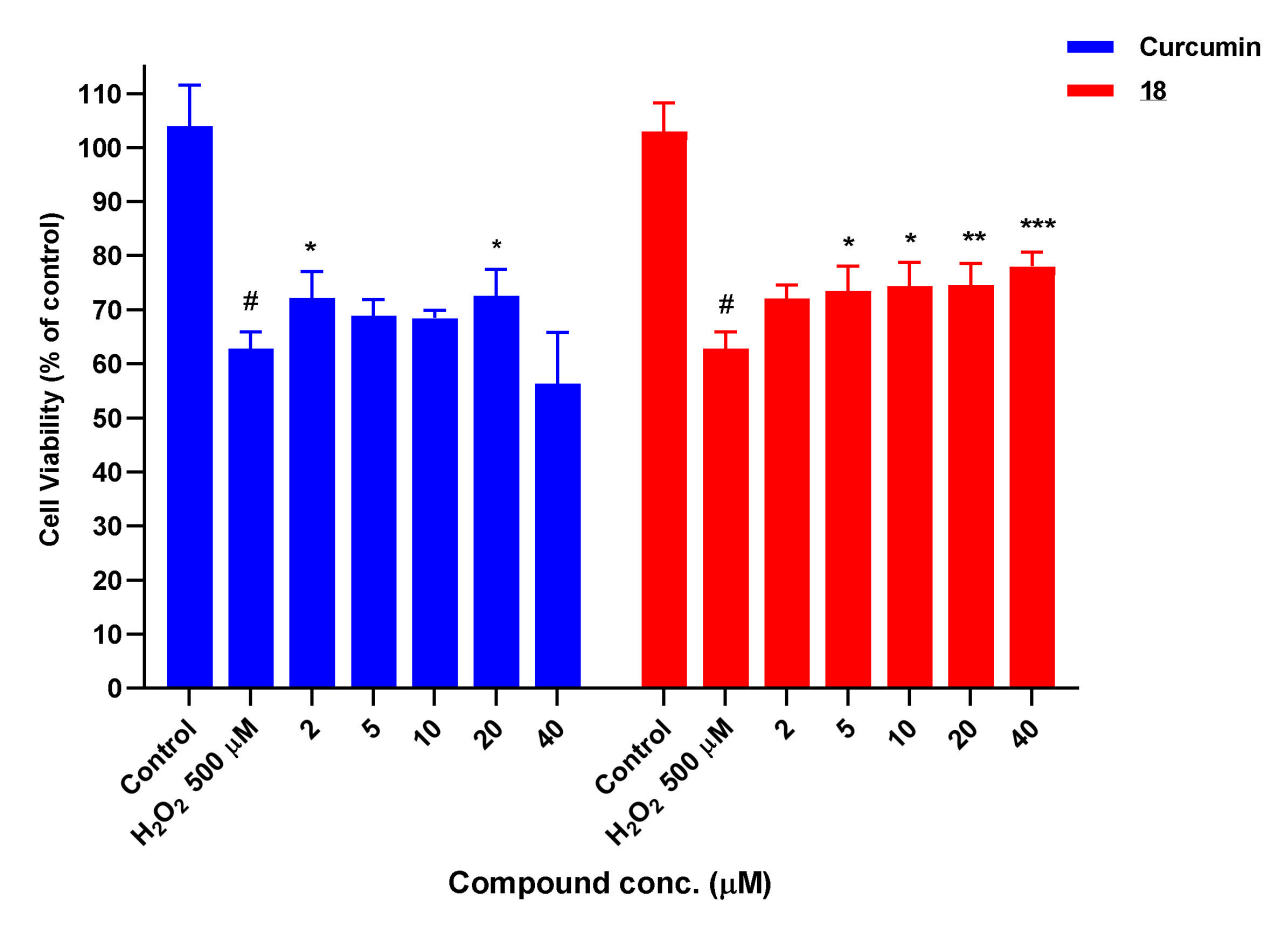
SH-SY5Y cells were differentiated by RA treatment (10 µM) and 1% FBS DMEM: F12 medium on days 2, 4, and 6 before antioxidant treatment on day 8. Cells were treated with curcumin or **18** for 24 h at 0, 2, 5, 10, 20, and 40 µM. Afterward, cells were exposed to H_2_O_2_ 500 µM for 24 h. Cell viability was measured by the MTT assay. Data are presented as the Mean ± SEM of four independent experiments carried out in triplicate. # *p* < 0.001 vs. vehicle-treated control. * *p* < 0.05, ** *p* < 0.01, *** *p* < 0.001 vs. cells treated only with H_2_O_2_.

Figure 26 shows the diffSH-SY5Y cell viability results obtained after treatment with compound **18** and curcumin at 0, 2, 5, 10, 20, and 40 µM under an oxidative stress pressure of H_2_O_2_ 500 µM with the MTT assay. It can be concluded that compound **18** yielded dose-dependent neuroprotectant activity in neuronal-like diffSH-SY5Y cells, with statistical significance at higher doses, while curcumin showed a more irregular activity profile with some toxicity at 40 µM.

## 4. Conclusions

In the present work, we report the synthesis of a series of twenty-three new 1,5-benzodiazepin-2(3H)-ones, which showed interesting antioxidant properties in the ABTS and FRAP assays with good drug-like physicochemical properties in comparison with curcumin. The most interesting derivatives did not show reactivity with H_2_O_2_ or MTT. Most of them also showed a lack of cytotoxicity against the human SH-SY5Y and HepG2 cell lines. In contrast to one well-known issue of curcumin, some compounds had good solubility in biorelevant media. As expected, only those compounds with the best AOX properties showed neuroprotection under H_2_O_2_-induced oxidative stress in the MTT and the LDH assays. 4-Phenyl-1H-1,5- benzodiazepin-2(3H)-one (**18**) and 4-(3,4,5-trimethoxyphenyl)-1H-1,5-benzodiazepin- 2(3H)-one (**20**) with the best AOX and cytotoxicity, solubility, and neuroprotection profiles were studied in the in vitro PD models of 6-OHD and MPP^+^ where they also demonstrated neuroprotection after 48 h under the pressure of those oxidative insults. In addition to that, both derivatives showed that they protect neuronal SH-SY5Y cells under oxidative stress conditions, with a significant reduction in the intracellular ROS and mitochondrial superoxide levels, and with a substantial recovery of the mitochondrial membrane potential.

**Figure 27:**
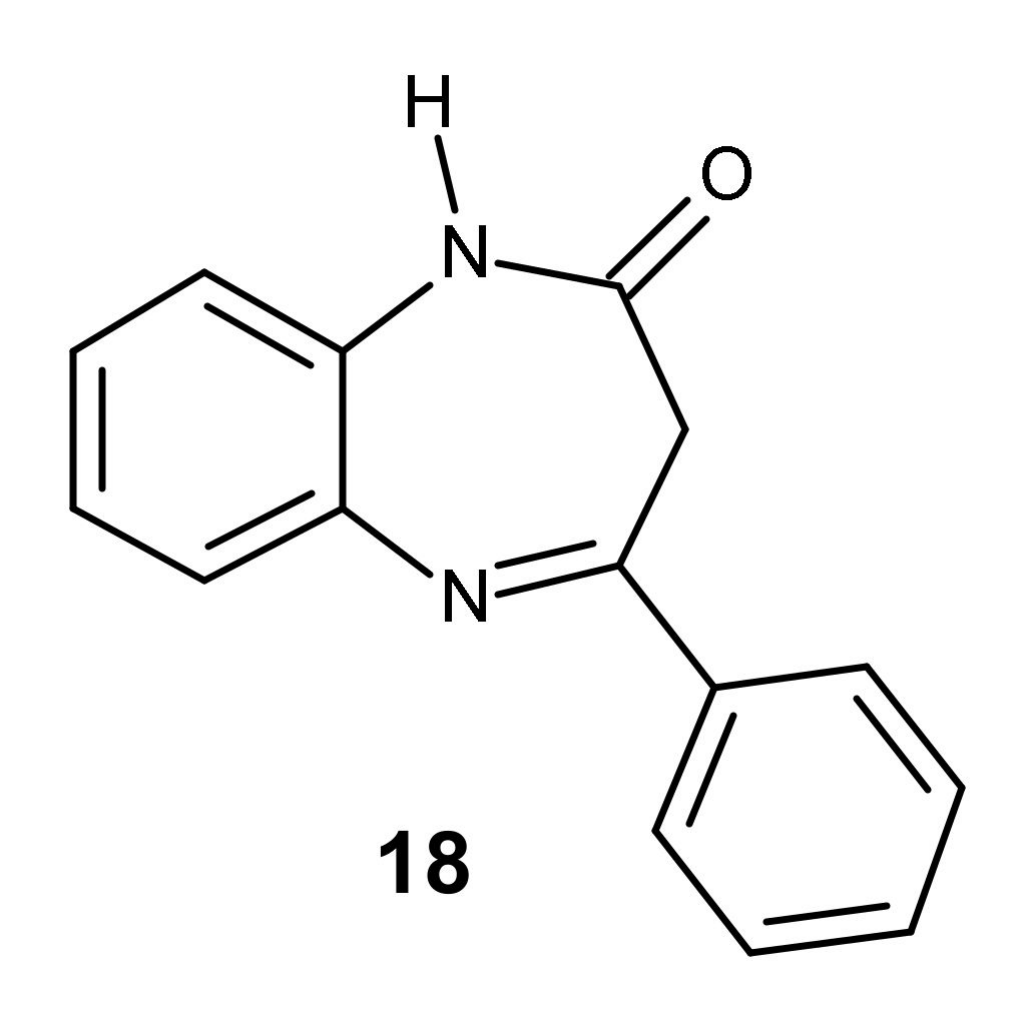
Compound **18** with the best neuroprotectant profile.

In comparison with curcumin, compound **18** (Figure 27) showed a better reduction of lipid peroxidation levels as well as a better intracellular GSH recovery in the SH-SY5Y cells under H_2_O_2_-induced oxidative stress. There was a reduction in apoptosis and caspase-3 levels. Finally, the neuroprotectant activity shown in neuron-like differentiated SH-SY5Y cells under H_2_O_2_ oxidative stress pressure is noteworthy. In summary, this new family of 1,5-benzodiazepin-2(3H)- ones with drug-like properties is a good starting point for further chemical exploration, as well as for studies that allow the elucidation of their mechanism of action and therapeutic target. Compound **18** should be used in in vitro and in vivo studies where it can demonstrate its potential as a new therapeutic agent, not only for Parkinson’s Disease, but also for other neurodegenerative pathologies where oxidative stress may be involved.

## Figures and Tables

**Table 1:** Antioxidant properties of benzodiazepinones.

		Antioxidant Capacity (%)				
	ABTS Assay			FRAP Assay		
Compound	IC_50_ (μM)	TEAC_50_ *	TEAC_100_ *	at 100 uM	at 200 uM	Trolox Equivalents
**Trolox**	25.1 ± 1.2	1	1	51.3	100	1
**Curcumin**	21.0 ± 1.5	1.1	1.1	49.0	74.4	0.96
**1**	576.3 ± 40.0	0.33	0.16	0.5	0.5	0.01
**2**	>1000	n.d.	n.d.	1.00	0.5	0.02
**3**	>1000	n.d.	n.d.	1.5	1.8	0.03
**4**	>1000	n.d.	n.d.	1.1	1.3	0.02
**5**	658.4 ± 96.0	0.32	0.15	1.4	5.5	0.03
**6**	>1000	n.d.	n.d.	1.1	1.6	0.02
**7**	635.0 ± 87.0	0.35	0.16	0.6	0.4	0.01
**8**	>1000	n.d.	n.d.	0.6	0.2	0.01
**9**	431.6 ± 68.0	0.36	0.18	21.9	37.1	0.43
**10**	>1000	n.d.	n.d.	0.3	0.6	0.01
**11**	306.4 ± 34.6	0.35	0.17	28.9	49.8	0.56
**12**	>1000	n.d.	n.d.	0.8	1.4	0.02
**13**	324.7 ± 55.0	0.36	0.19	40.7	61.6	0.79
**14**	>1000	n.d.	n.d.	0.3	0.6	0.01
**15**	495.5 ± 82.0	0.34	0.15	3.8	6.2	0.07
**16**	>1000	n.d.	n.d.	0	0	0.00
**17**	>1000	n.d.	n.d.	1.8	2.5	0.04
**18**	187.3 ± 15.2	0.36	0.21	45.7	65.1	0.89
**19**	>1000	n.d.	n.d.	1.7	3.5	0.03
**20**	269.0 ± 27.2	0.34	0.18	48.1	63.8	0.90
**21**	137.0 ± 5.3	0.36	0.21	47.2	60.9	0.89
**22**	>500	n.d.	n.d.	15.7	13.7	0.30
**23**	>500	n.d.	n.d.	2.7	4.6	0.03
**24**	64.2 ± 4.4	0.58	0.32	6.0	10.9	0.11

* TEAC50 y TEAC100: Equivalent antioxidant capacity at Trolox 50 mM and 100 mM. n.d.: not determined.

**Table 2:** Calculated physicochemical properties of 1,5-Benzodiazepin-2(3*H*)-ones *.

Compound	MW	clogP	logD	HBD	NO	TPSA	BBB Permeant
**Curcumin**	368.39	2.256	2.778	3	6	96.2	No
**1**	246.165	2.311	1.107	1	3	41.46	Yes
**2**	260.192	2.583	1.362	0	3	32.67	Yes
**3**	308.237	3.355	2.646	1	3	41.46	Yes
**4**	322.264	3.593	2.854	0	3	32.67	Yes
**5**	326.227	3.475	2.705	1	3	41.46	Yes
**6**	340.254	3.71	2.918	0	3	32.67	Yes
**7**	342.682	3.593	3.101	1	3	41.46	Yes
**8**	356.709	3.825	3.297	0	3	32.67	Yes
**9**	272.256	3.112	2.424	1	3	41.46	Yes
**10**	286.283	3.355	2.528	0	3	32.67	Yes
**11**	254.265	2.988	2.334	1	3	41.46	Yes
**12**	268.292	3.234	2.434	0	3	32.67	Yes
**13**	254.265	2.988	2.207	1	3	41.46	Yes
**14**	268.292	3.234	2.296	0	3	32.67	Yes
**15**	272.256	3.112	2.334	1	3	41.46	Yes
**16**	286.283	3.355	2.484	0	3	32.67	Yes
**17**	272.256	3.112	2.483	1	3	41.46	Yes
**18**	236.275	2.595	2.081	1	3	41.46	Yes
**19**	250.302	2.844	2.156	0	3	32.67	Yes
**20**	326.354	1.225	1.799	1	6	69.15	Yes
**21**	362.335	1.458	2.121	1	6	69.15	Yes
**22**	354.408	1.685	2.436	1	6	69.15	Yes
**23**	395.244	1.685	3.014	1	6	69.15	Yes

* Calculations were performed with MedChem Designer. clogP: Moriguchi LogP; logD: Distribution coefficient at pH 7.4; NO: Number of Oxygen atoms; TPSA:Total Polar Surface Area; HBD: number of Hydrogen Bonding Donor groups.

**Table 3:** Solubility and melting points of 1,5-Benzodiazepin-2(3*H*)-ones.

Compound	Solubility (µM) *	mp °C	Silicos-IT Class **
**Curcumin**	<50	ND	Moderately soluble
**1**	>200	155.2	Moderately soluble
**2**	>200	132.2	Moderately soluble
**3**	<100	253.3	Poorly soluble
**4**	ND	133.7	Poorly soluble
**5**	<20	248.1	Poorly soluble
**6**	<200	142.6	Poorly soluble
**7**	<50	211.7	Poorly soluble
**8**	<100	128.7	Poorly soluble
**9**	<50	214.2	Poorly soluble
**10**	>200	115.8	Moderately soluble
**11**	<20	212.5	Poorly soluble
**12**	>200	oil	Moderately soluble
**13**	<20	205.7	Poorly soluble
**14**	>200	107.7	Moderately soluble
**15**	<10	213.4	Poorly soluble
**16**	>200	60.2	Moderately soluble
**17**	<50	221.5	Poorly soluble
**18**	<100	208.2	Moderately soluble
**19**	>200	oil	Moderately soluble
**20**	>200	215.4	Poorly soluble
**21**	<100	208.5	Poorly soluble
**22**	<100	241.8	Poorly soluble
**23**	<20	246.0	Poorly soluble

* Solubility in PBS 50 mM. ** Predicted solibility [113].

**Table 4:** Cytotoxicity of 1,5-Benzodiazepin-2(3*H*)-ones.

Compound	SH-SY5Y IC_50_ (µM) *	HepG2 IC_50_ (µM) *
**Curcumin**	48.8 ± 5.1	109.7 ± 9.8
**1**	>250	>250
**2**	>250	>250
**3**	>250	>250
**4**	>250	>250
**5**	>250	>250
**6**	>250	>250
**7**	135.0 ± 11.2	201 ± 13.1
**8**	150.1 ± 17.0	206.8 ± 27.0
**9**	>250	>250
**10**	193 ± 15.9	>250
**11**	>250	>250
**12**	240.1 ± 24.9	230.2 ± 25.5
**13**	>250	>250
**14**	220.2 ± 27.9	233.0 ± 18.7
**15**	>250	>250
**16**	>250	>250
**17**	>250	>250
**18**	>250	223.7 ± 35.0
**19**	200.6 ± 27.0	>250
**20**	>250	>250
**21**	>250	>250
**22**	>250	>250
**23**	>250	N.D.

* Values are the mean ± SD of four different experiments performed by cuadruplicate. N.D.: Not determined due to solubility issues.

**Table 5:** Neuroprotection activity in SH-SY5Y cells under oxidative stress conditions (H_2_O_2_ 250 µM).

	% Cell Viability at Compound Concentration *			
Compound	5 µM	10 µM	20 µM	40 µM
**Curcumin**	+	+	−	−
**1**	−	−	−	−
**2**	−	−	−	−
**3**	−	−	−	−
**4**	−	−	−	−
**5**	−	−	−	−
**6**	−	−	−	−
**7**	+	−	−	−
**8**	−	−	−	−
**9**	+	+	−	−
**10**	−	−	−	−
**11**	+	+	+	−
**12**	−	−	−	−
**13**	−	+	+	−
**14**	−	−	−	−
**15**	−	−	−	−
**16**	−	−	−	−
**17**	−	−	−	−
**18**	+	+	+	+
**19**	−	−	−	−
**20**	+	+	+	+
**21**	+	−	−	−
**22**	−	−	−	−
**23**	−	−	−	−

* Assays were carried out twice by cuadruplicate.

## Data Availability

Data are contained within the article and Supplementary Materials.

## Supplementary Materials

The following are available online at www.mdpi.com/article/10.3390/antiox10101584/s1. Figure S1. Cytotoxicity results were obtained for curcumin (**A**) and compound **18** (**B**) against SH-SY5Y cells. Images were taken with a Leica DM IL inverted microscope with a Leica EC3 camera at 10×. Figure S2. Results from protection experiment in SH-SY5Y cells with curcumin (**A**) and compound 18 (**B**) under oxidative stress induced by H2O2 250 µM. Images were taken with a Leica DM IL inverted microscope with a Leica EC3 camera at 10×. Figure S3. Representative fluorescent microscopy images from Calcein-AM staining experiments with compound 18 against H2O2 induced cytotoxicity in SH-SY5Y cells. Images were taken at 4×. Figure S4. Images from MitoSOX experiments performed with compound 18 at 10, 20 and 40 µM in SH-SY5Y cells under oxidative stress induced by H2O2 250 µM for 24 h. Images were taken with a Leica DM 5500B microscope with a Leica DFC425FX camera. Figure S5. Undifferentiated (**A**) versus differentiated (**B**) SH-SY5Y cells. SHSY5Y cells were differentiated for 8 days by RA 10 µM treatment in DMEM:F12 with FBS 1%. Medium with RA was replaced on Days 2, 4, 6, and 8. Pictures taken on day 8 allows the identification of a more neuronal-like morphology with axon-like projections. Images were taken at 10× magnification using a Leica DM IL inverted microscope with a Leica EC3 camera. Figure S6. Fluorescence microscopy images of undifferentiated and differentiated SH-SY5Y cells using βIII-Tubulin and MAP2 to highlight their different morphology features. Images were obtained at 50× magnification. Figure S7. Staining of undifferentiated and differentiated SH-SY5Y cells with phalloidin and DAPI. Images were taken at 50 µm with a Leica DM5500 microscope.
