# Supplementary material for: 1,5-Benzodiazepin-2(3H)-ones: In Vitro Evaluation as Antiparkinsonian Agents
Source: Antioxidants (Basel). 2021 Oct 9;10(10):1584. doi: 10.3390/antiox10101584 (PMC8533176; doi:10.3390/antiox10101584)
Supplement: Supplementary file 1 [file antioxidants-10-01584-s001.zip › antioxidants-1237612-supplementary.pdf]

SUPPLEMENTARY INFORMATION

1,5-Benzodiazepin-2(3H)-ones: *in vitro* evaluation as antiparkinsonian agents

Ana Ortíz de Zárate[1], Marta Pérez-Torralba[2], Iñigo Bonet Isidro[1], Concepción López[2], Rosa M. Claramunt[2,*] Diana Martínez-Casanova[1], Isabel Sánchez-Vera[1], Jesús Jiménez González[1] and José Luis Lavandera[1,3,*]

[1]Instituto de Medicina Molecular Aplicada (IMMA), Facultad de Medicina, Universidad San Pablo-CEU. CEU Universities. Campus de Montepríncipe, Boadilla, E-28668 Madrid, Spain.
[2]Departamento de Química Orgánica y Bio-Orgánica, Facultad de Ciencias, Universidad Nacional de Educación a Distancia (UNED), Paseo Senda del Rey, 9, E-28040 Madrid, Spain.
[3] Department of Pharmacology, Physiology and Neuroscience. Medical School. Rutgers. The State University of New Jersey. 185 South Orange Avenue. Newark. NJ 07103. USA
*Correspondence: rclaramunt@ccia.uned.es; joseluis.lavandera@ceu.es

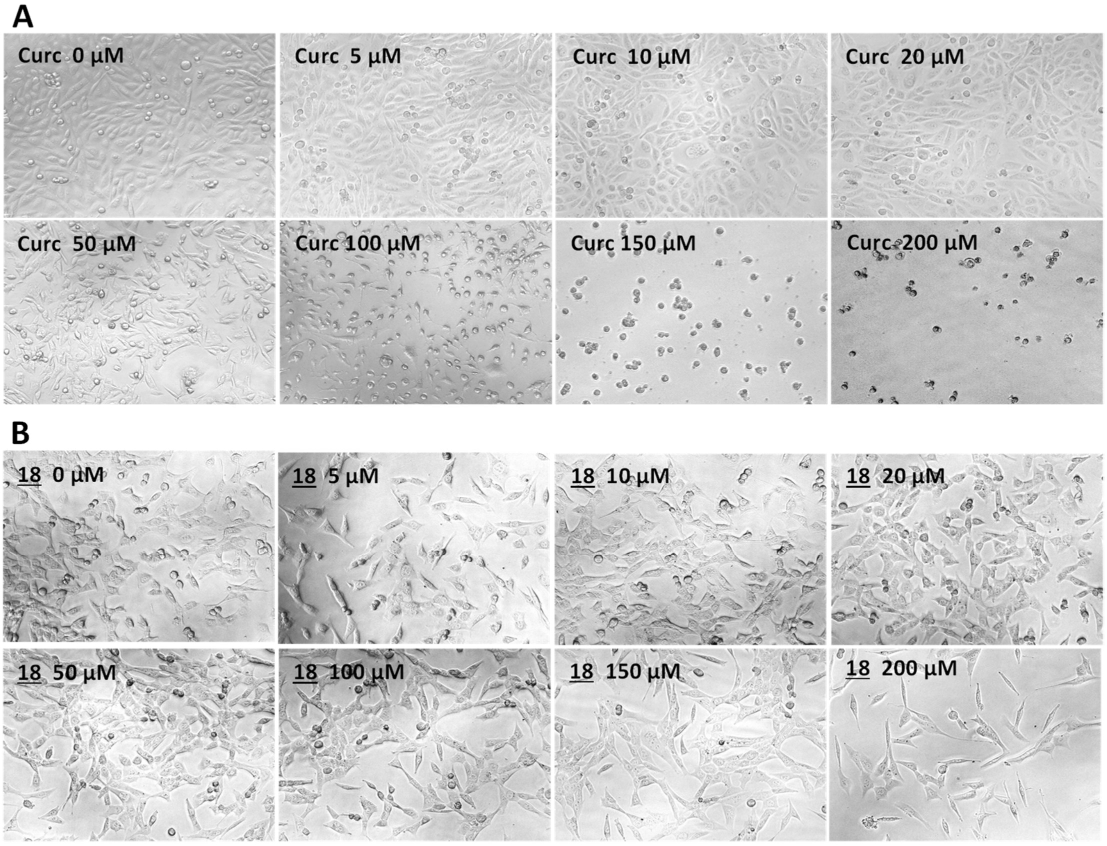

Figure S1. Cytotoxicity results were obtained for curcumin (A) and compound **18** (B) against SH-SY5Y cells. Images were taken with a Leica DM IL inverted microscope with a Leica EC3 camera at 10X.

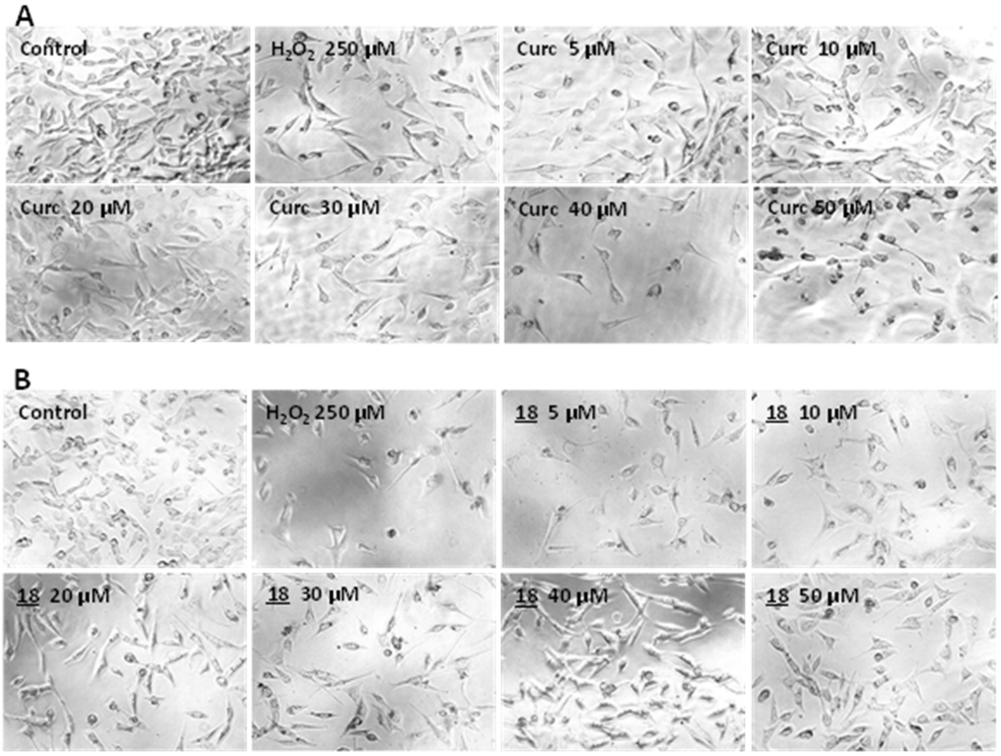

Figure S2. Results from protection experiment in SH-SY5Y cells with curcumin (A) and compound **18** (B) under oxidative stress induced by H_2O_2 250 µM. Images were taken with a Leica DM IL inverted microscope with a Leica EC3 camera at 10X.

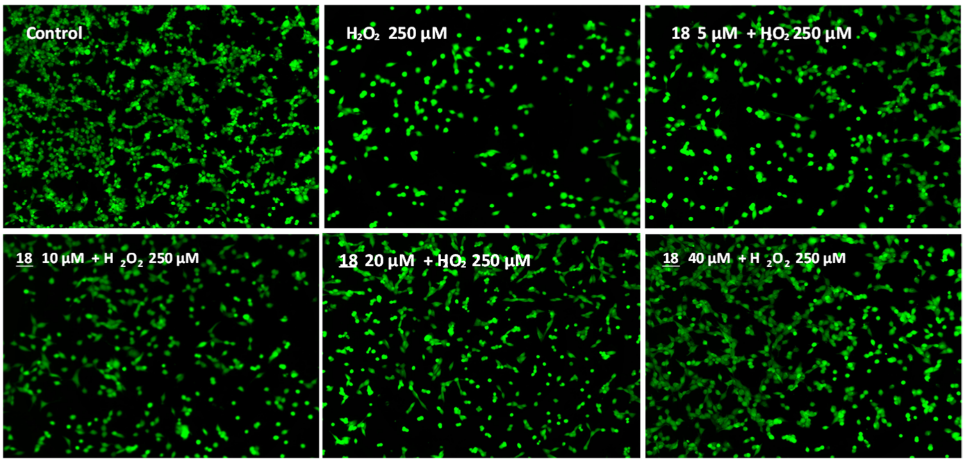

Figure S3. Representative fluorescent microscopy images from Calcein-AM staining experiments with compound 18 against H_2O_2 induced cytotoxicity in SH-SY5Y cells. Images were taken at 4X.

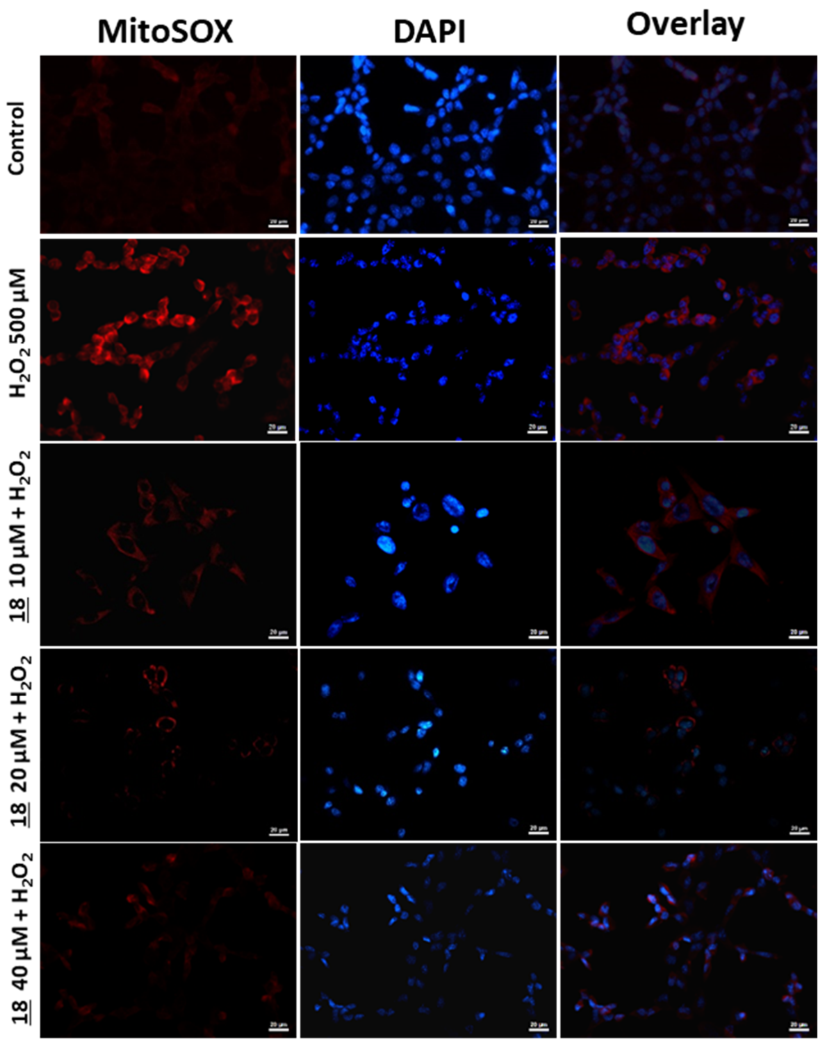

Figura S4. Images from MitoSOX experiments performed with compound 18 at 10, 20 and 40 µM in SH-SY5Y cells under oxidative stress induced by H_2O_2 250 µM for 24h. Images were taken with a Leica DM 5500B microscope with a Leica DFC425FX camera.

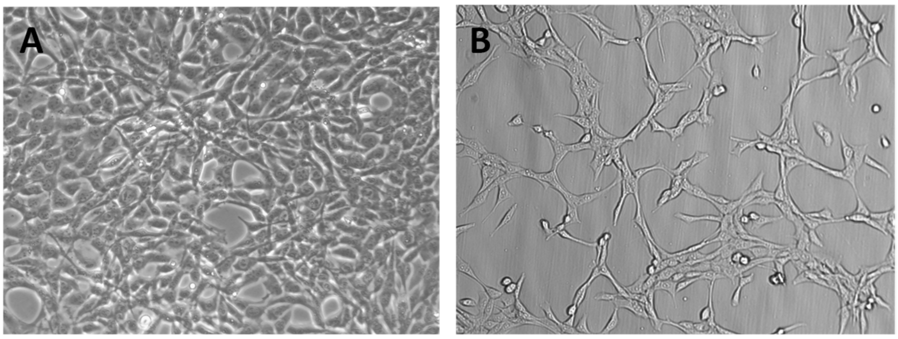

Figure S5. Undifferentiated (A) versus differentiated (B) SH-SY5Y cells. SHSY5Y cells were differentiated for 8 days by RA 10 μM treatment in DMEM:F12 with FBS 1%. Medium with RA was replaced on Days 2, 4, 6, and 8. Pictures taken on day 8 allows the identification of a more neuronal-like morphology with axon-like projections. Images were taken at 10X magnification using a Leica DM IL inverted microscope with a Leica EC3 camera.

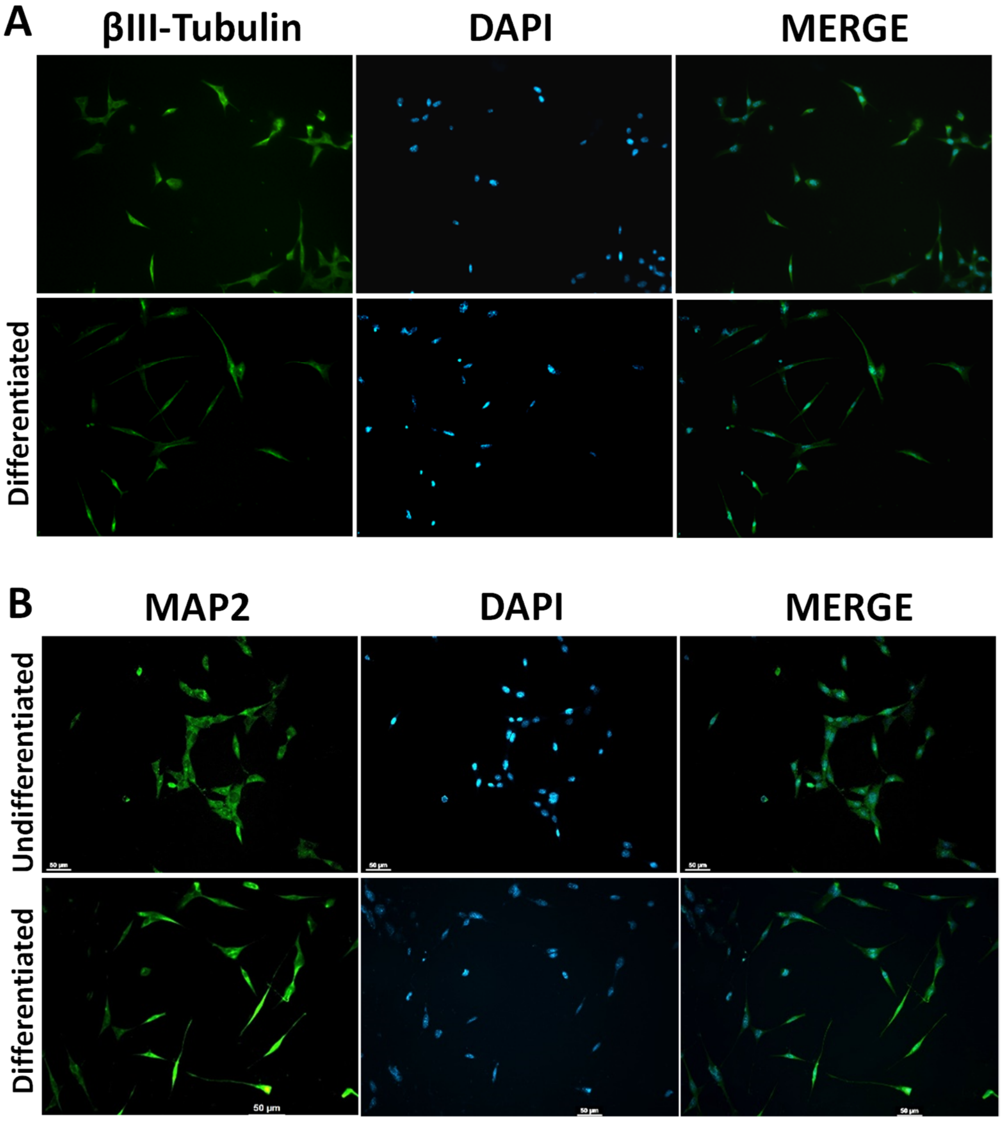

Figure S6. Fluorescence microscopy images of undifferentiated and differentiated SH-SY5Y cells using βIII-Tubulin and MAP2 to highlight their different morphology features. Images were obtained at 50X magnification.

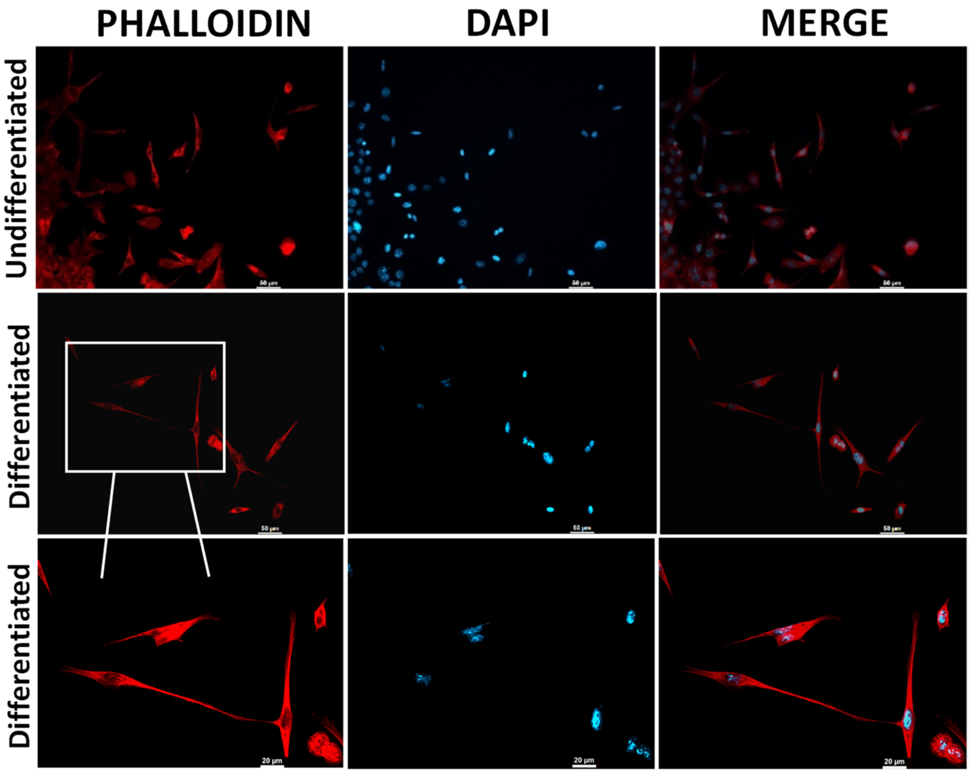

Figure S7. Staining of undifferentiated and differentiated SH-SY5Y cells with phalloidin and DAPI. Images were taken at 50 µm with a Leica DM5500 microscope.
